# Correction: Immunopeptidomics reveals determinants of *Mycobacterium tuberculosis* antigen presentation on MHC class I

**DOI:** 10.7554/eLife.108911

**Published:** 2025-08-20

**Authors:** Owen Leddy, Forest M White, Bryan D Bryson

**Keywords:** Other

Leddy O, White FM, Bryson BD. 2023. Immunopeptidomics reveals determinants of *Mycobacterium tuberculosis* antigen presentation on MHC class I. *eLife* **12**:e84070. doi: 10.7554/eLife.84070.

Published 19 April 2023

In the course of working on a related study, we realized that a coding mistake had caused some self (i.e., human protein derived) MHC-I peptides to be paired with the wrong source protein names. As a result, we also in some cases misstated which self peptides were derived from which subcellular locations. This error does not affect any of the *M. tuberculosis* peptides identified in the study, nor does it change any of our conclusions regarding processing and presentation of *M. tuberculosis* antigens. We sincerely apologize for this oversight and for any confusion this mistake may have caused.

Corrected text (in Results section):

Treatment with MG-132 and E64d each respectively reduced presentation of a subset of self MHC-I peptides but did not reduce presentation of *Mtb* peptides (Figure 4C).

Original text:

Treatment with MG-132 reduced presentation of MHC-I peptides derived from cytosolic or nuclear host proteins, but not peptides derived from Mtb proteins or secreted or endomembrane compartment localized host proteins (Figure 4C). Treatment with E64d decreased presentation of peptides derived from endosomal or secreted proteins and had a range of effects on peptides derived from cytosolic or nuclear proteins, but did not decrease presentation of Mtb peptides (Figure 4C).

Removed text (from “Quantitative analyses” Methods section):

Self peptides were classified as derived from cytosolic/nuclear proteins or extracellular/endosomal proteins based on Uniprot annotations of the source protein(s). Peptides derived from transmembrane proteins were classified based on which side of the membrane they were predicted to reside on based on topological predictions made using DeepTMHMM (Hallgren et al., 2022).

Removed reference:

Hallgren J., Tsirigos KD., Pedersen MD., Almagro Armenteros JJ., Marcatili P., Nielsen H., Krogh A., Winther O (2022) DeepTMHMM Predicts Alpha and Beta Transmembrane Proteins Using Deep Neural Networks. bioRxiv. https://doi.org/10.1101/2022.04.08.487609

The corrected Figure 4 (with panel C updated) is shown here:

**Figure 1:**
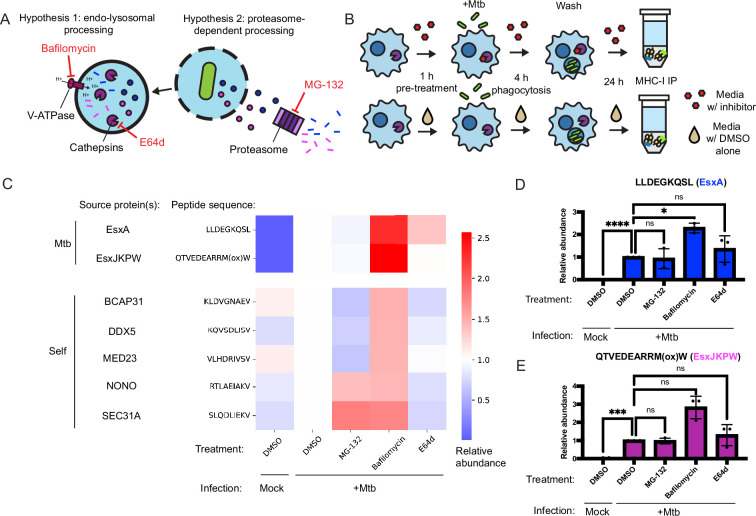


The originally published Figure 4 is shown for reference:

**Figure 2:**
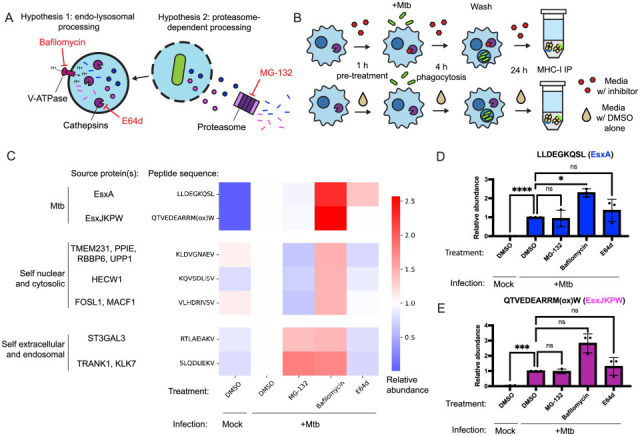


The article has been corrected accordingly.

