# Supplementary figures and images for: Immunopeptidomics reveals determinants of Mycobacterium tuberculosis antigen presentation on MHC class I (part 2 of 2)
Source: eLife. 2023 Apr 19;12:e84070. doi: 10.7554/eLife.84070 (PMC10159623; doi:10.7554/eLife.84070)

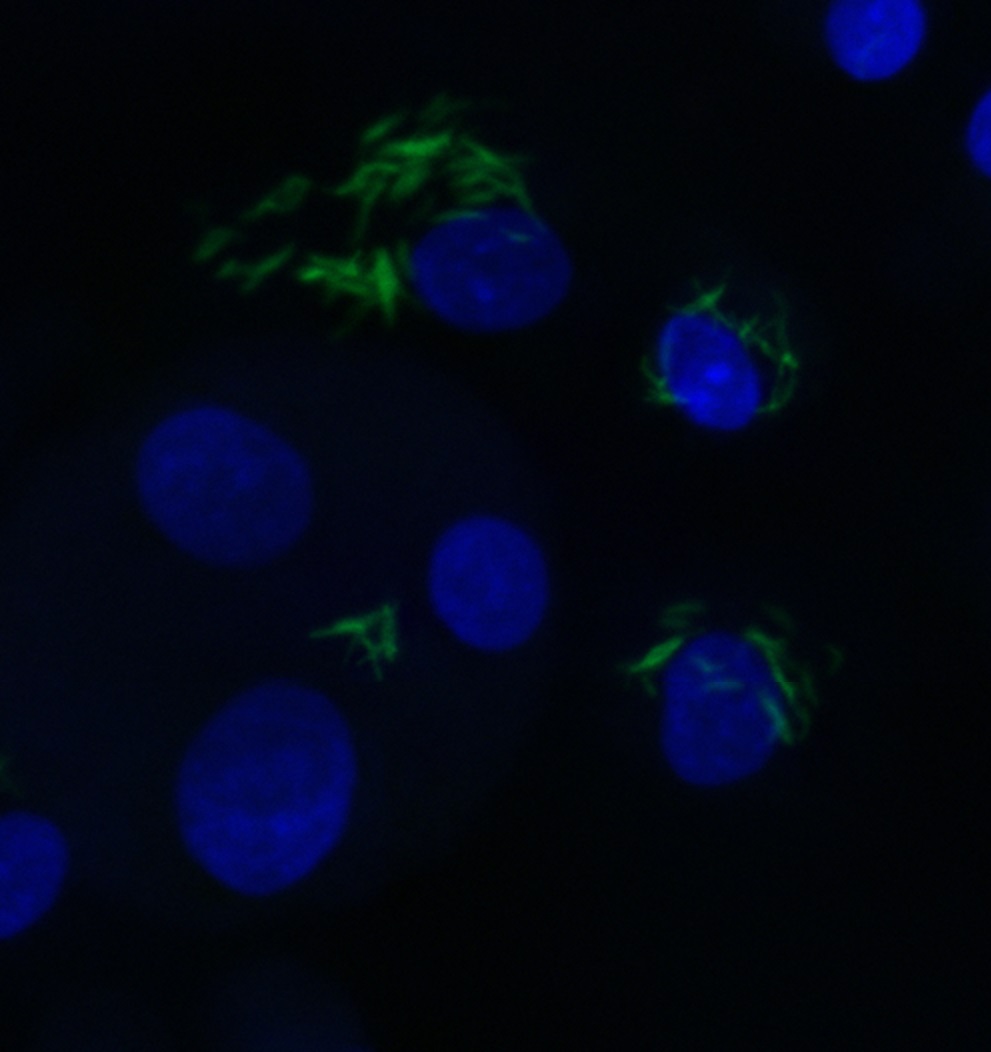

Supplement: Figure 2—source data 1. [file elife-84070-fig2-data1.zip › Figure 2 source data 1/Secondary ctrl/LAMP-1 secondary only.JPG]

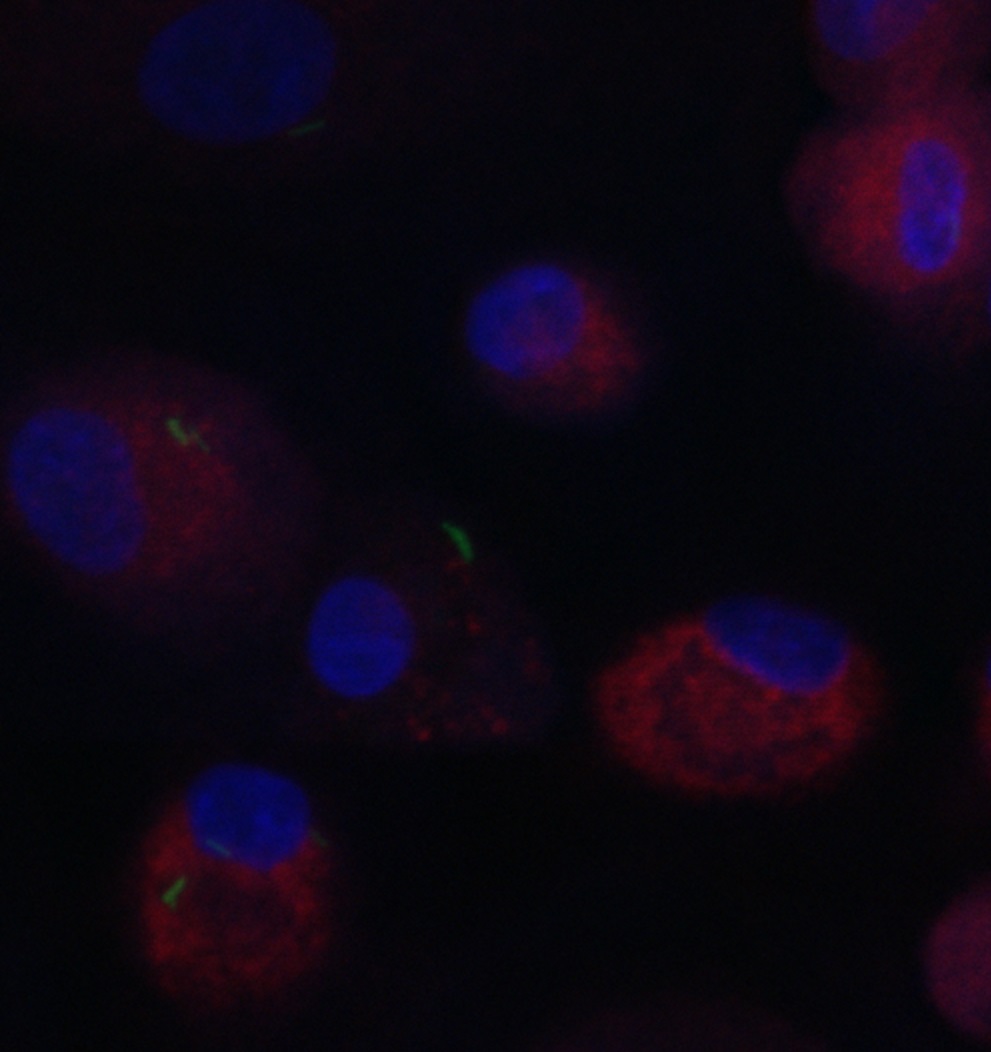

Supplement: Figure 2—source data 1. [file elife-84070-fig2-data1.zip › Figure 2 source data 1/Secondary ctrl/MHC-I +primary.JPG]

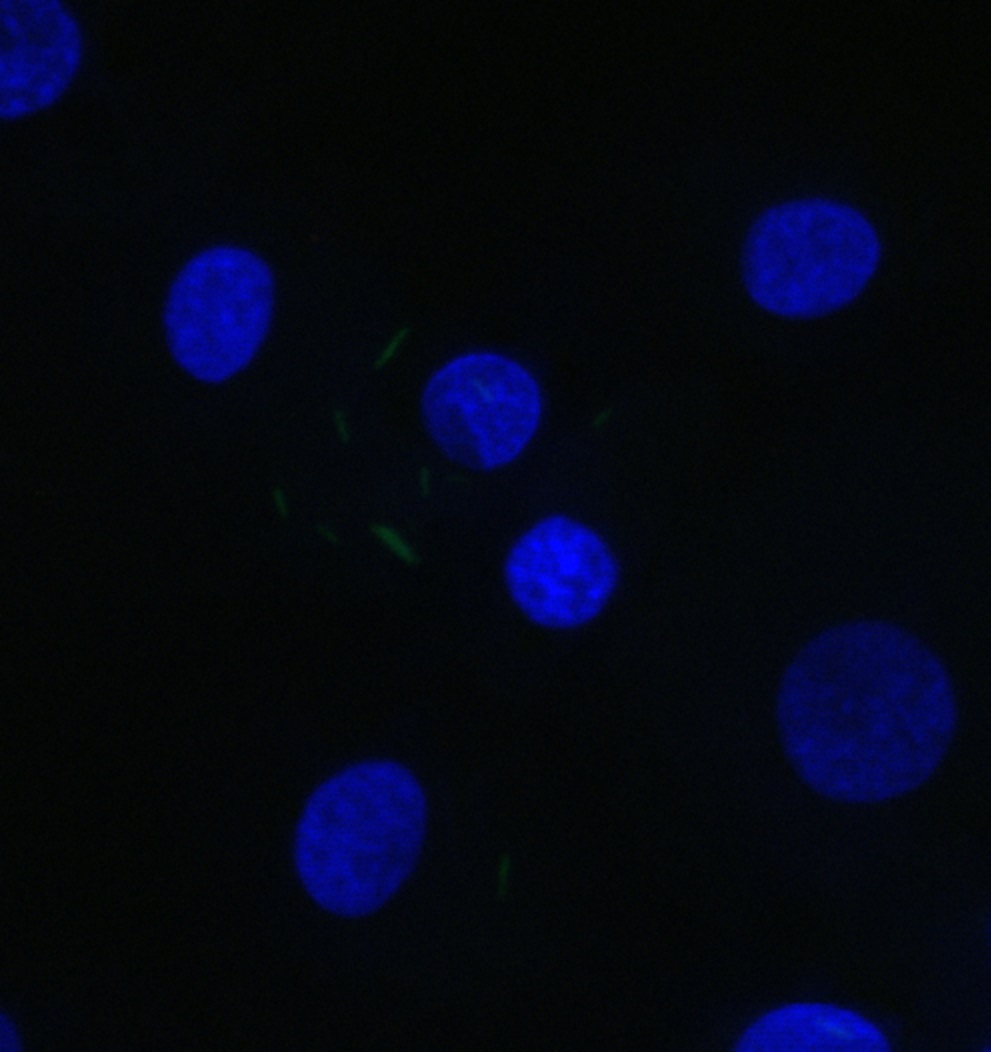

Supplement: Figure 2—source data 1. [file elife-84070-fig2-data1.zip › Figure 2 source data 1/Secondary ctrl/MHC-I secondary only.JPG]

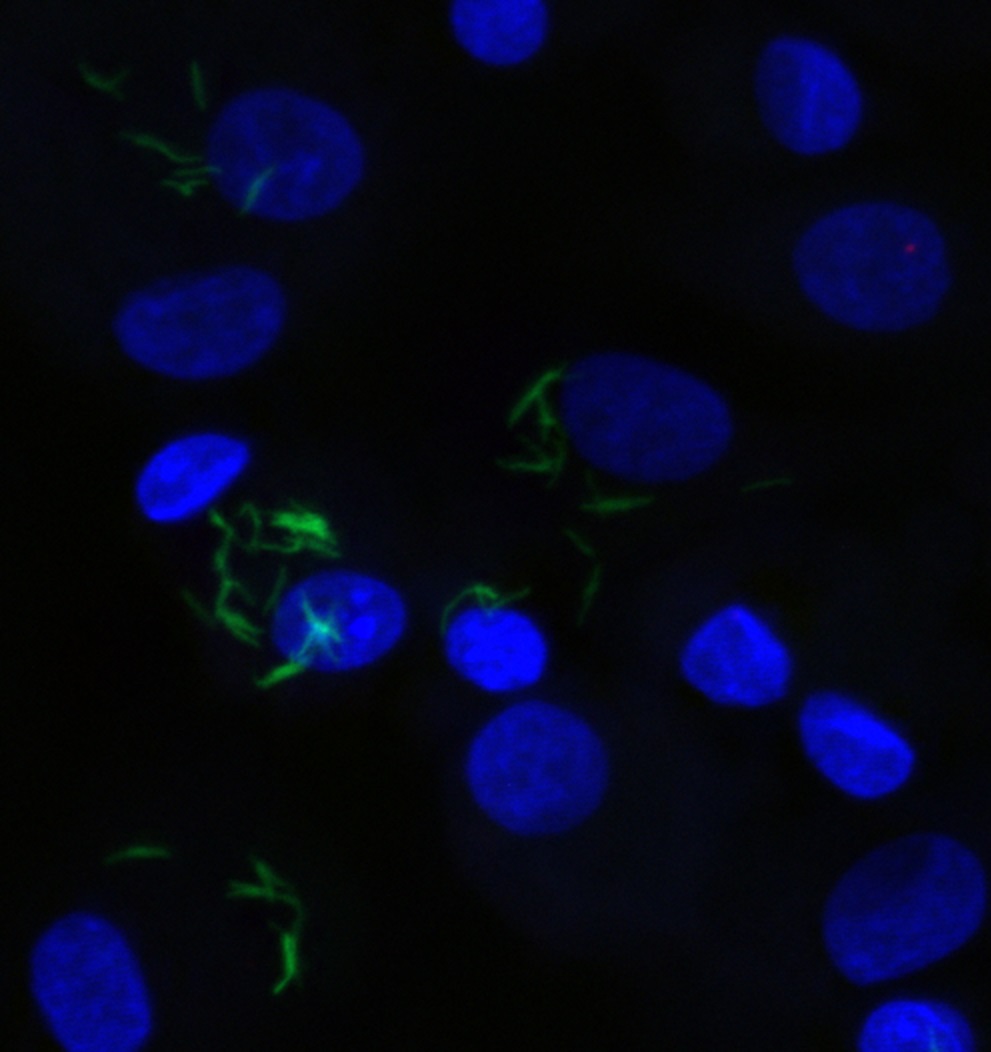

Supplement: Figure 2—source data 1. [file elife-84070-fig2-data1.zip › Figure 2 source data 1/Secondary ctrl/P62 secondary only.JPG]

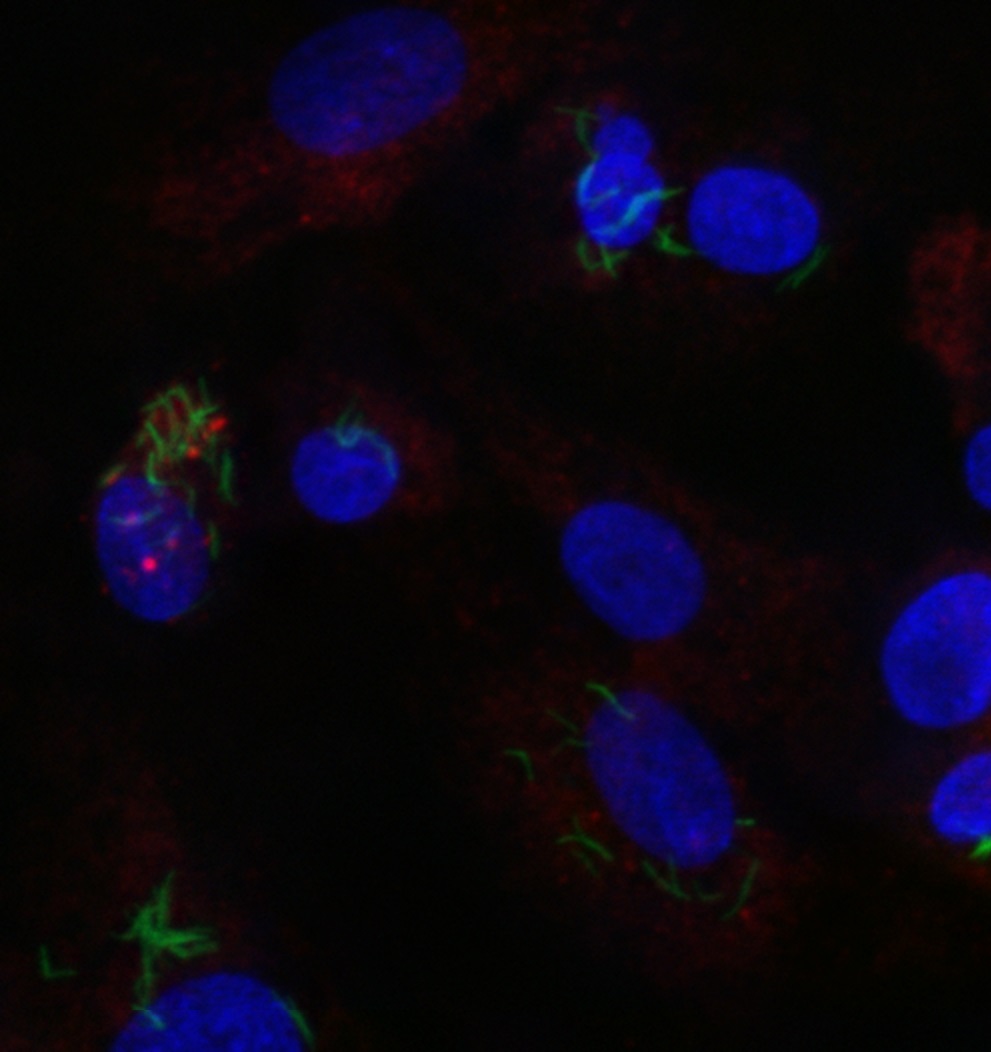

Supplement: Figure 2—source data 1. [file elife-84070-fig2-data1.zip › Figure 2 source data 1/Secondary ctrl/P62+primary.JPG]

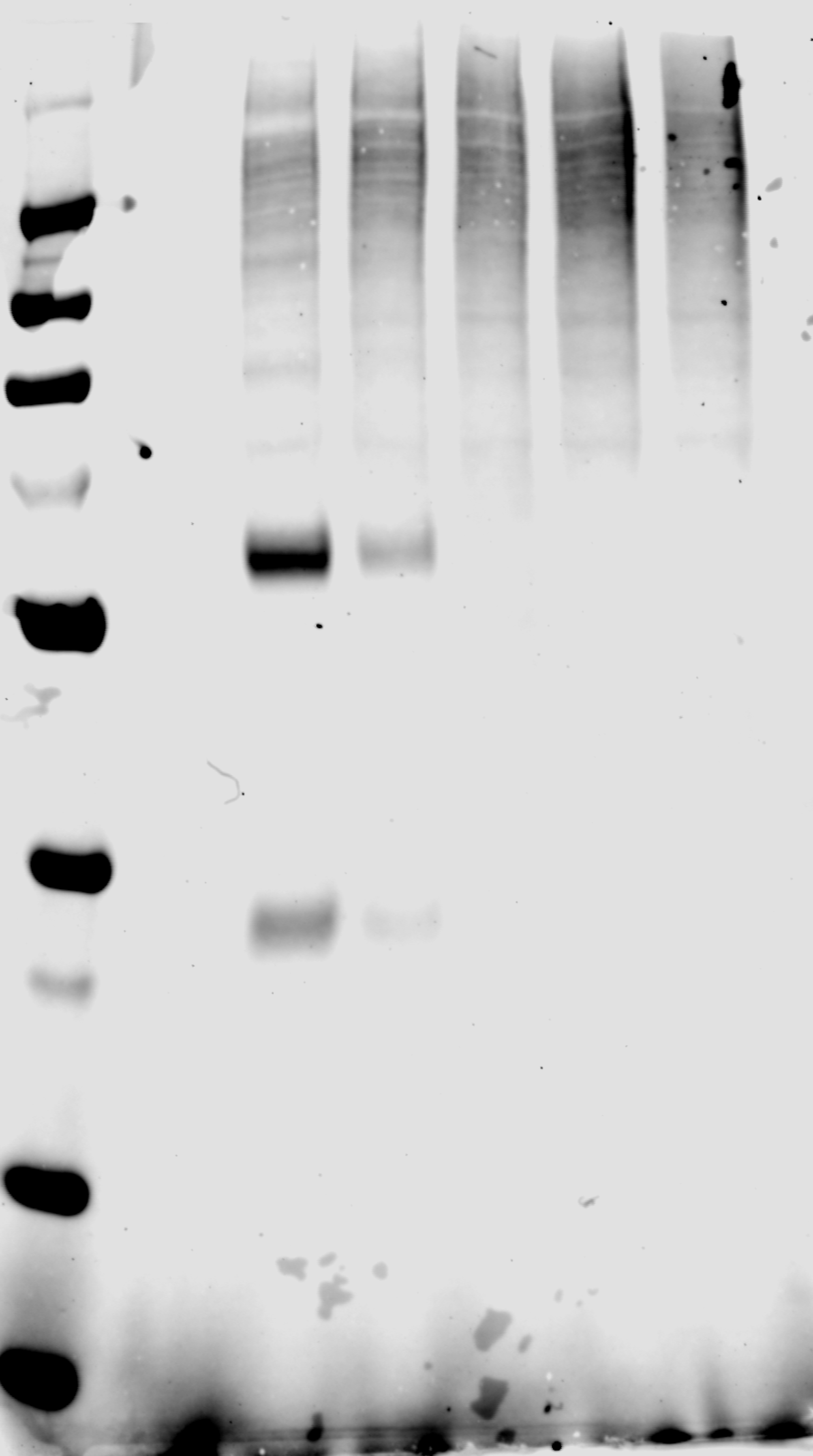

Supplement: Figure 4—figure supplement 1—source data 1. [file elife-84070-fig4-figsupp1-data1.zip › Figure 4 - figure supplement 1 - source data 1/Donor_A_K48Ub.tif]

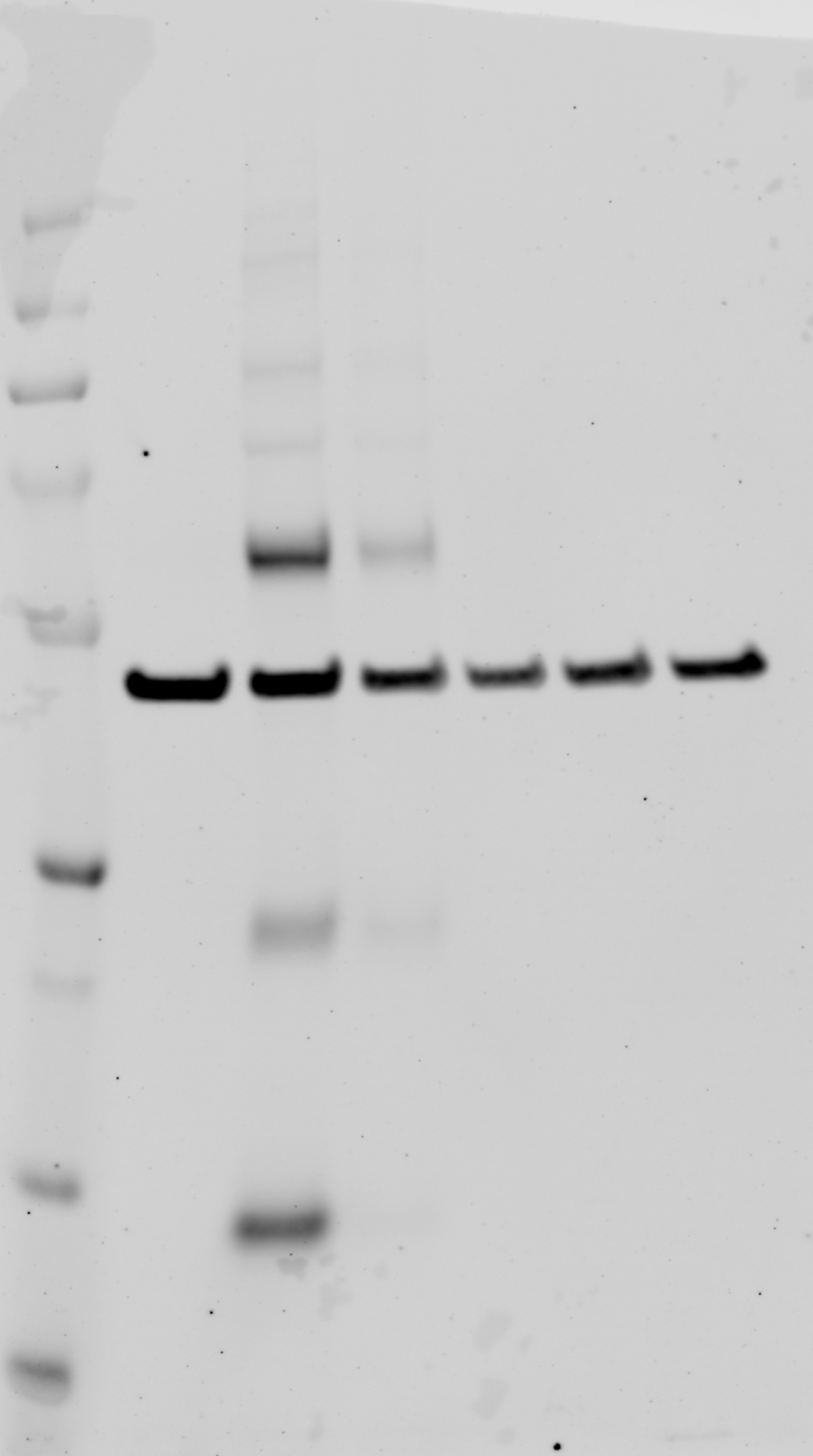

Supplement: Figure 4—figure supplement 1—source data 1. [file elife-84070-fig4-figsupp1-data1.zip › Figure 4 - figure supplement 1 - source data 1/Donor_A_actin.tif]

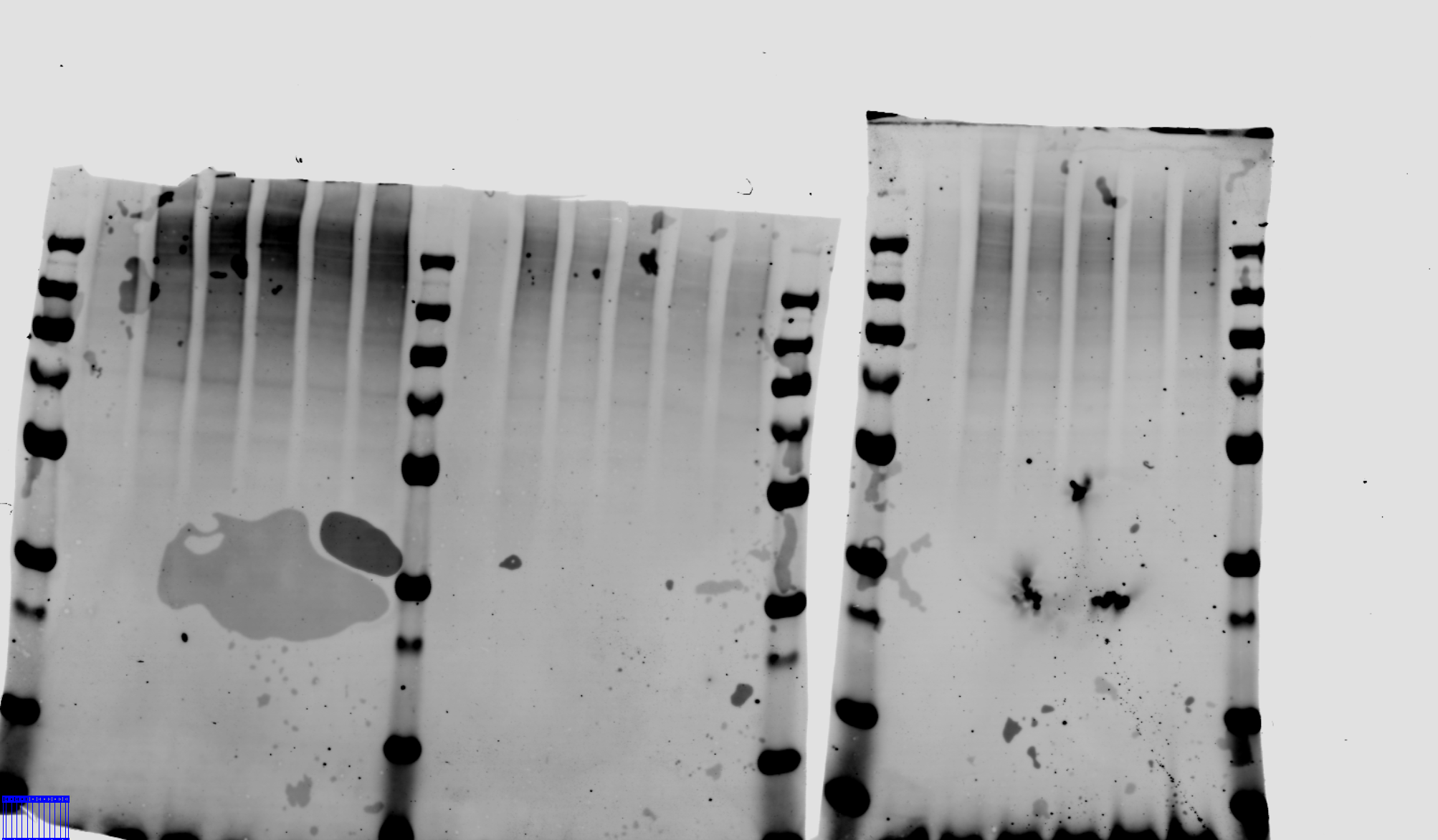

Supplement: Figure 4—figure supplement 1—source data 1. [file elife-84070-fig4-figsupp1-data1.zip › Figure 4 - figure supplement 1 - source data 1/Mtb_K48Ub.tif]

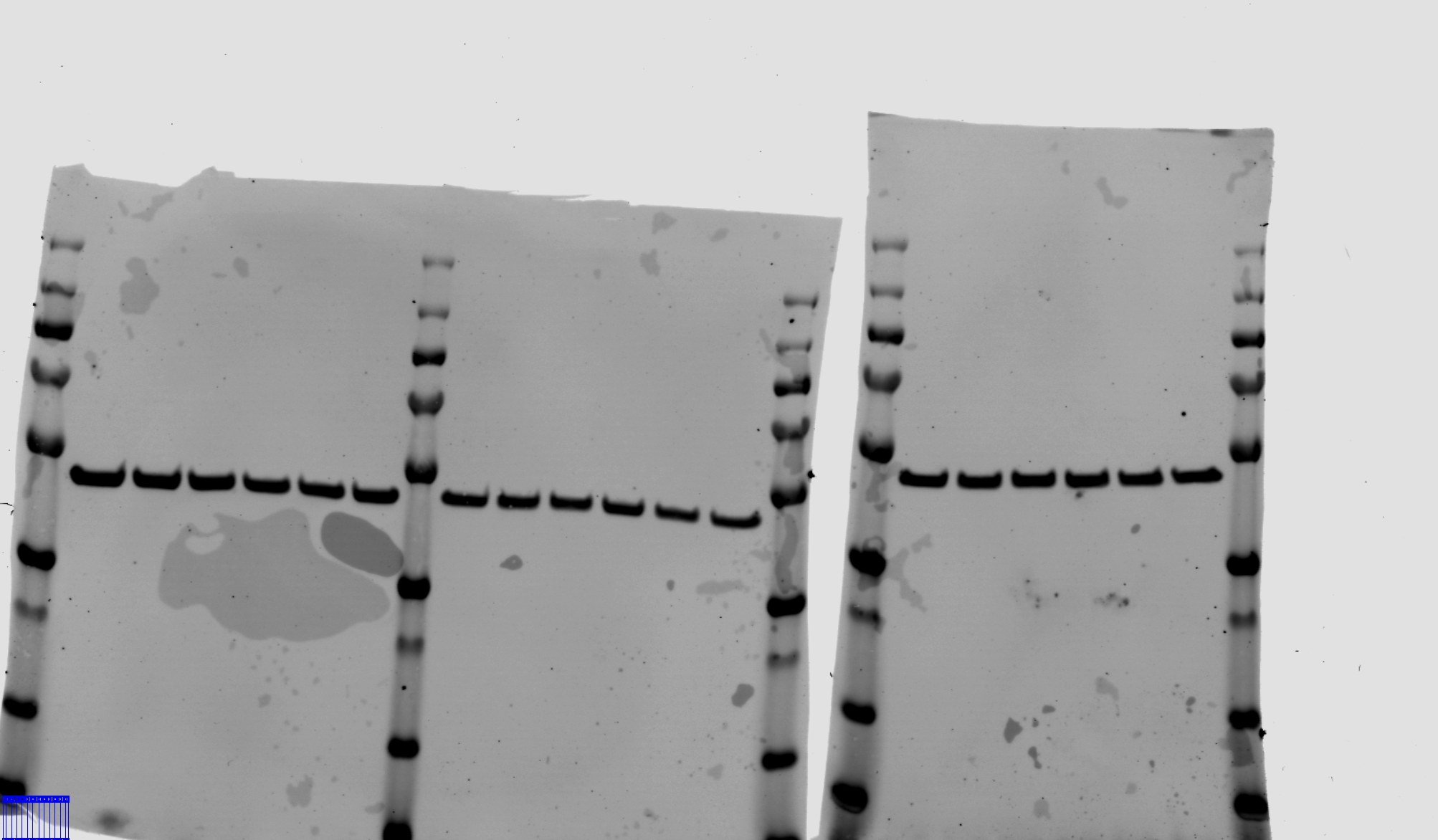

Supplement: Figure 4—figure supplement 1—source data 1. [file elife-84070-fig4-figsupp1-data1.zip › Figure 4 - figure supplement 1 - source data 1/Mtb_actin.tif]

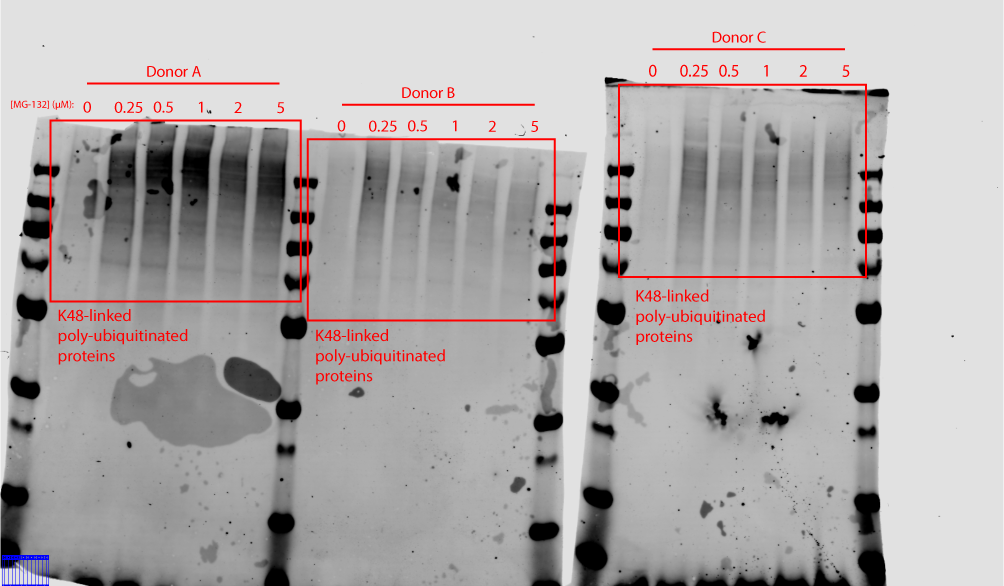

Supplement: Figure 4—figure supplement 1—source data 1. [file elife-84070-fig4-figsupp1-data1.zip › Figure 4 - figure supplement 1 - source data 1/Mtb_K48Ub_annotated.tif]

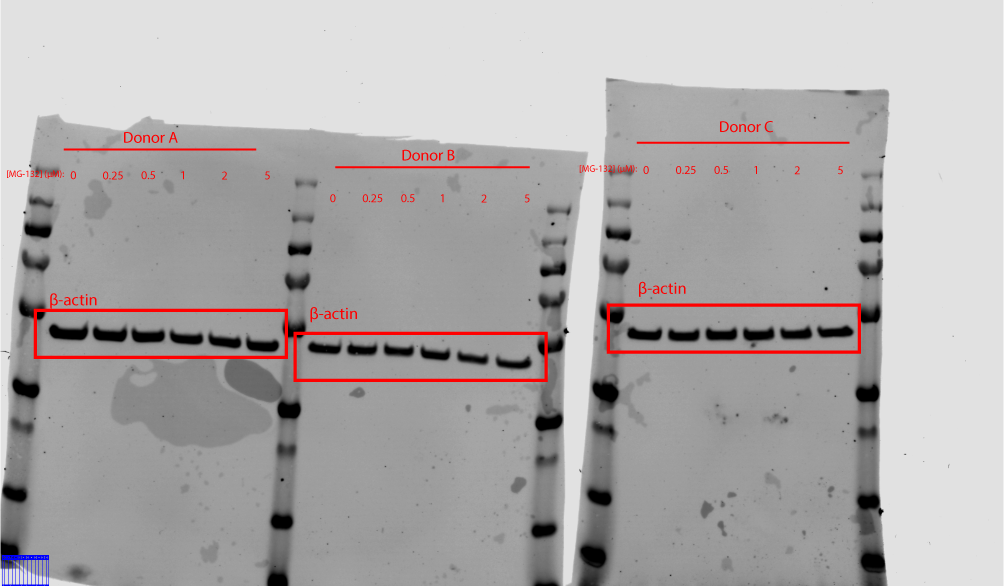

Supplement: Figure 4—figure supplement 1—source data 1. [file elife-84070-fig4-figsupp1-data1.zip › Figure 4 - figure supplement 1 - source data 1/Mtb_actin_annotated.tif]

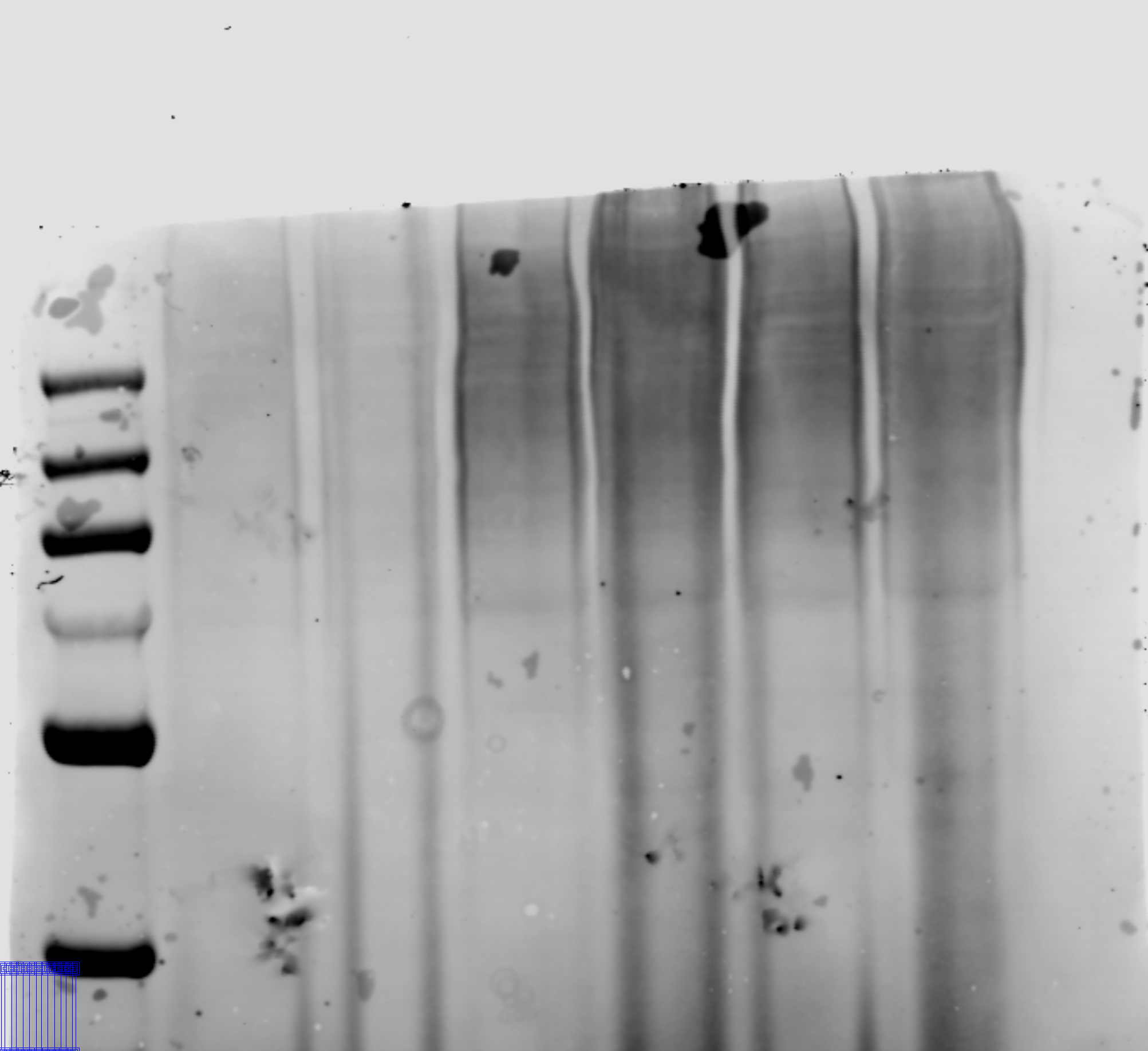

Supplement: Figure 4—figure supplement 1—source data 1. [file elife-84070-fig4-figsupp1-data1.zip › Figure 4 - figure supplement 1 - source data 1/Donor_C_K48Ub.tif]

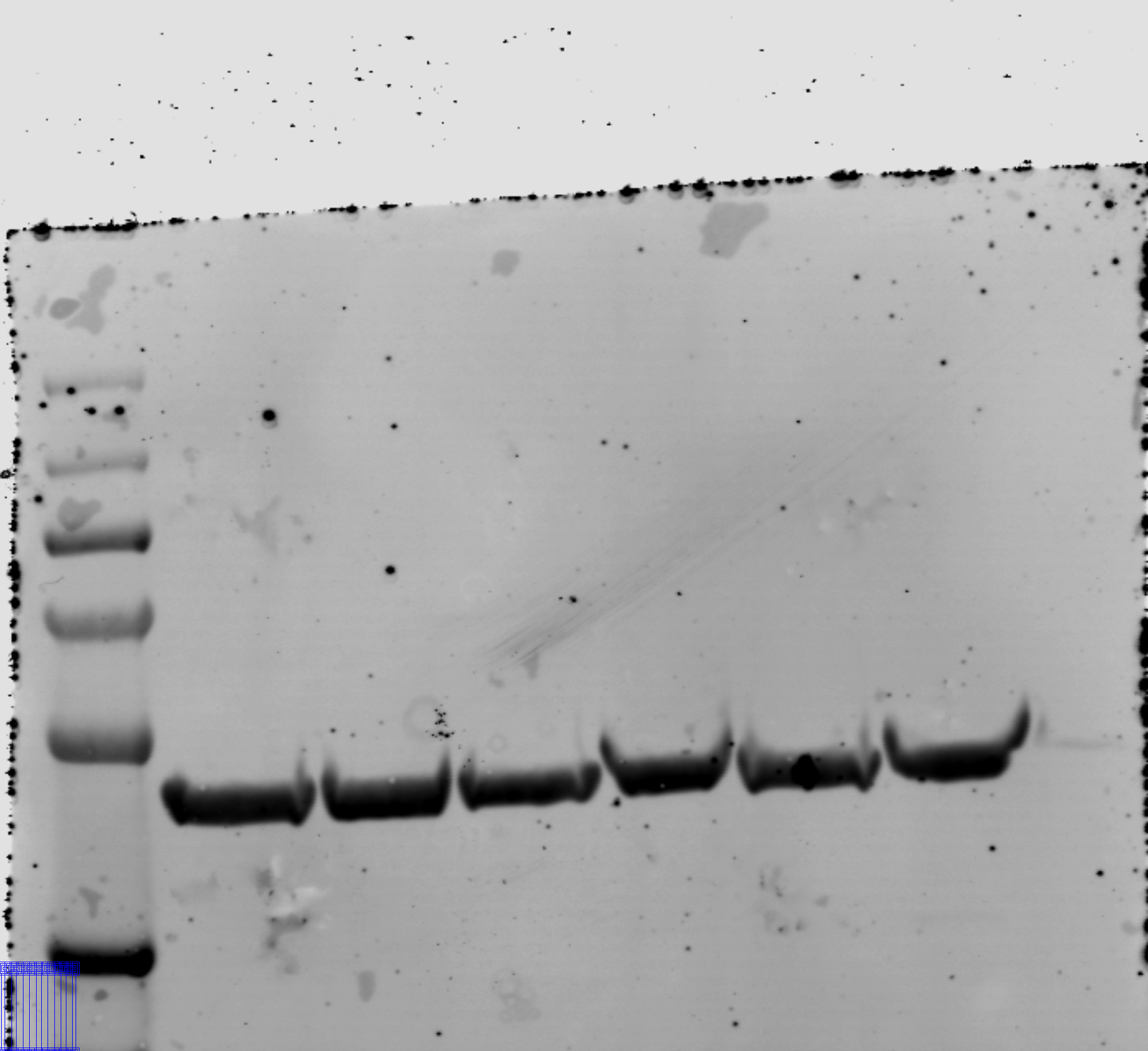

Supplement: Figure 4—figure supplement 1—source data 1. [file elife-84070-fig4-figsupp1-data1.zip › Figure 4 - figure supplement 1 - source data 1/Donor_C_actin.tif]

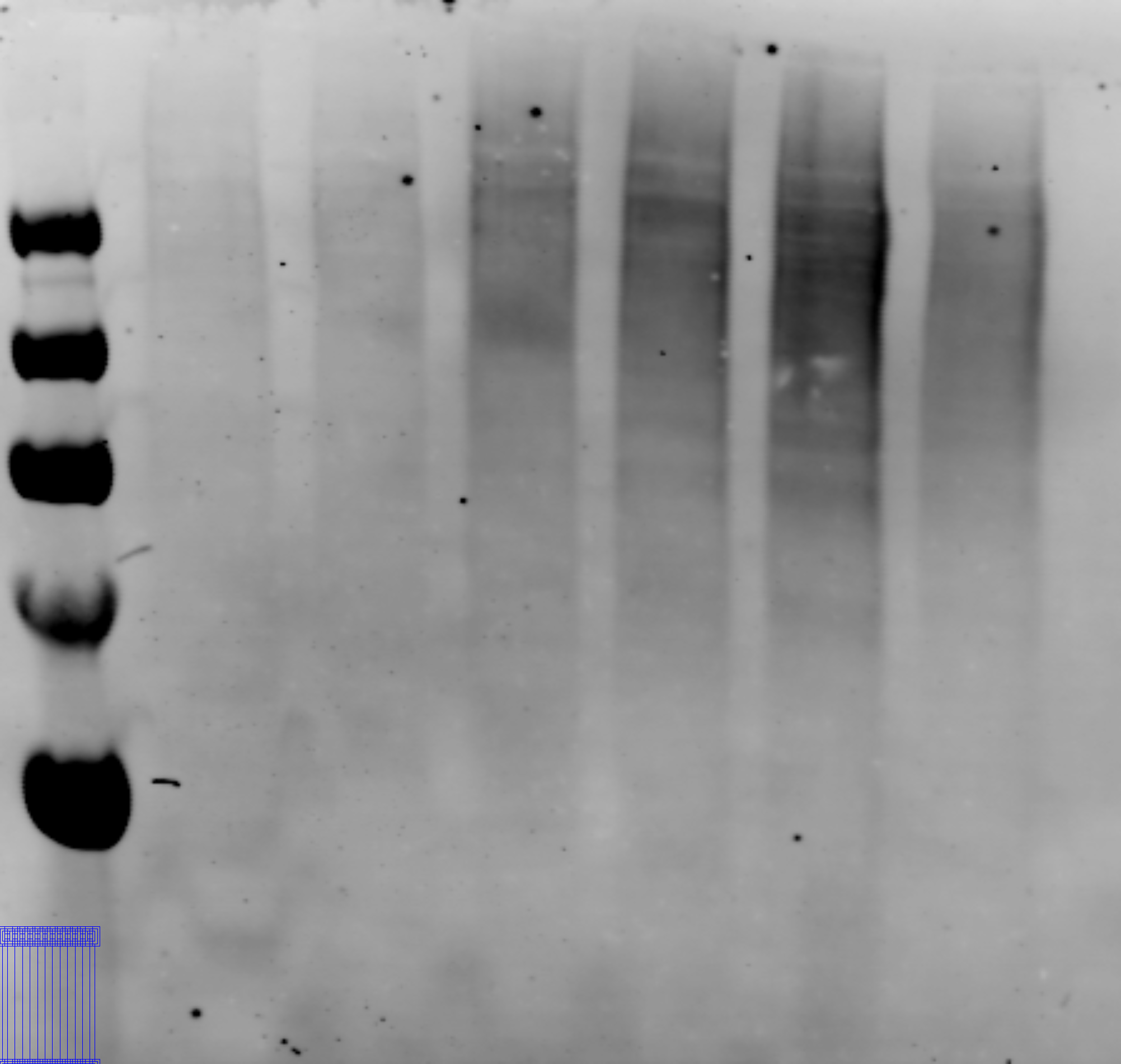

Supplement: Figure 4—figure supplement 1—source data 1. [file elife-84070-fig4-figsupp1-data1.zip › Figure 4 - figure supplement 1 - source data 1/Donor_B_K48Ub.tif]

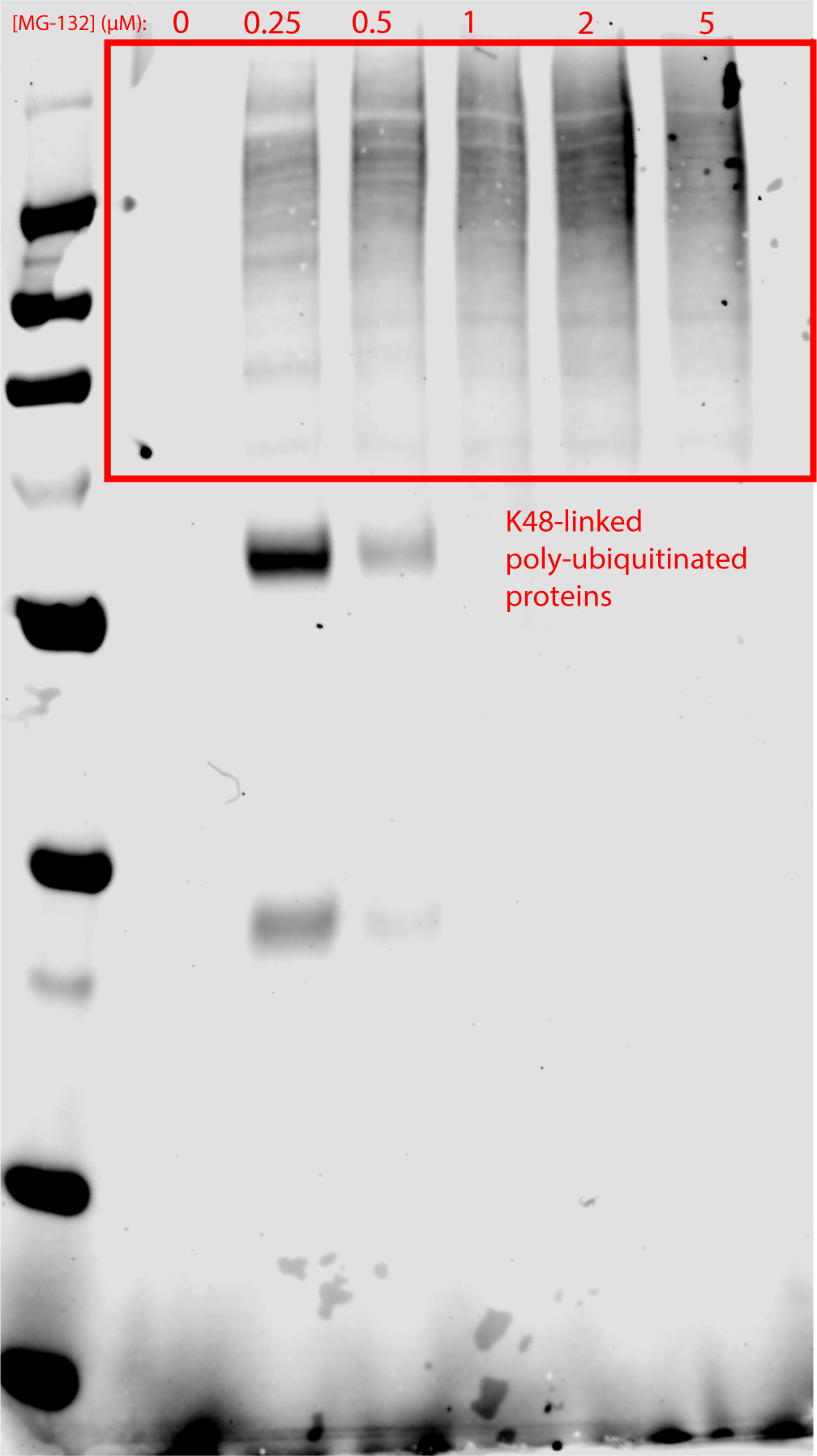

Supplement: Figure 4—figure supplement 1—source data 1. [file elife-84070-fig4-figsupp1-data1.zip › Figure 4 - figure supplement 1 - source data 1/Donor_A_K48Ub_annotated.tif]

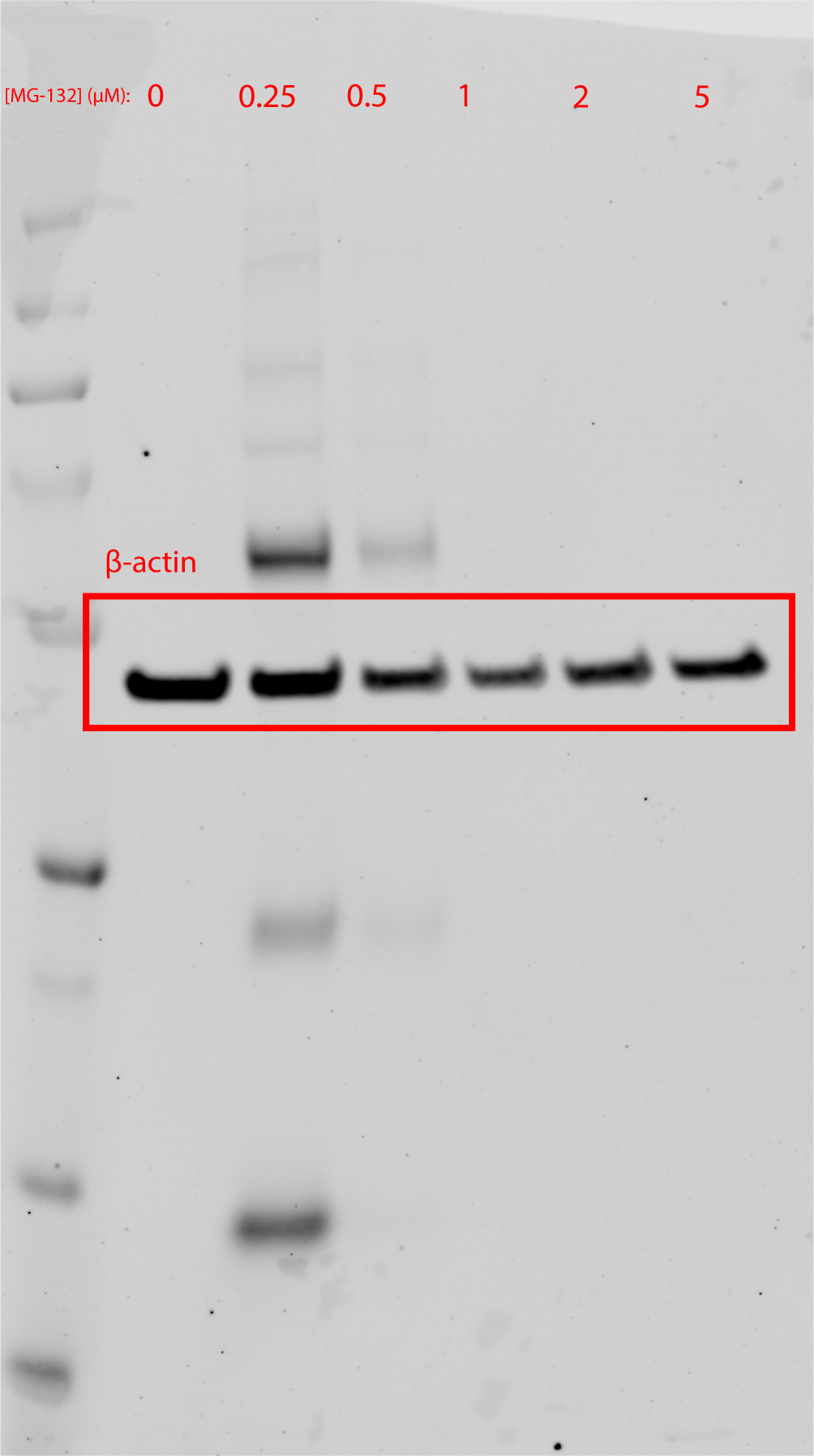

Supplement: Figure 4—figure supplement 1—source data 1. [file elife-84070-fig4-figsupp1-data1.zip › Figure 4 - figure supplement 1 - source data 1/Donor_A_actin_annotated.tif]

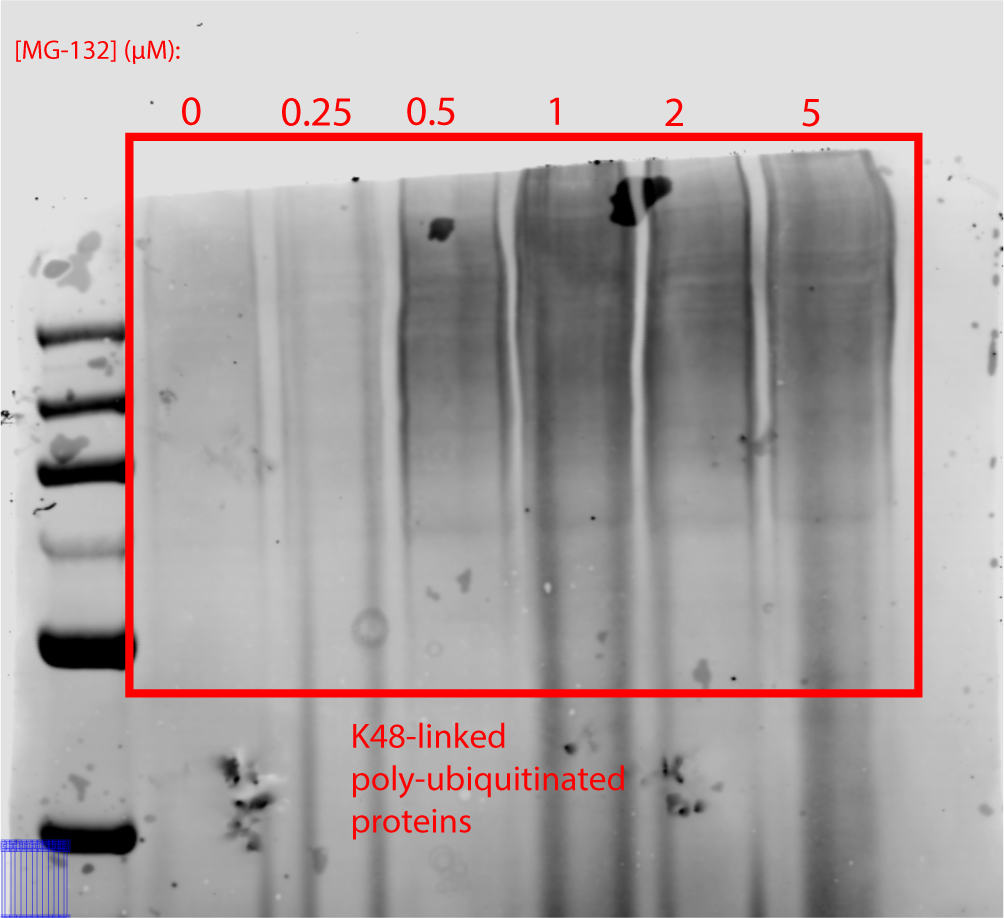

Supplement: Figure 4—figure supplement 1—source data 1. [file elife-84070-fig4-figsupp1-data1.zip › Figure 4 - figure supplement 1 - source data 1/Donor_C_K48Ub_annotated.tif]

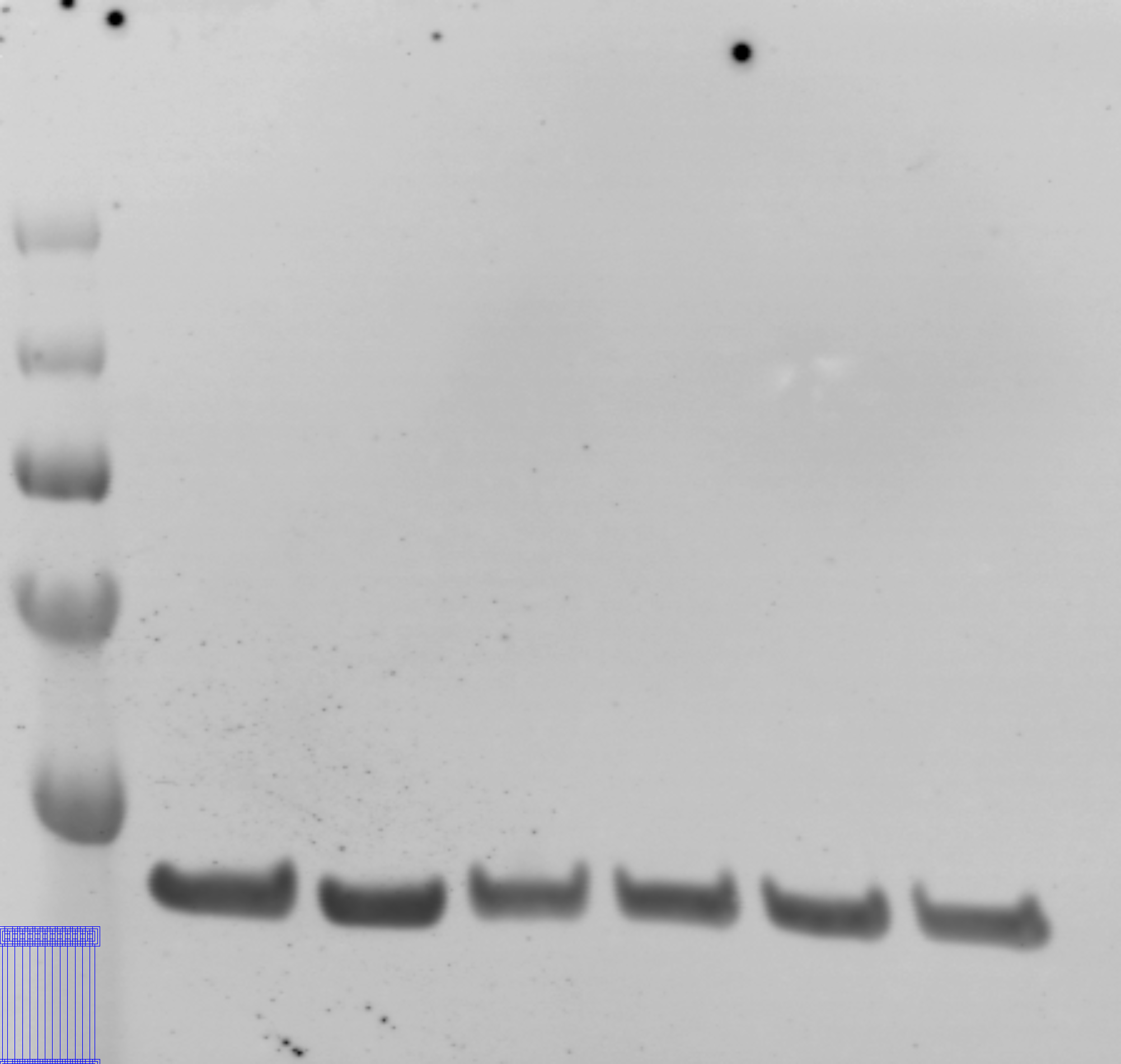

Supplement: Figure 4—figure supplement 1—source data 1. [file elife-84070-fig4-figsupp1-data1.zip › Figure 4 - figure supplement 1 - source data 1/Donor_B_actin.tif]

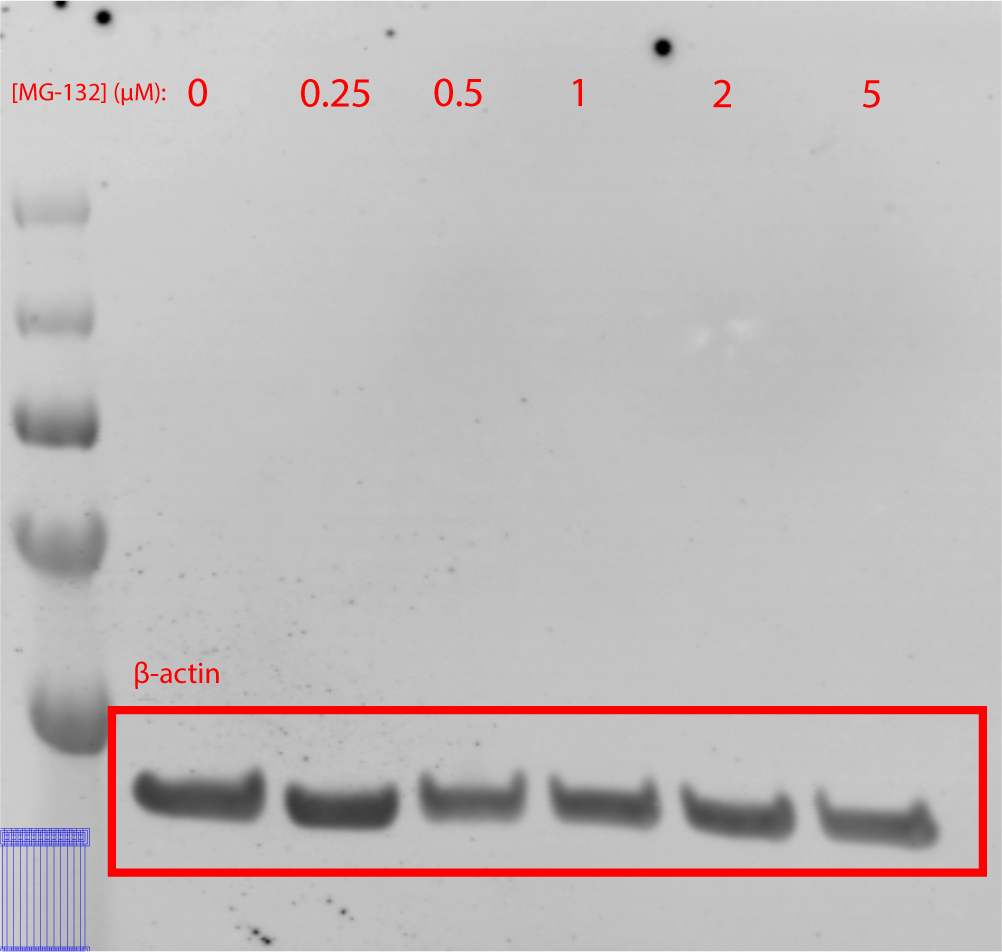

Supplement: Figure 4—figure supplement 1—source data 1. [file elife-84070-fig4-figsupp1-data1.zip › Figure 4 - figure supplement 1 - source data 1/Donor_B_actin_annotated.tif]

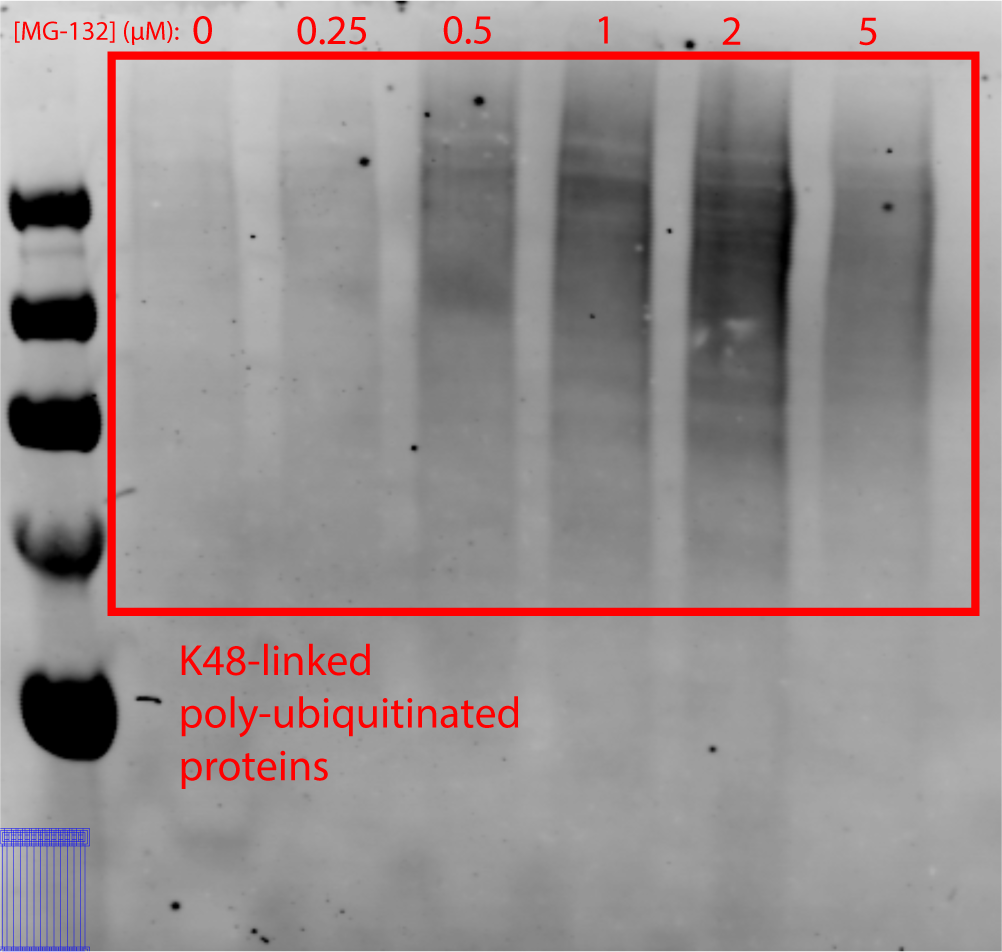

Supplement: Figure 4—figure supplement 1—source data 1. [file elife-84070-fig4-figsupp1-data1.zip › Figure 4 - figure supplement 1 - source data 1/Donor_B_K48Ub_annotated.tif]

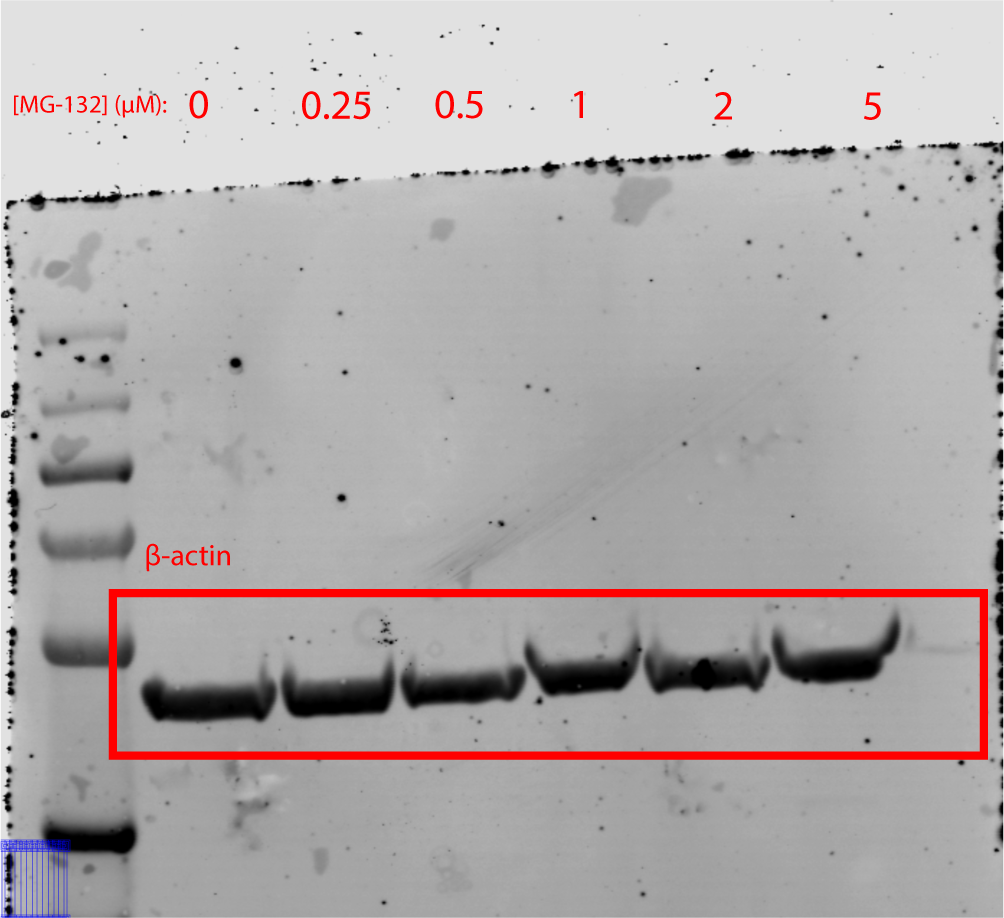

Supplement: Figure 4—figure supplement 1—source data 1. [file elife-84070-fig4-figsupp1-data1.zip › Figure 4 - figure supplement 1 - source data 1/Donor_C_actin_annotated.tif]
