# Supplementary figures and images for: Immunopeptidomics reveals determinants of Mycobacterium tuberculosis antigen presentation on MHC class I (part 1 of 2)
Source: eLife. 2023 Apr 19;12:e84070. doi: 10.7554/eLife.84070 (PMC10159623; doi:10.7554/eLife.84070)

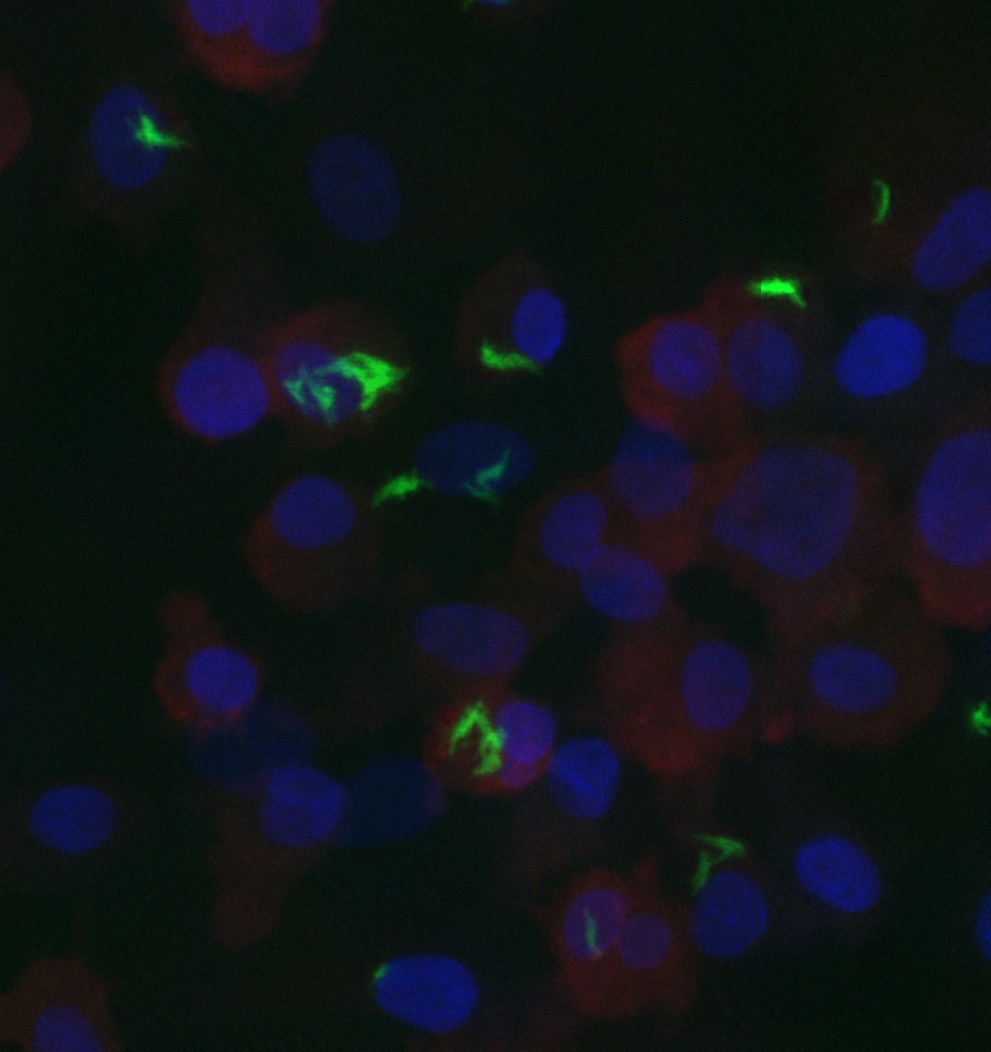

Supplement: Figure 2—source data 1. [file elife-84070-fig2-data1.zip › Figure 2 source data 1/Galectin-3/Galectin-3 ESX 120h.JPG]

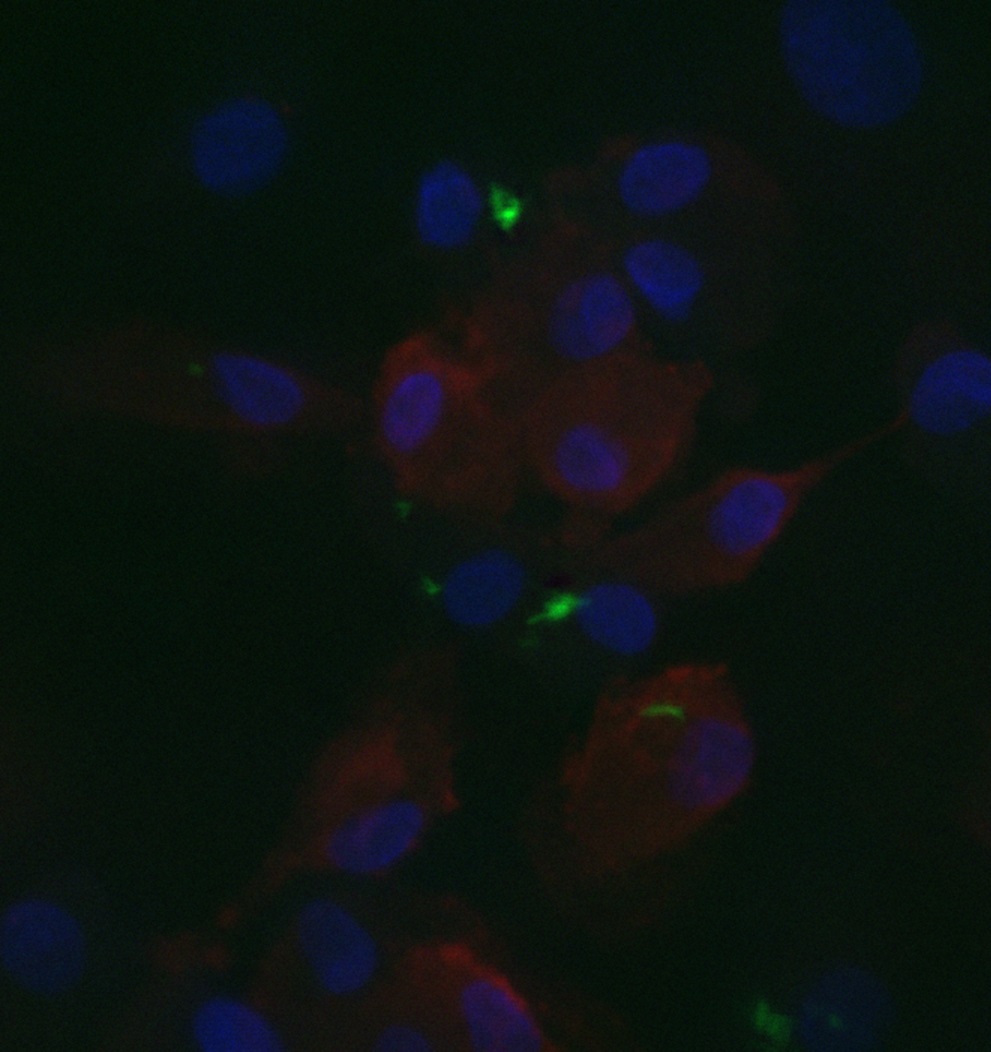

Supplement: Figure 2—source data 1. [file elife-84070-fig2-data1.zip › Figure 2 source data 1/Galectin-3/Galectin-3 ESX 24h.JPG]

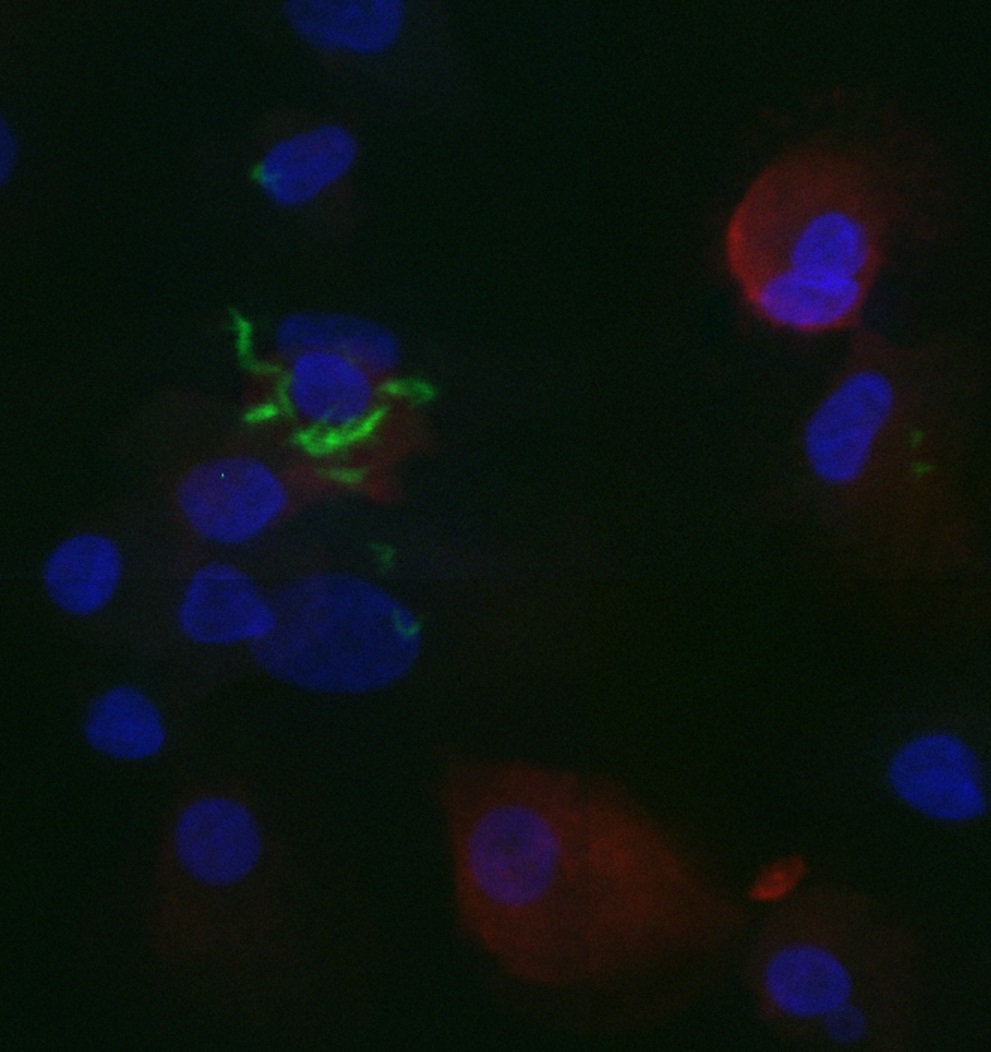

Supplement: Figure 2—source data 1. [file elife-84070-fig2-data1.zip › Figure 2 source data 1/Galectin-3/Galectin-3 ESX 72h.JPG]

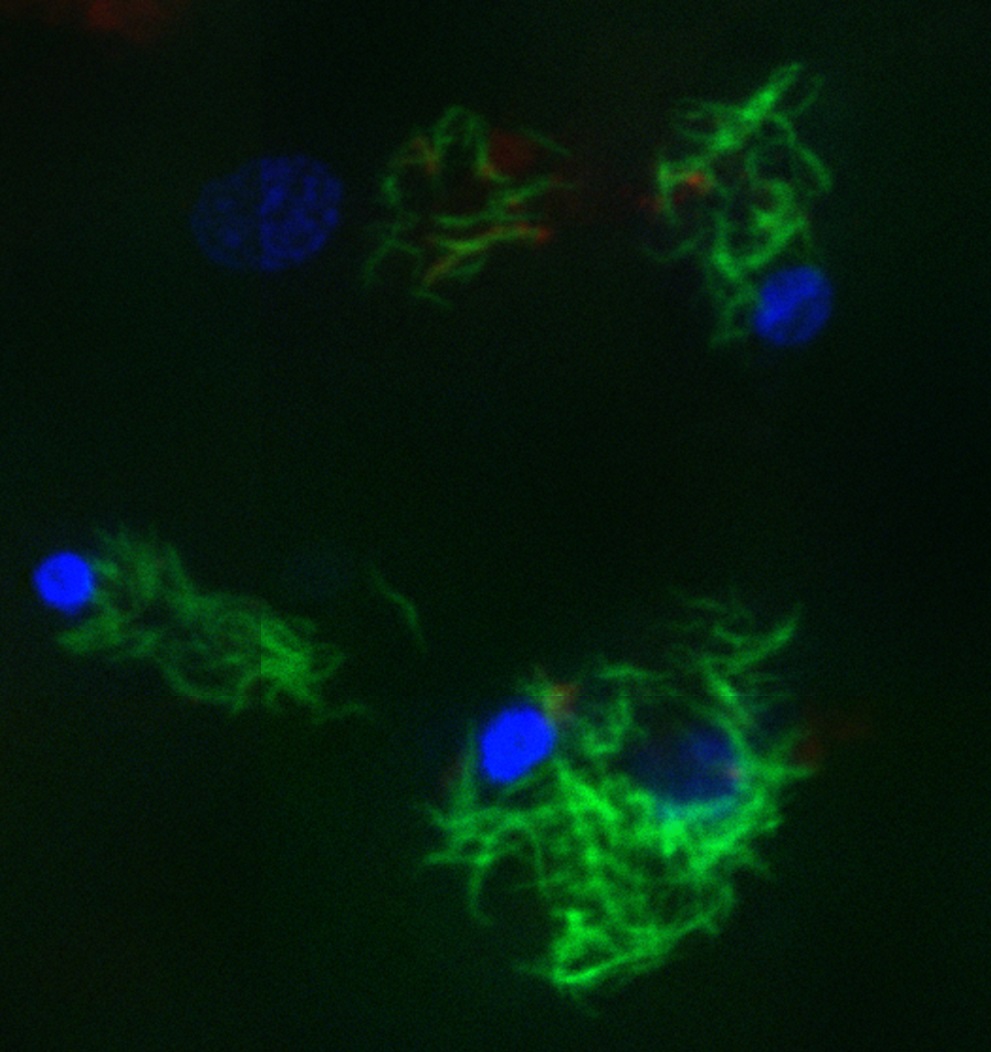

Supplement: Figure 2—source data 1. [file elife-84070-fig2-data1.zip › Figure 2 source data 1/Galectin-3/Galectin-3 WT 120h 2.JPG]

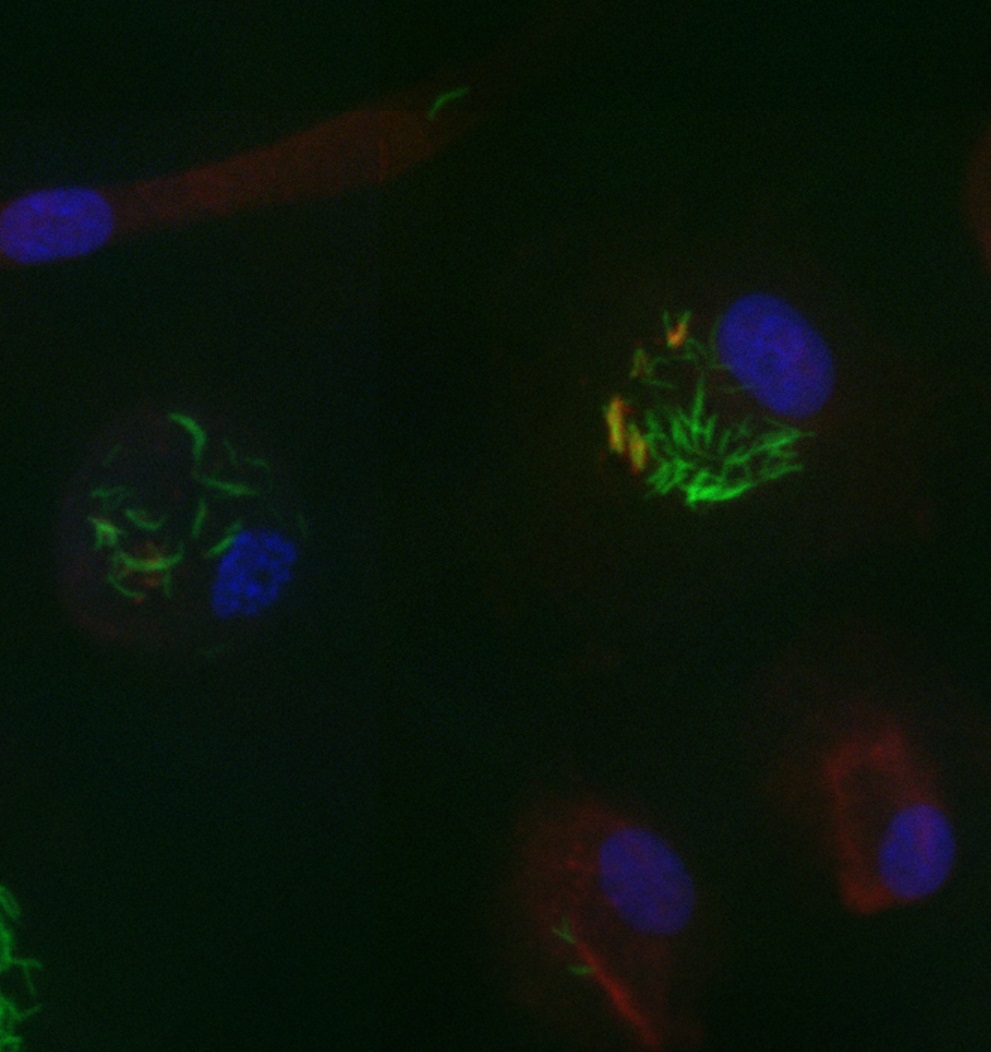

Supplement: Figure 2—source data 1. [file elife-84070-fig2-data1.zip › Figure 2 source data 1/Galectin-3/Galectin-3 WT 120h.JPG]

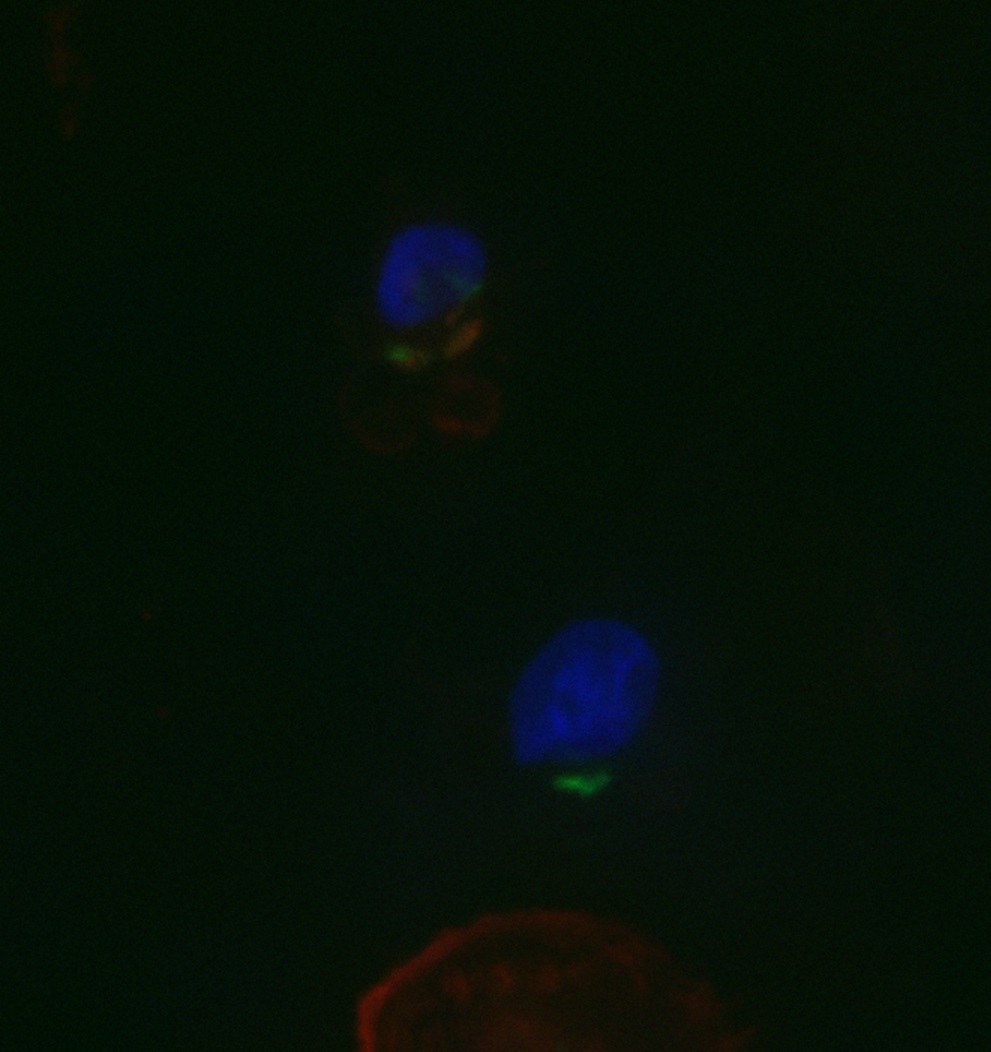

Supplement: Figure 2—source data 1. [file elife-84070-fig2-data1.zip › Figure 2 source data 1/Galectin-3/Galectin-3 WT 24h 2.JPG]

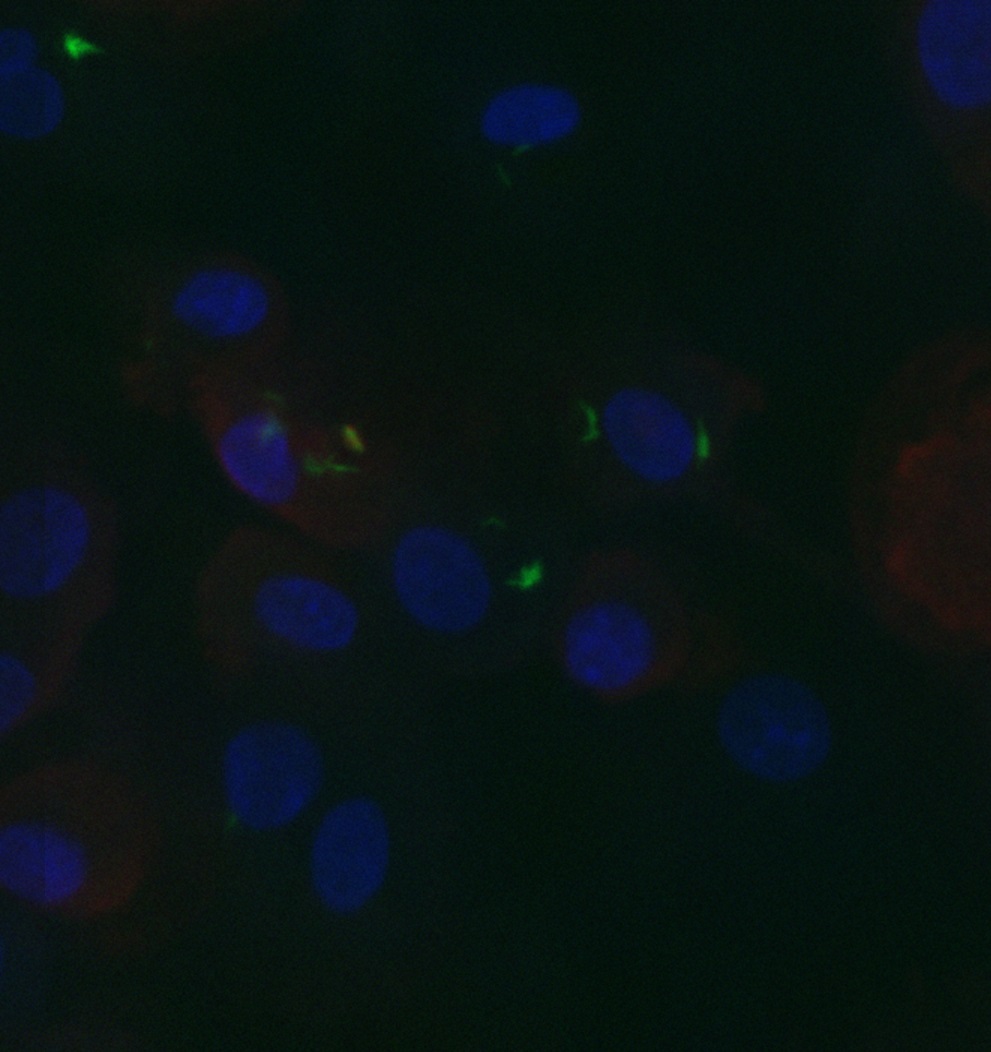

Supplement: Figure 2—source data 1. [file elife-84070-fig2-data1.zip › Figure 2 source data 1/Galectin-3/Galectin-3 WT 24h.JPG]

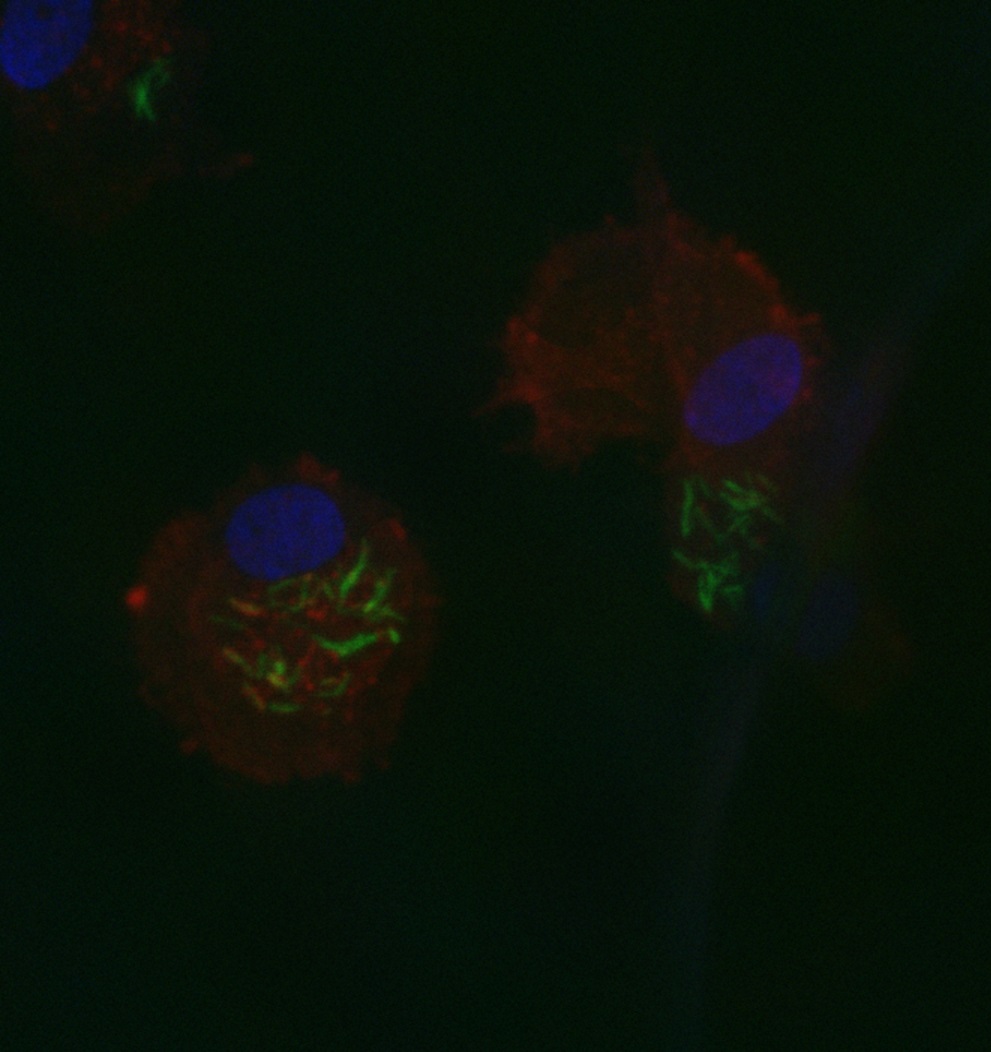

Supplement: Figure 2—source data 1. [file elife-84070-fig2-data1.zip › Figure 2 source data 1/Galectin-3/Galectin-3 WT 72h 2.JPG]

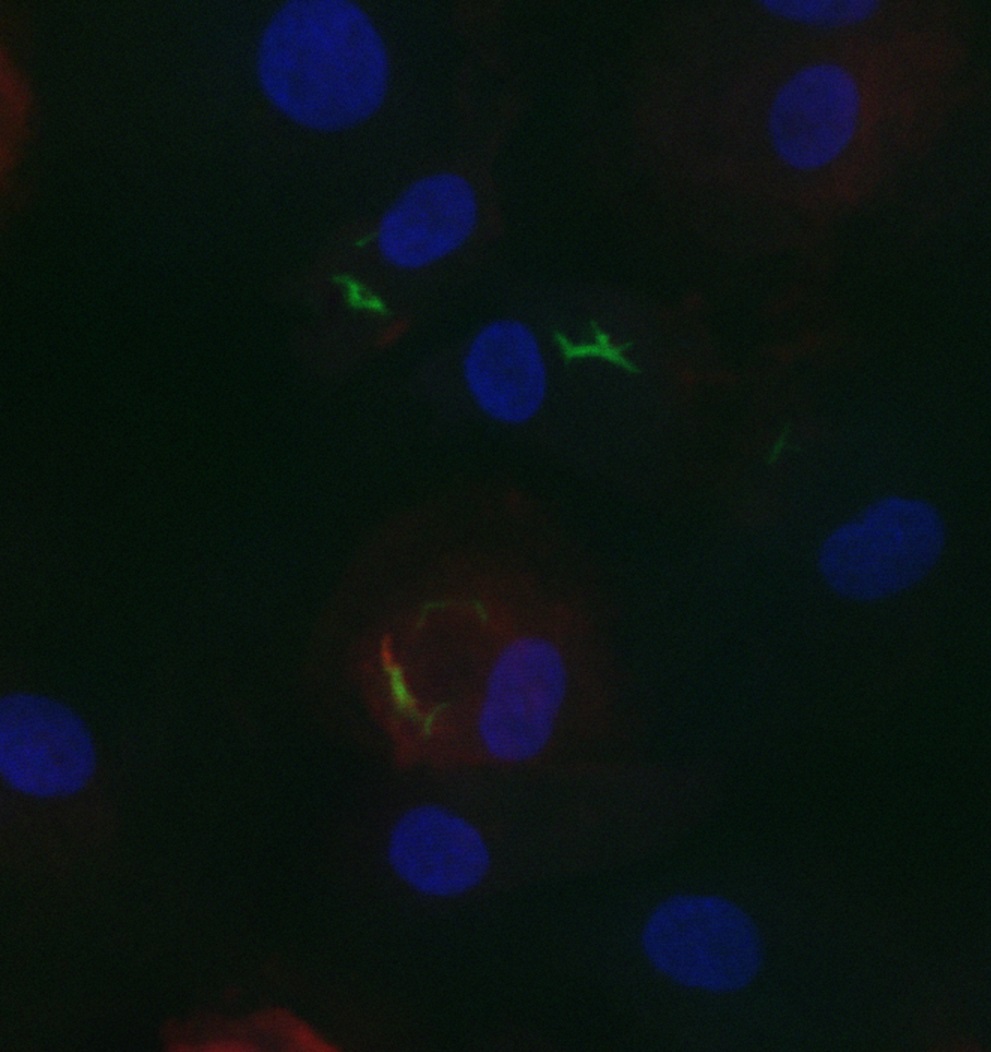

Supplement: Figure 2—source data 1. [file elife-84070-fig2-data1.zip › Figure 2 source data 1/Galectin-3/Galectin-3 WT 72h.JPG]

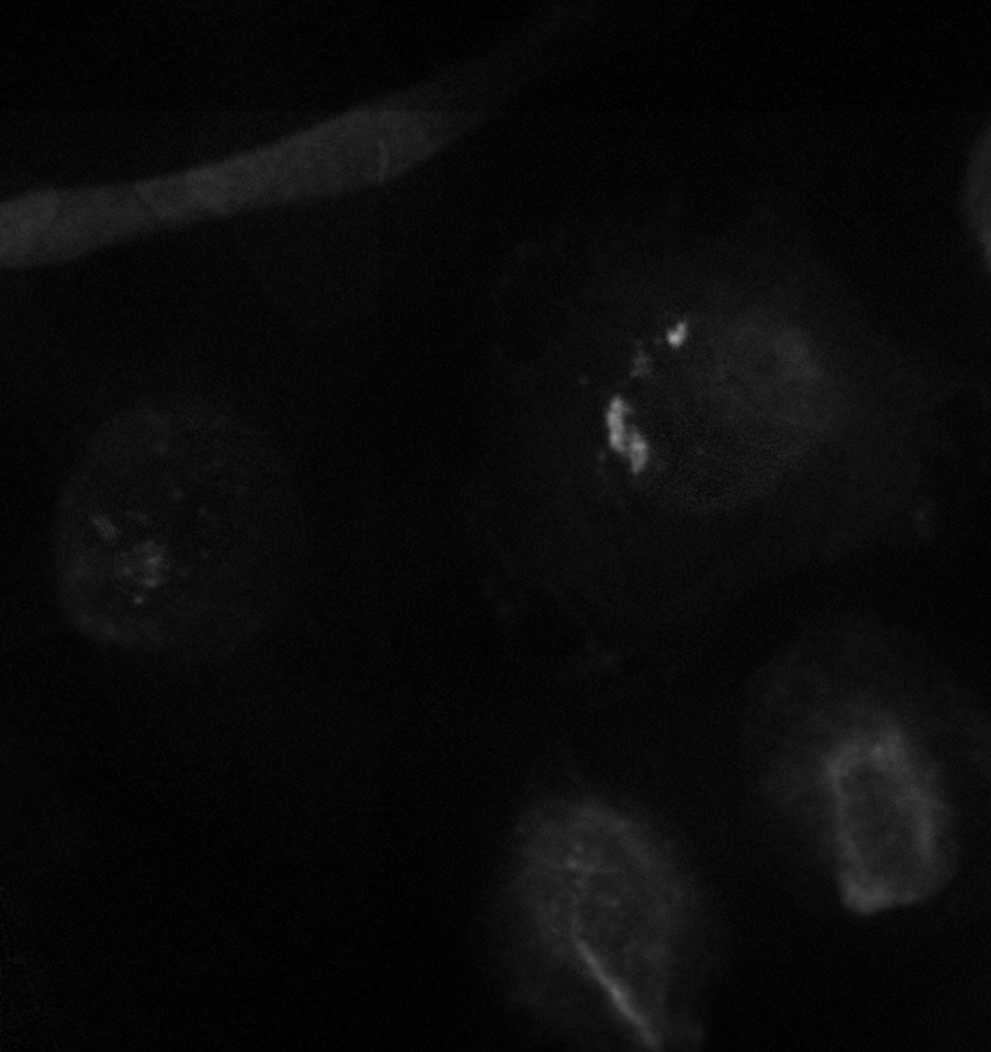

Supplement: Figure 2—source data 1. [file elife-84070-fig2-data1.zip › Figure 2 source data 1/Galectin-3/Galectin-3 WT 120h AF647.tif]

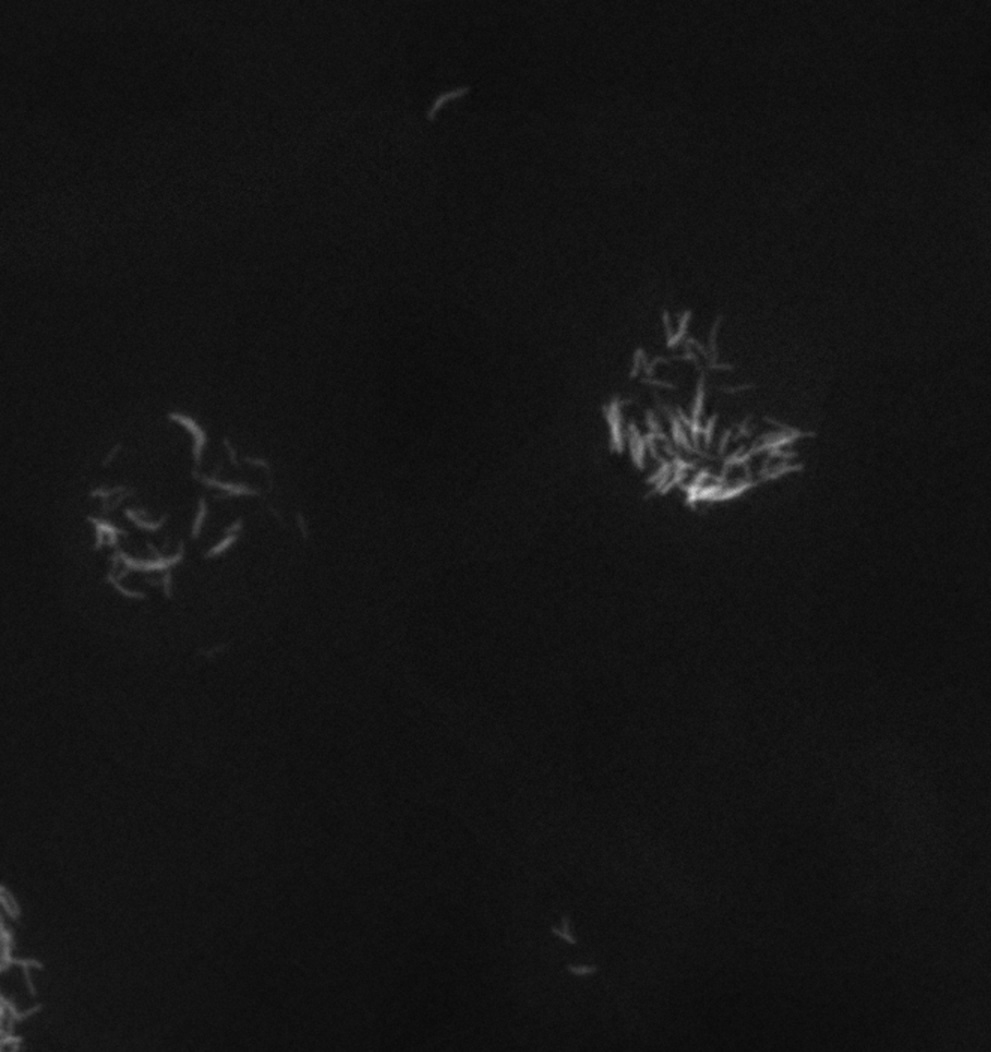

Supplement: Figure 2—source data 1. [file elife-84070-fig2-data1.zip › Figure 2 source data 1/Galectin-3/Galectin-3 WT 120h GFP.tif]

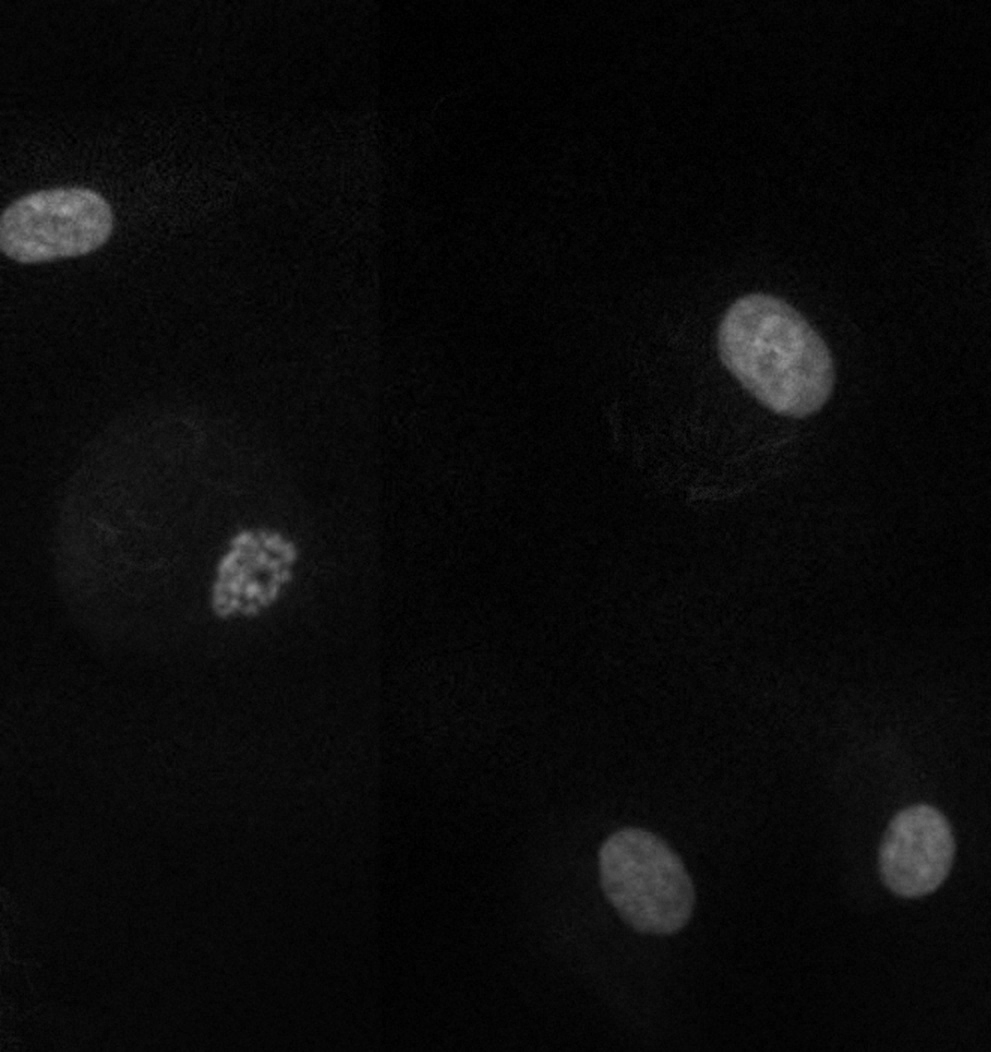

Supplement: Figure 2—source data 1. [file elife-84070-fig2-data1.zip › Figure 2 source data 1/Galectin-3/Galectin-3 WT 120h DAPI.tif]

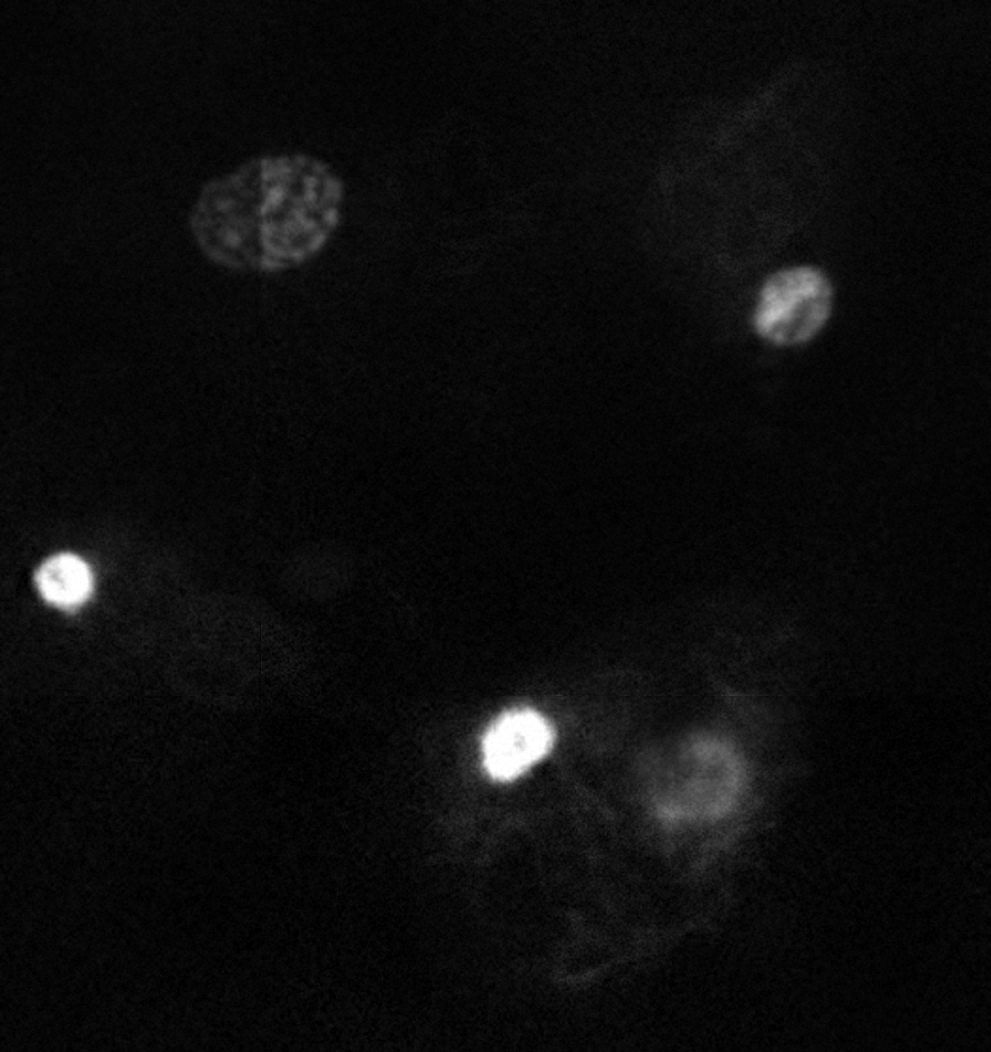

Supplement: Figure 2—source data 1. [file elife-84070-fig2-data1.zip › Figure 2 source data 1/Galectin-3/Galectin-3 WT 120h 2 DAPI.tif]

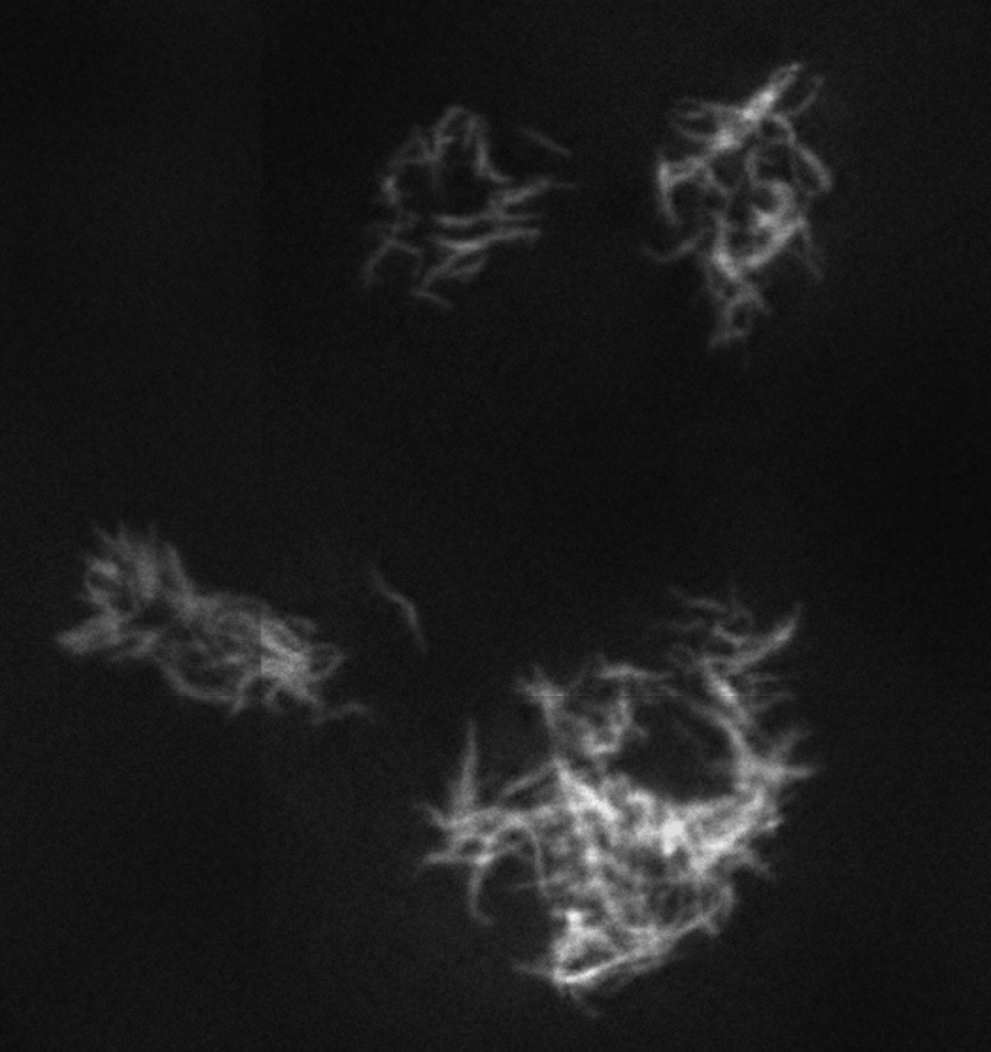

Supplement: Figure 2—source data 1. [file elife-84070-fig2-data1.zip › Figure 2 source data 1/Galectin-3/Galectin-3 WT 120h 2 GFP.tif]

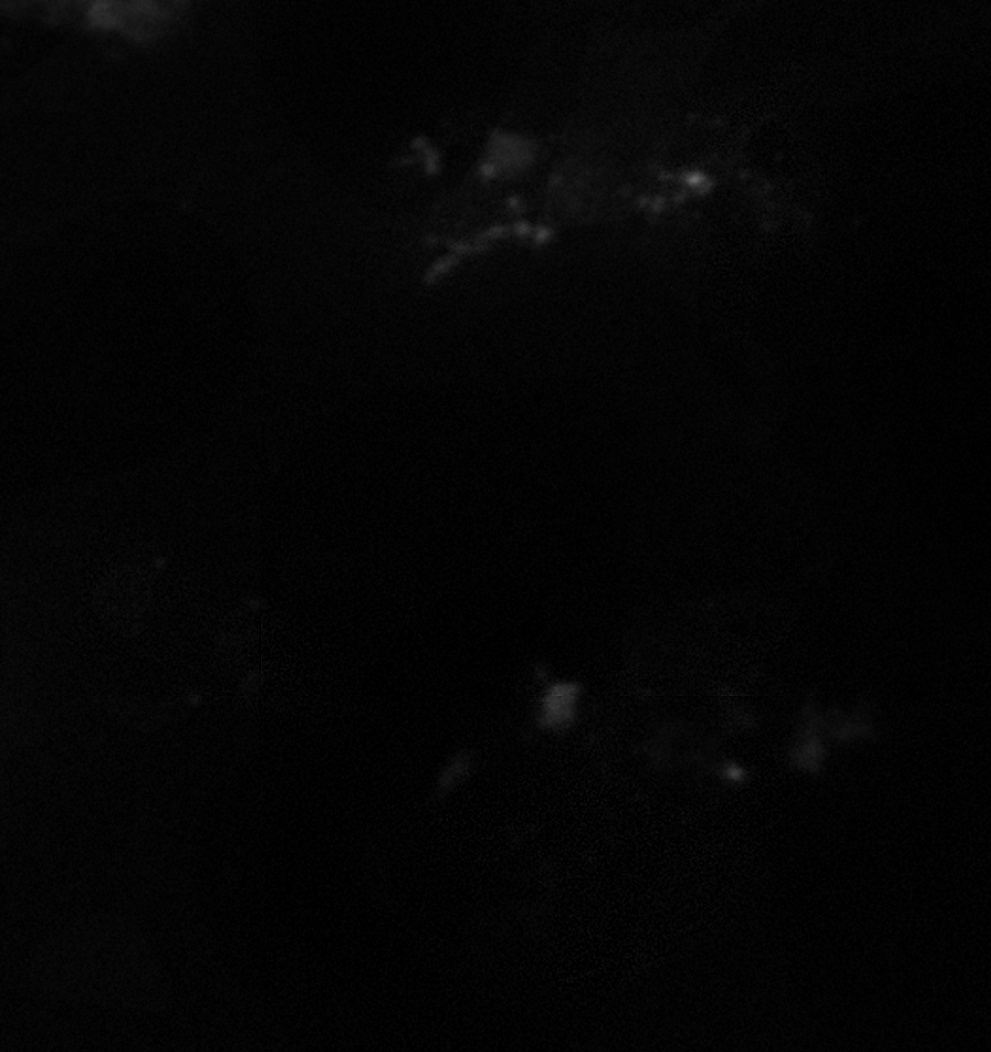

Supplement: Figure 2—source data 1. [file elife-84070-fig2-data1.zip › Figure 2 source data 1/Galectin-3/Galectin-3 WT 120h 2 AF647.tif]

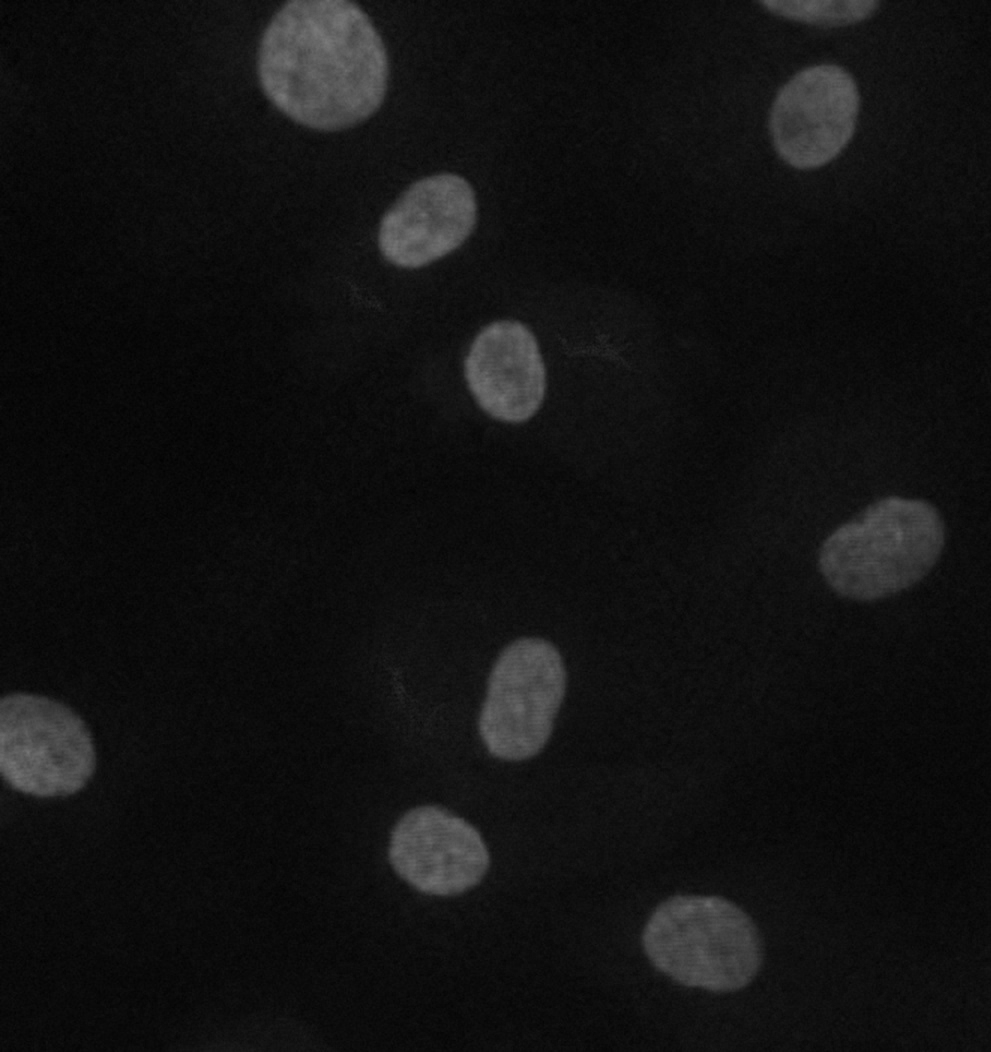

Supplement: Figure 2—source data 1. [file elife-84070-fig2-data1.zip › Figure 2 source data 1/Galectin-3/Galectin-3 WT 72h DAPI.tif]

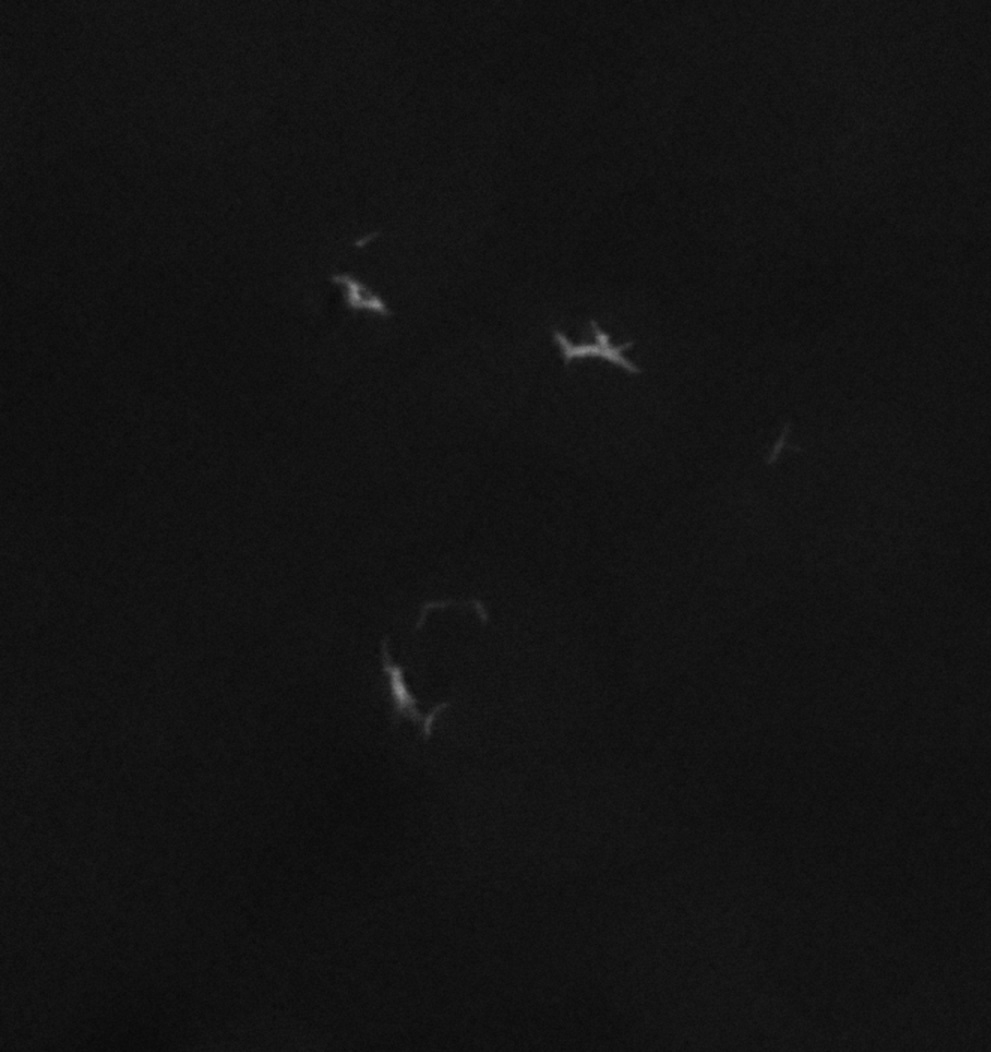

Supplement: Figure 2—source data 1. [file elife-84070-fig2-data1.zip › Figure 2 source data 1/Galectin-3/Galectin-3 WT 72h GFP.tif]

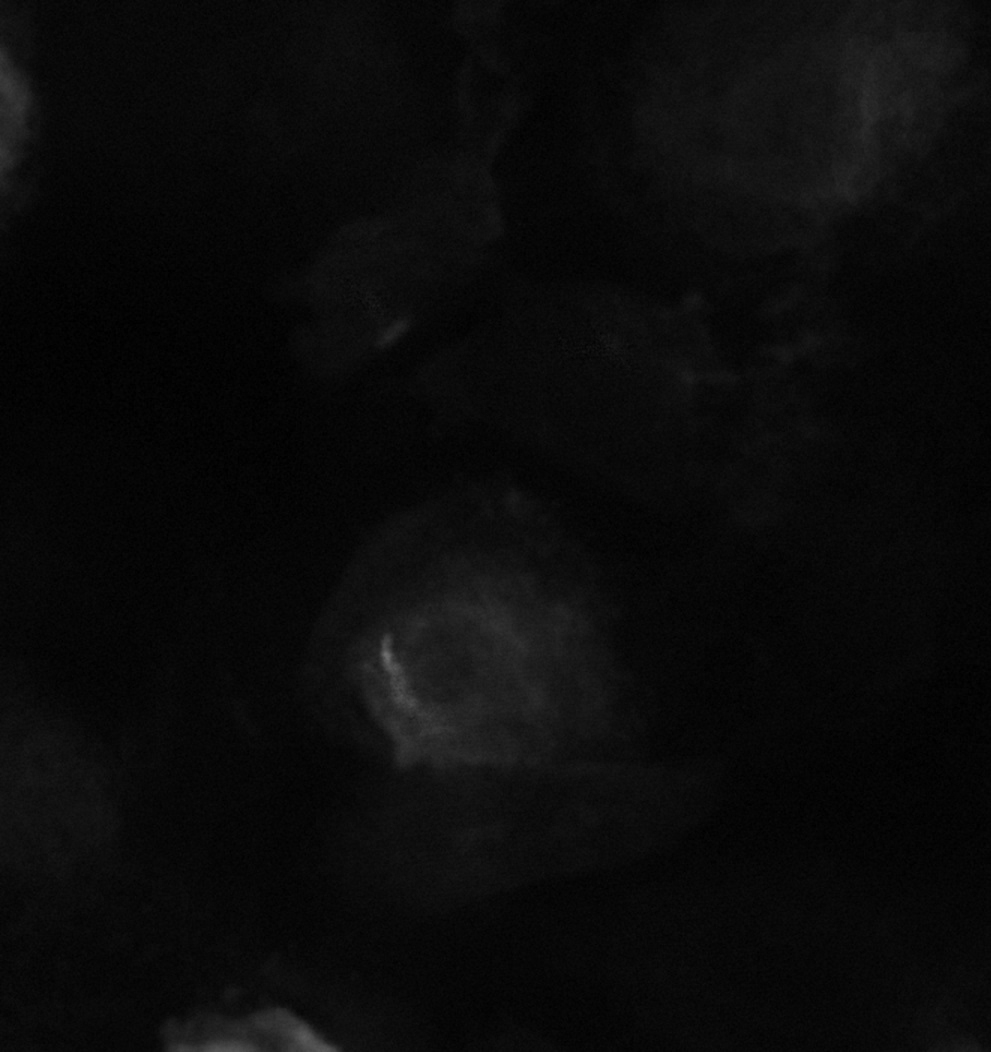

Supplement: Figure 2—source data 1. [file elife-84070-fig2-data1.zip › Figure 2 source data 1/Galectin-3/Galectin-3 WT 72h AF647.tif]

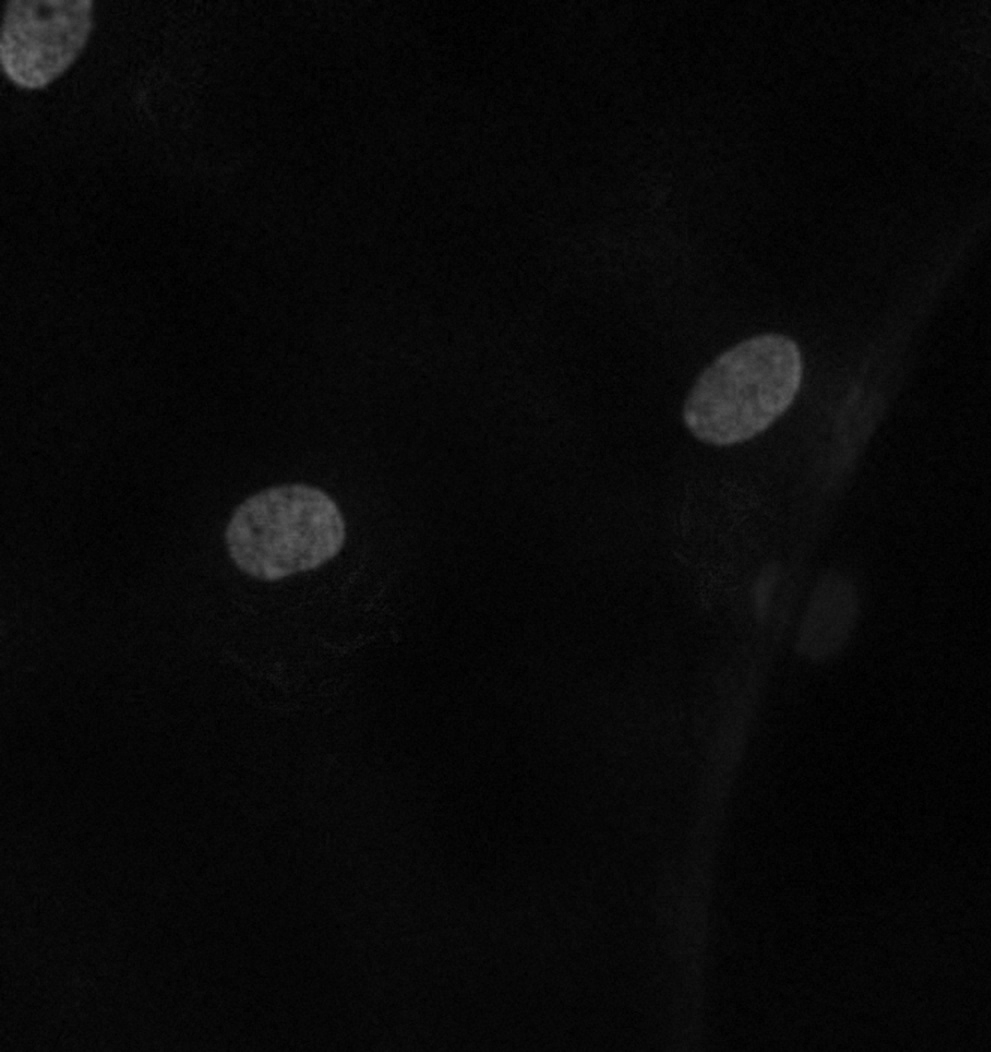

Supplement: Figure 2—source data 1. [file elife-84070-fig2-data1.zip › Figure 2 source data 1/Galectin-3/Galectin-3 WT 72h 2 DAPI.tif]

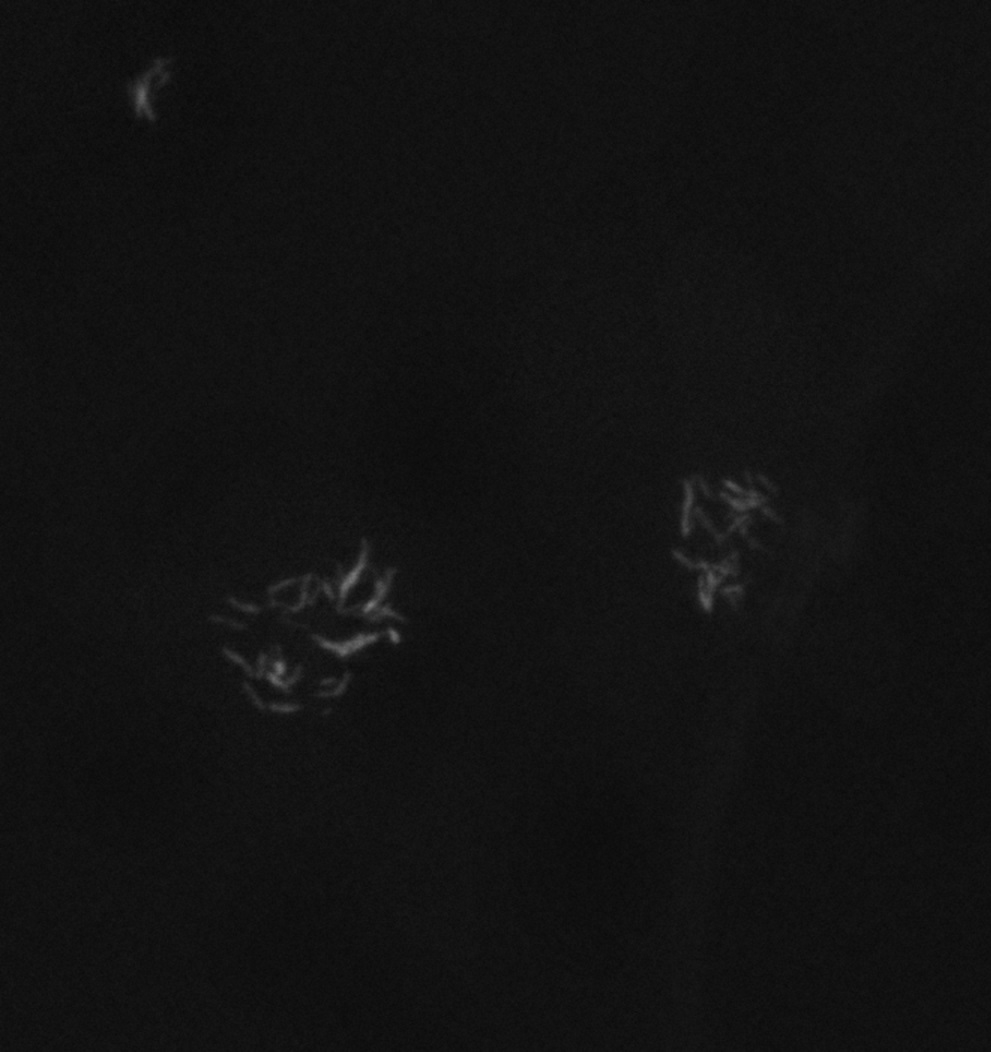

Supplement: Figure 2—source data 1. [file elife-84070-fig2-data1.zip › Figure 2 source data 1/Galectin-3/Galectin-3 WT 72h 2 GFP.tif]

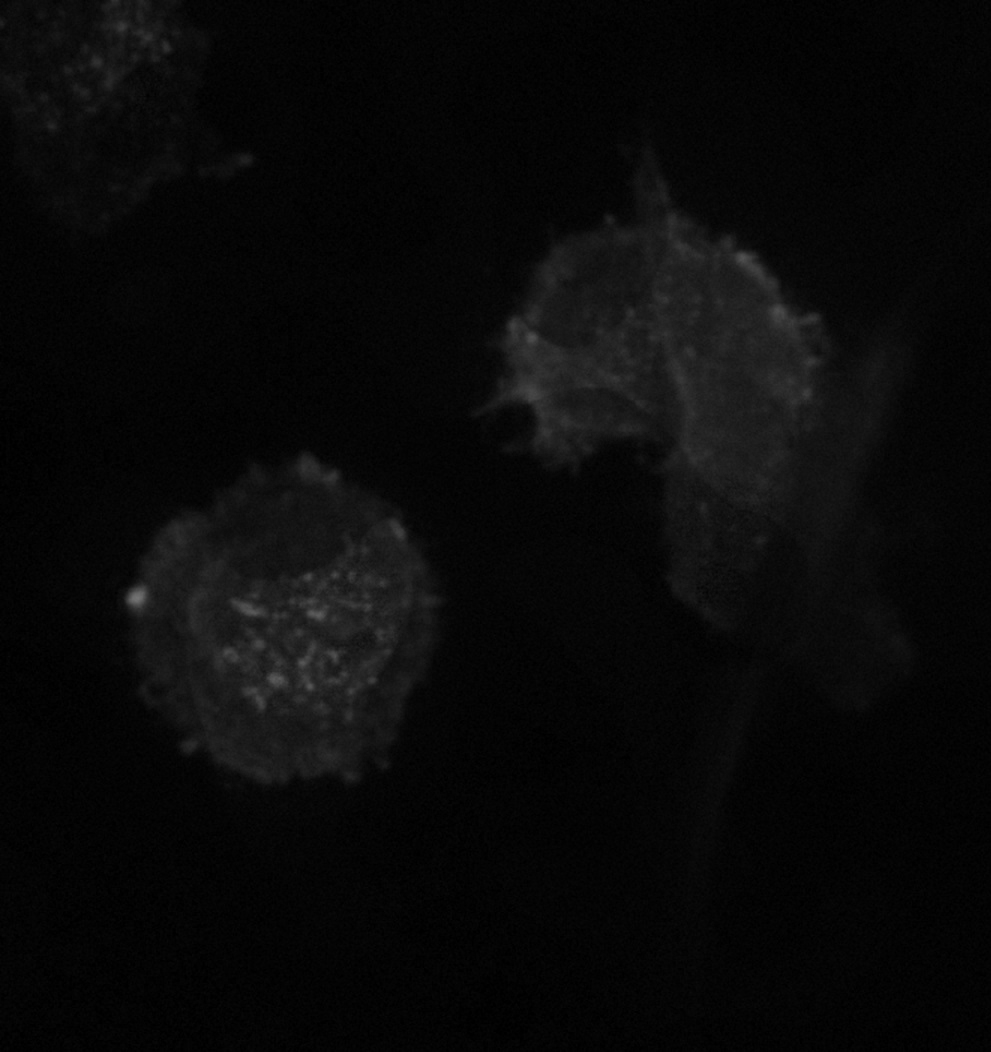

Supplement: Figure 2—source data 1. [file elife-84070-fig2-data1.zip › Figure 2 source data 1/Galectin-3/Galectin-3 WT 72h 2 AF647.tif]

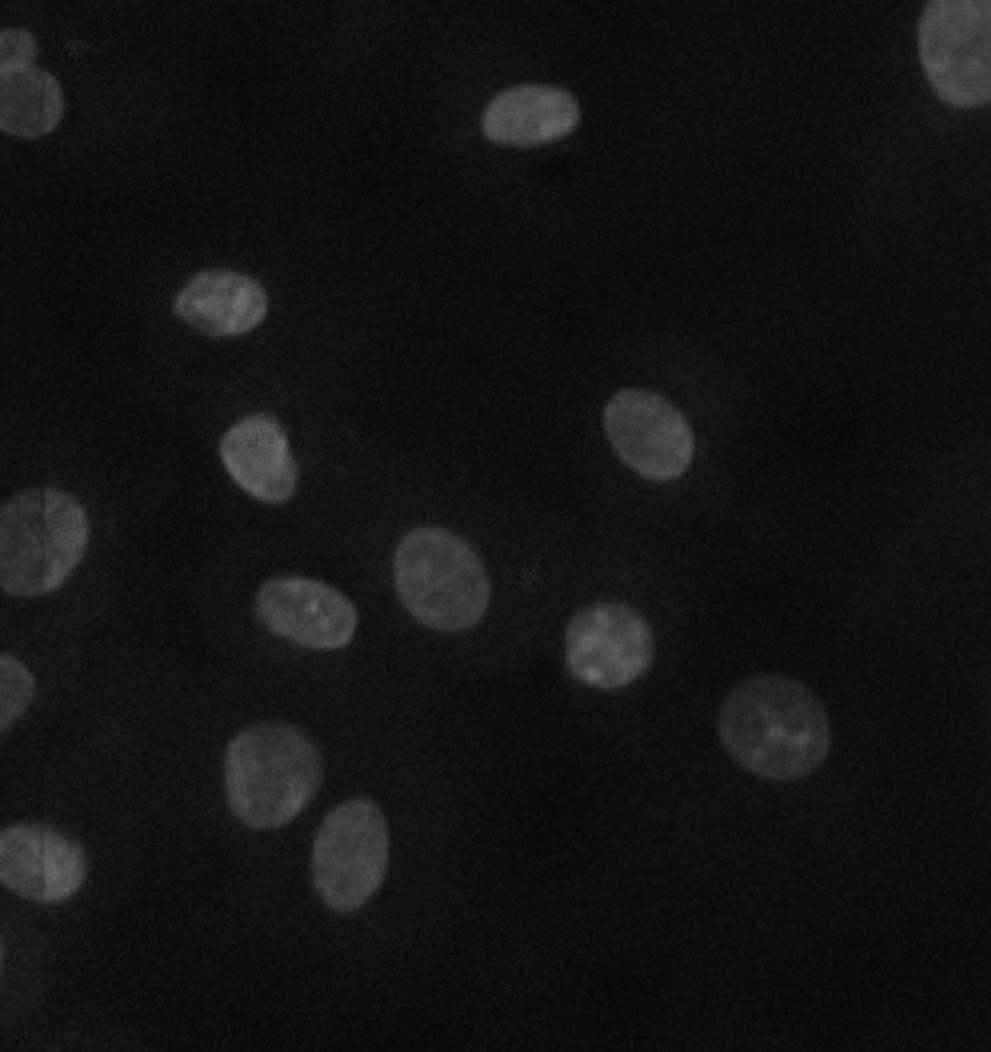

Supplement: Figure 2—source data 1. [file elife-84070-fig2-data1.zip › Figure 2 source data 1/Galectin-3/Galectin-3 WT 24h DAPI.tif]

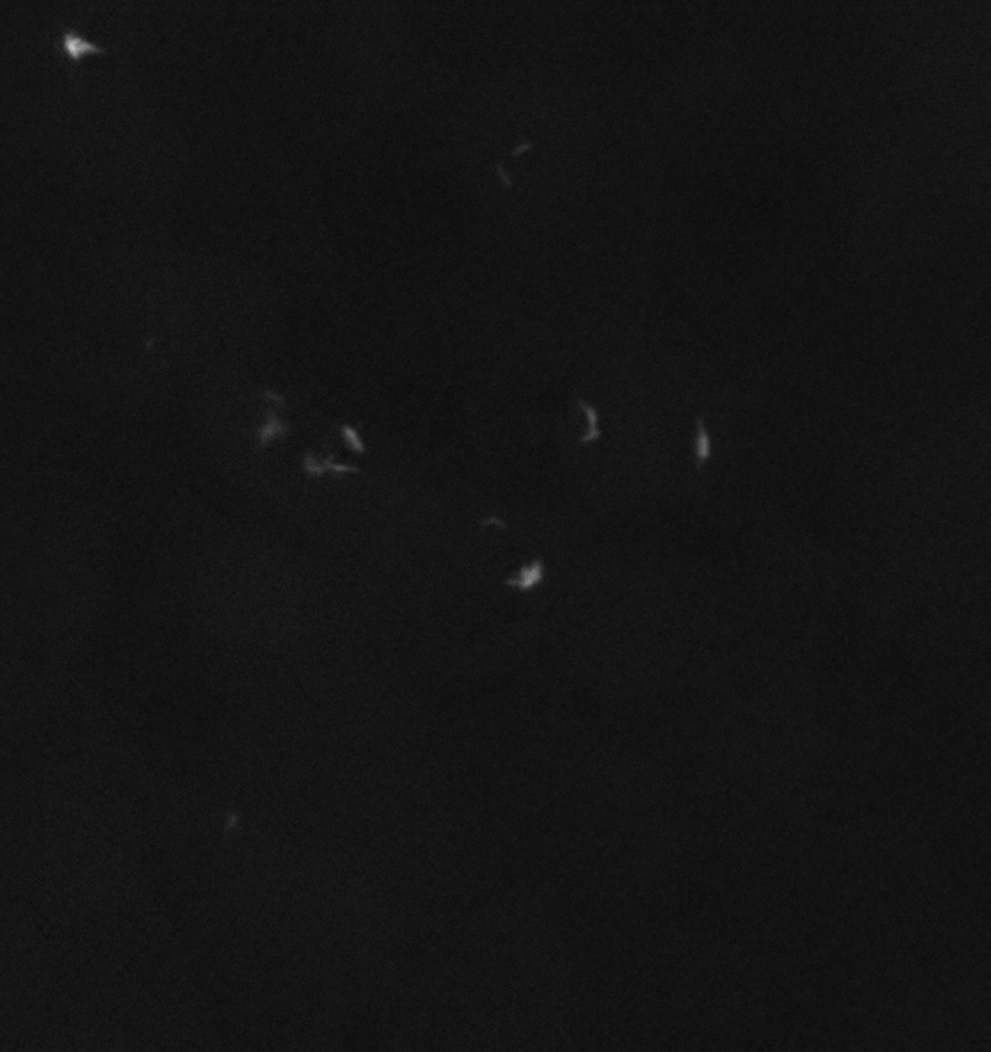

Supplement: Figure 2—source data 1. [file elife-84070-fig2-data1.zip › Figure 2 source data 1/Galectin-3/Galectin-3 WT 24h GFP.tif]

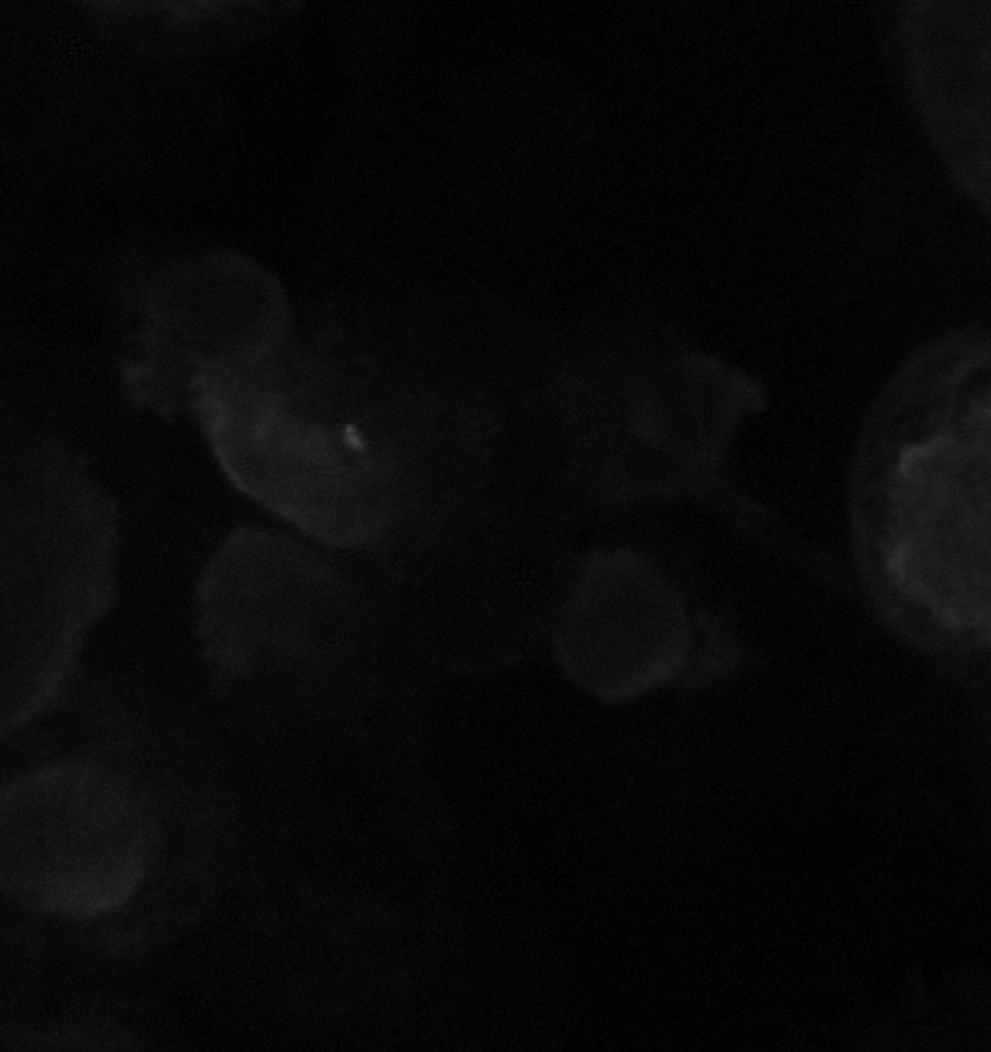

Supplement: Figure 2—source data 1. [file elife-84070-fig2-data1.zip › Figure 2 source data 1/Galectin-3/Galectin-3 WT 24h AF647.tif]

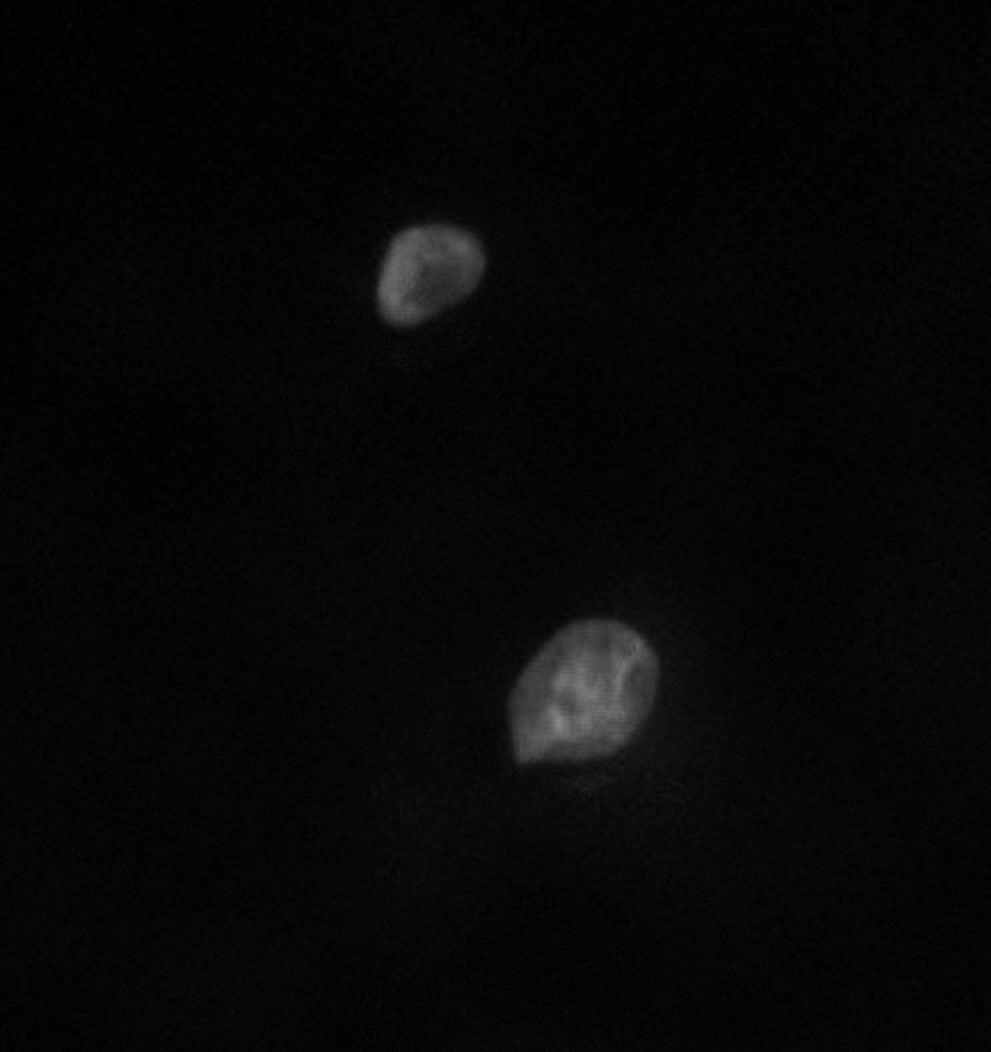

Supplement: Figure 2—source data 1. [file elife-84070-fig2-data1.zip › Figure 2 source data 1/Galectin-3/Galectin-3 WT 24h 2 DAPI.tif]

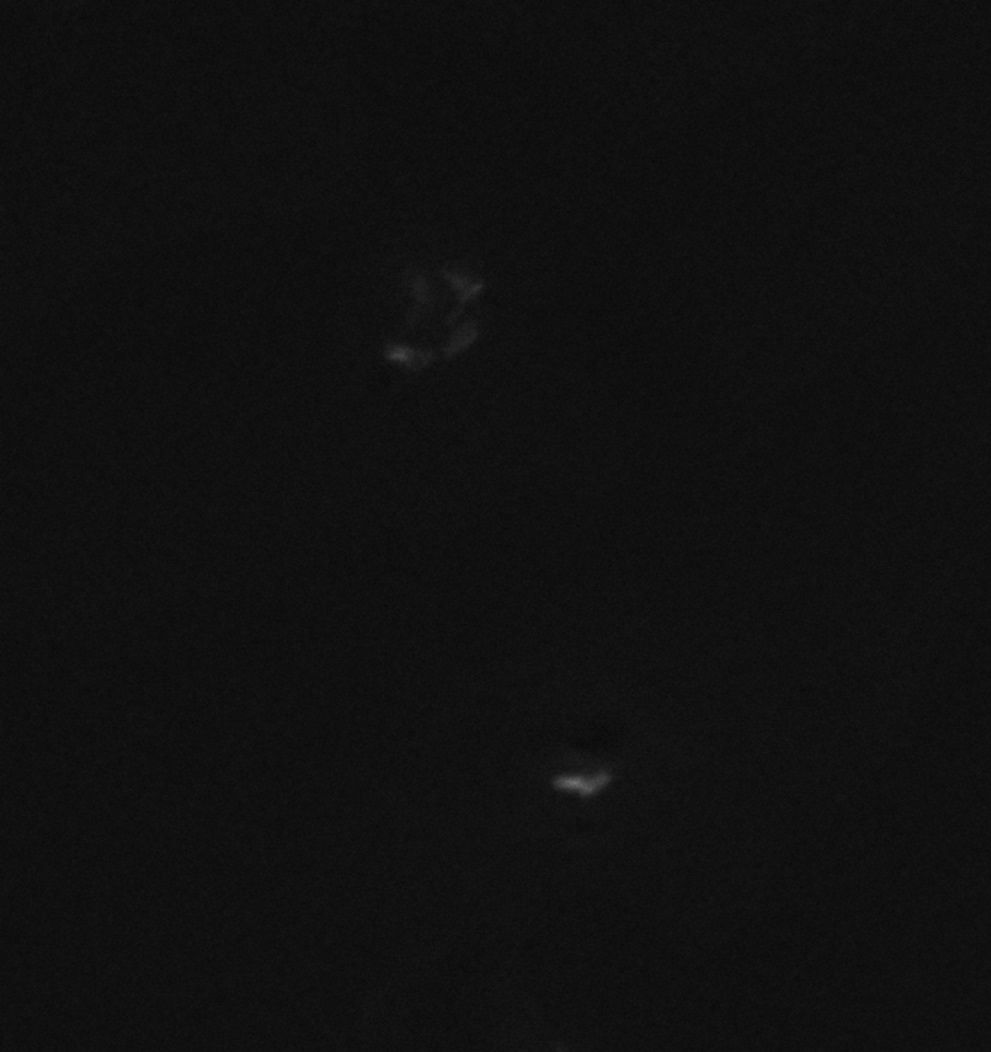

Supplement: Figure 2—source data 1. [file elife-84070-fig2-data1.zip › Figure 2 source data 1/Galectin-3/Galectin-3 WT 24h 2 GFP.tif]

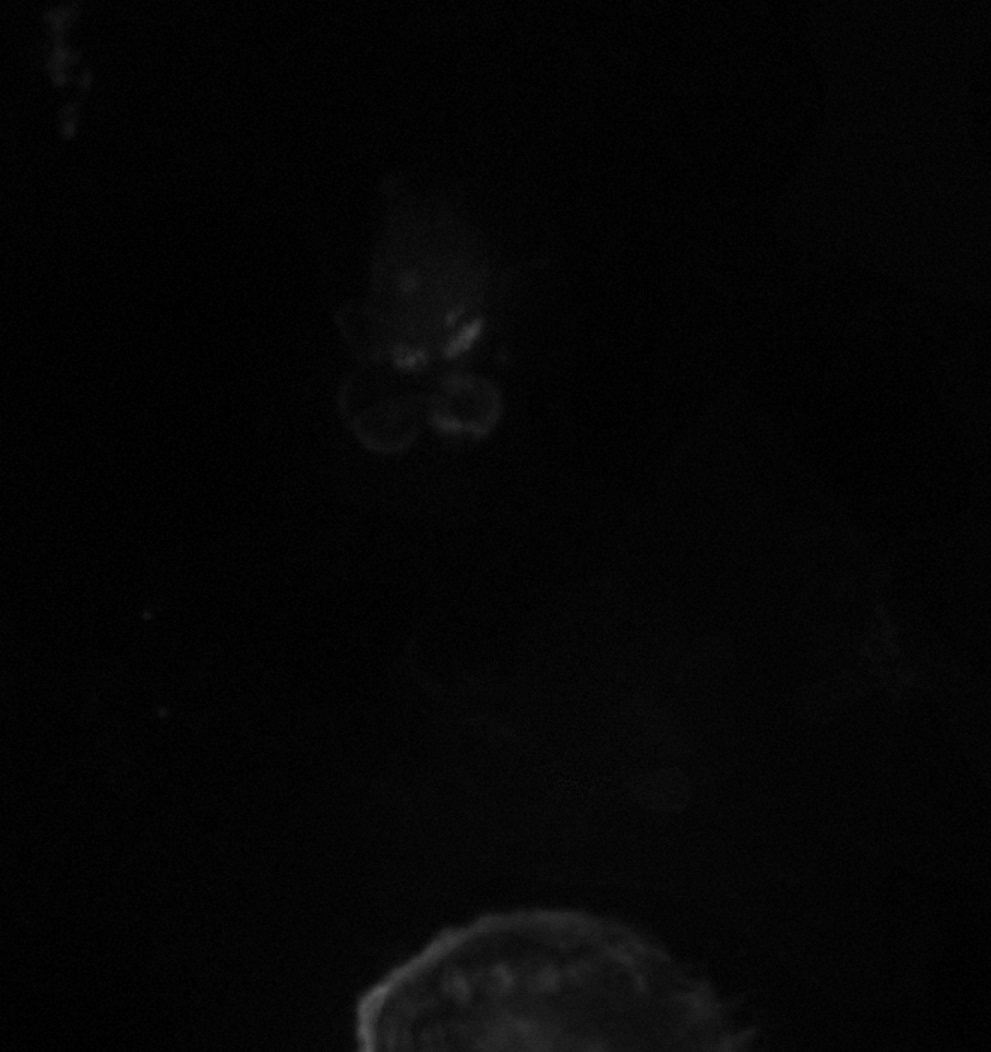

Supplement: Figure 2—source data 1. [file elife-84070-fig2-data1.zip › Figure 2 source data 1/Galectin-3/Galectin-3 WT 24h 2 AF647.tif]

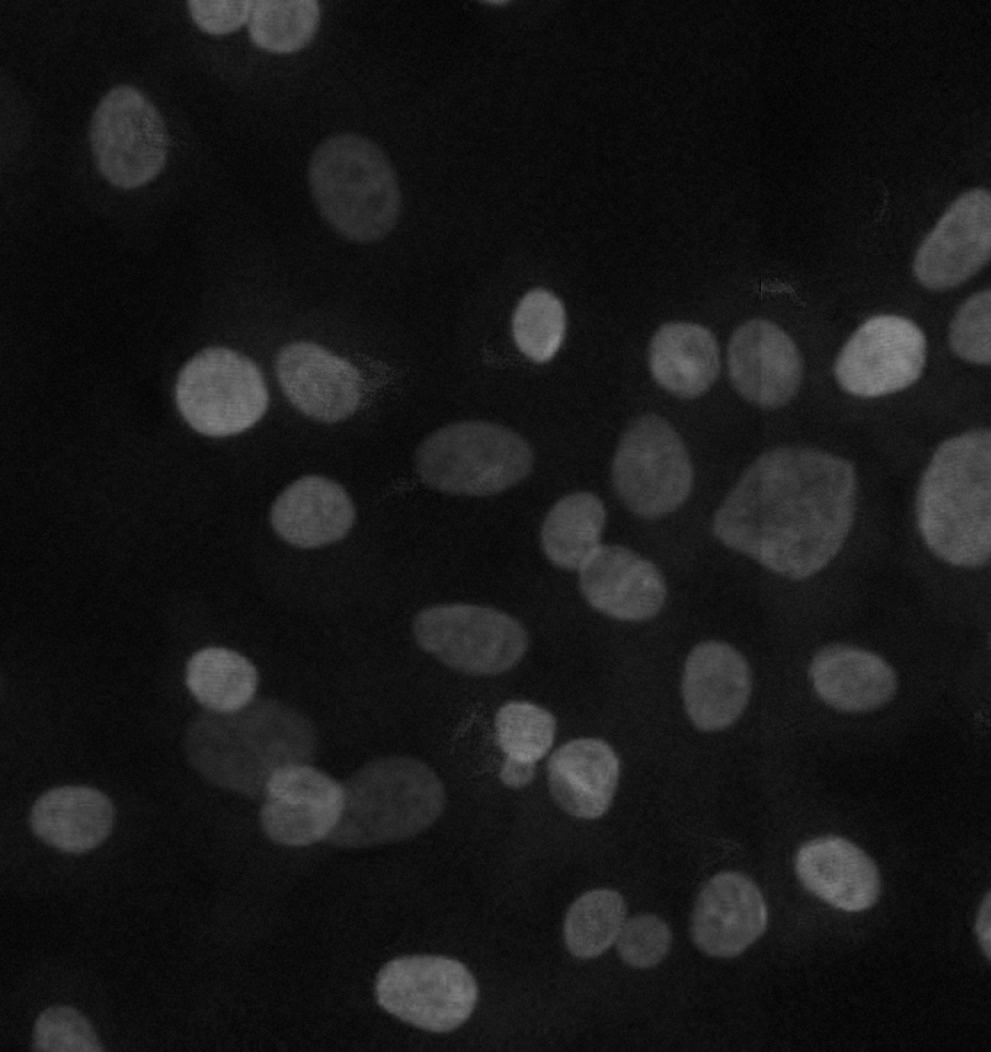

Supplement: Figure 2—source data 1. [file elife-84070-fig2-data1.zip › Figure 2 source data 1/Galectin-3/Galectin-3 ESX 120h DAPI.tif]

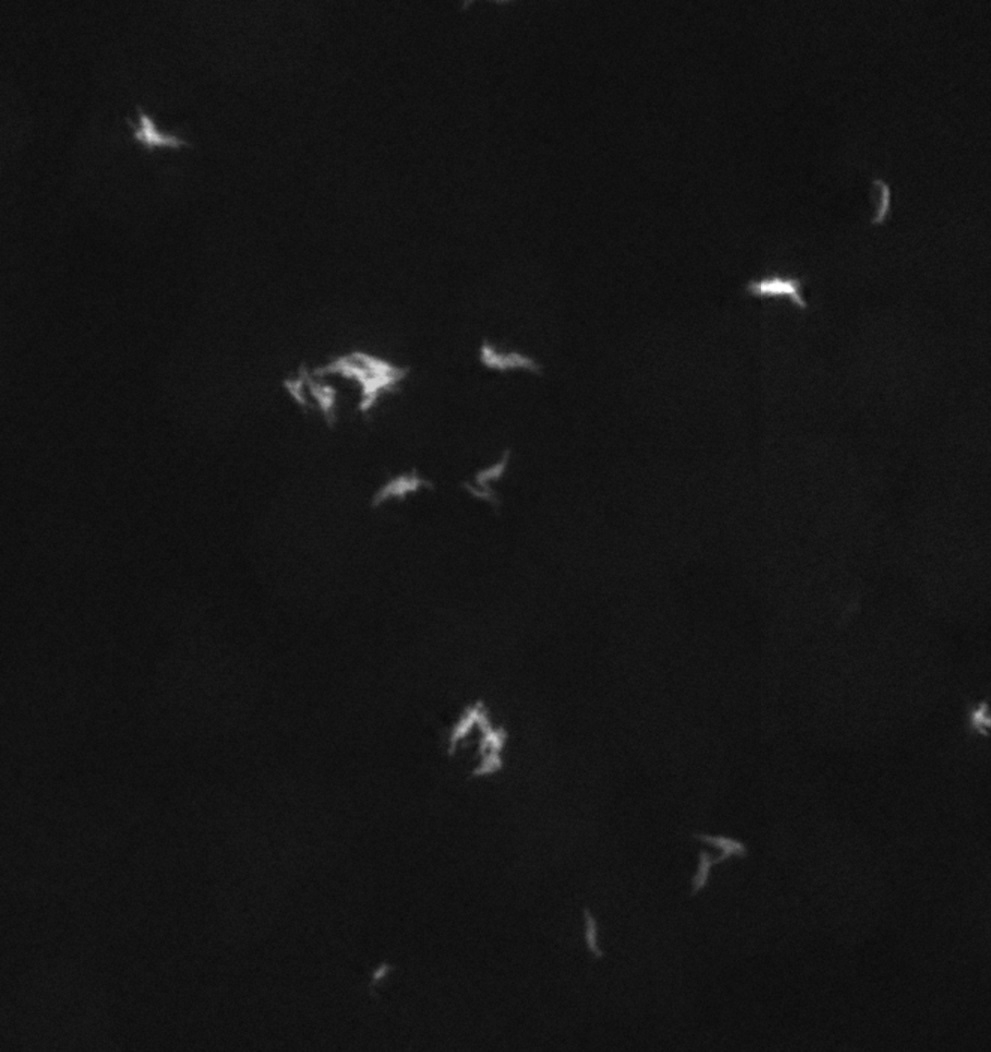

Supplement: Figure 2—source data 1. [file elife-84070-fig2-data1.zip › Figure 2 source data 1/Galectin-3/Galectin-3 ESX 120h GFP.tif]

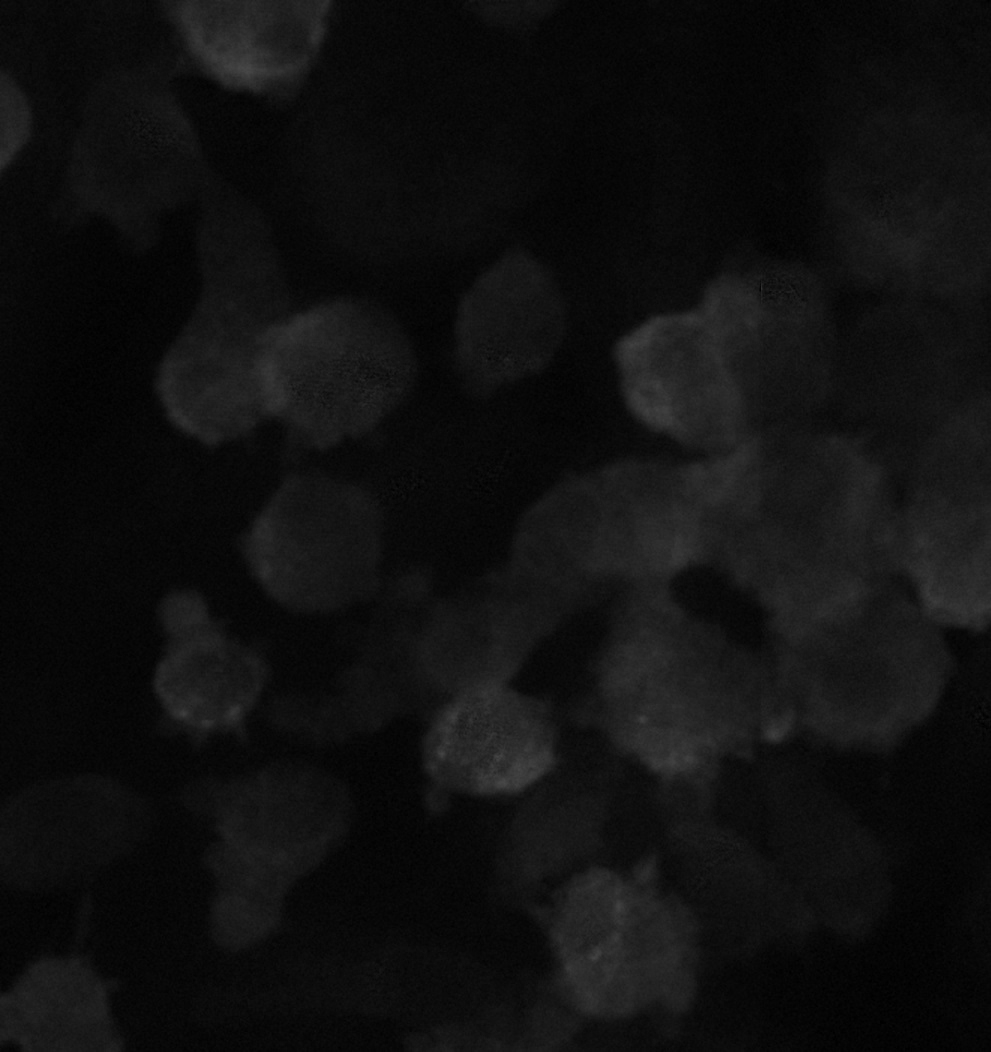

Supplement: Figure 2—source data 1. [file elife-84070-fig2-data1.zip › Figure 2 source data 1/Galectin-3/Galectin-3 ESX 120h AF647.tif]

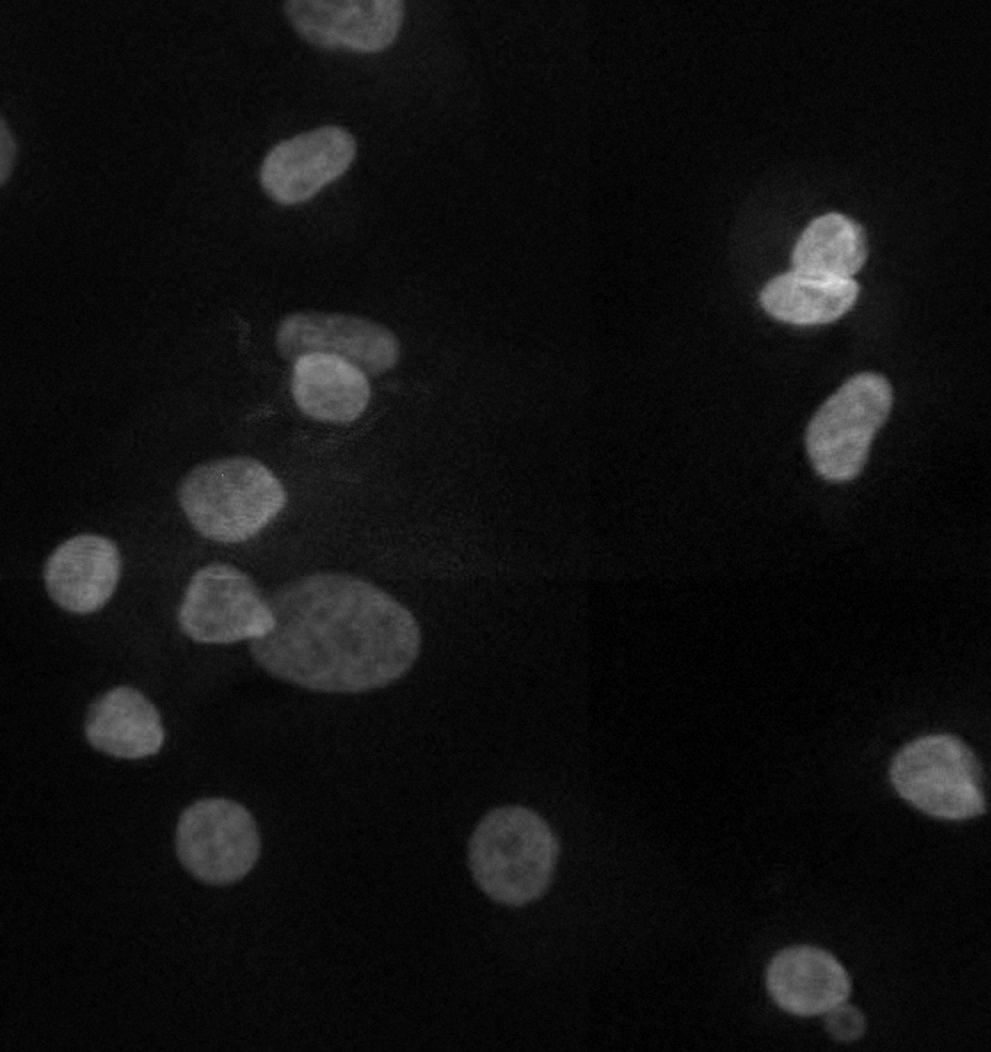

Supplement: Figure 2—source data 1. [file elife-84070-fig2-data1.zip › Figure 2 source data 1/Galectin-3/Galectin-3 ESX 72h DAPI.tif]

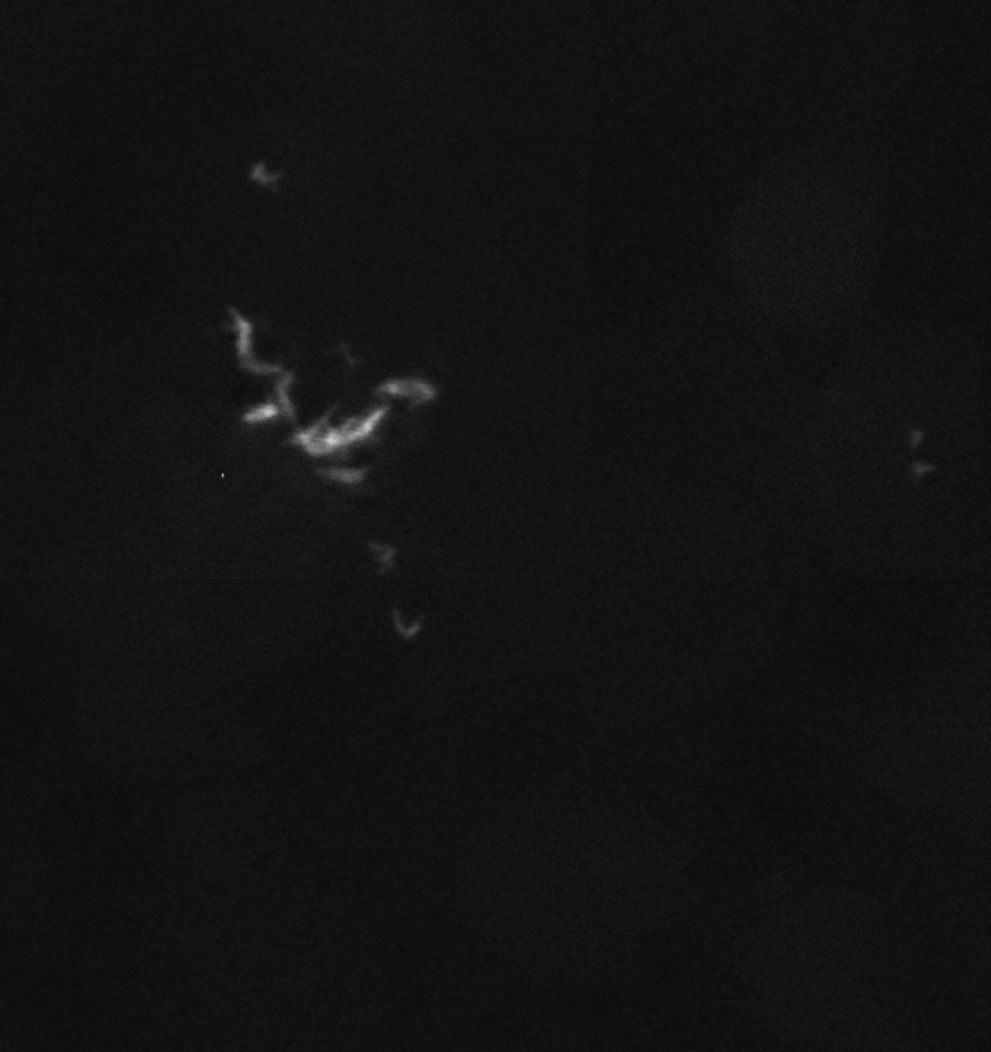

Supplement: Figure 2—source data 1. [file elife-84070-fig2-data1.zip › Figure 2 source data 1/Galectin-3/Galectin-3 ESX 72h GFP.tif]

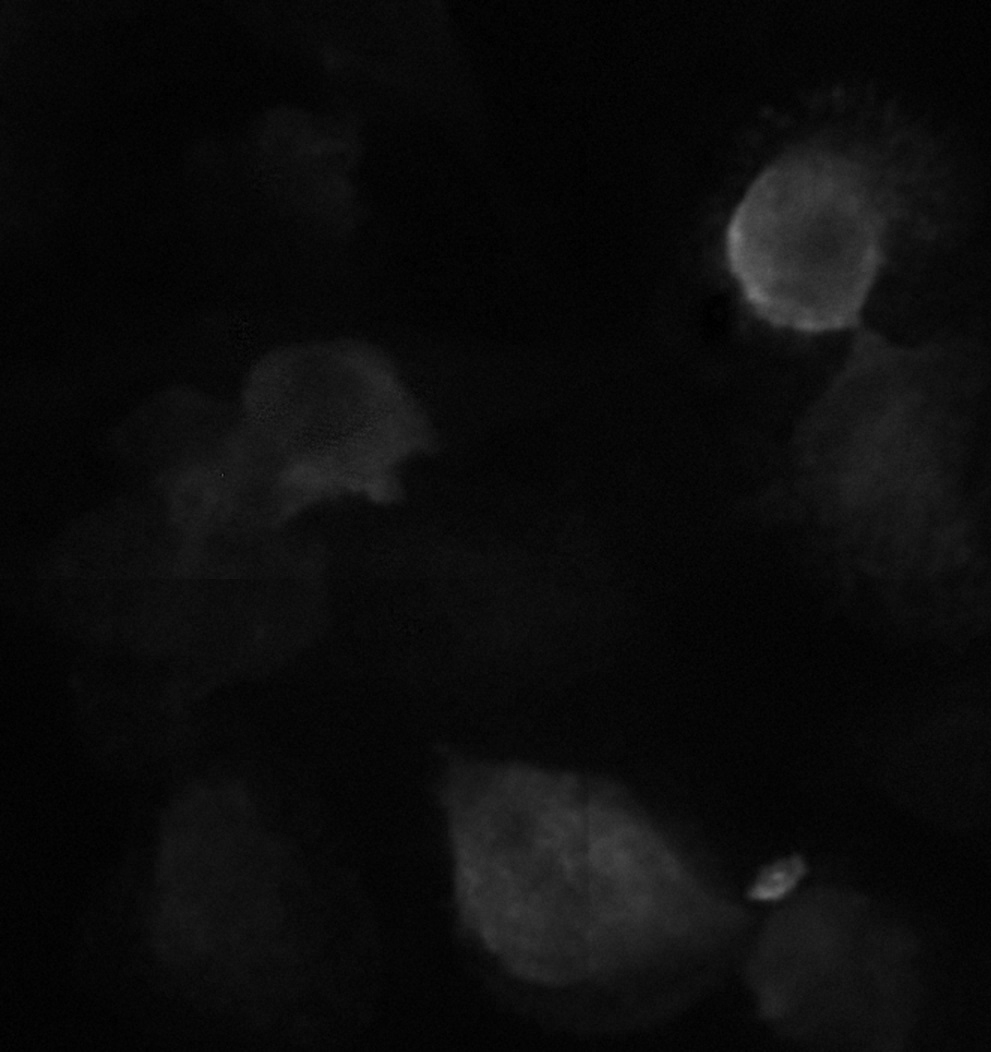

Supplement: Figure 2—source data 1. [file elife-84070-fig2-data1.zip › Figure 2 source data 1/Galectin-3/Galectin-3 ESX 72h AF647.tif]

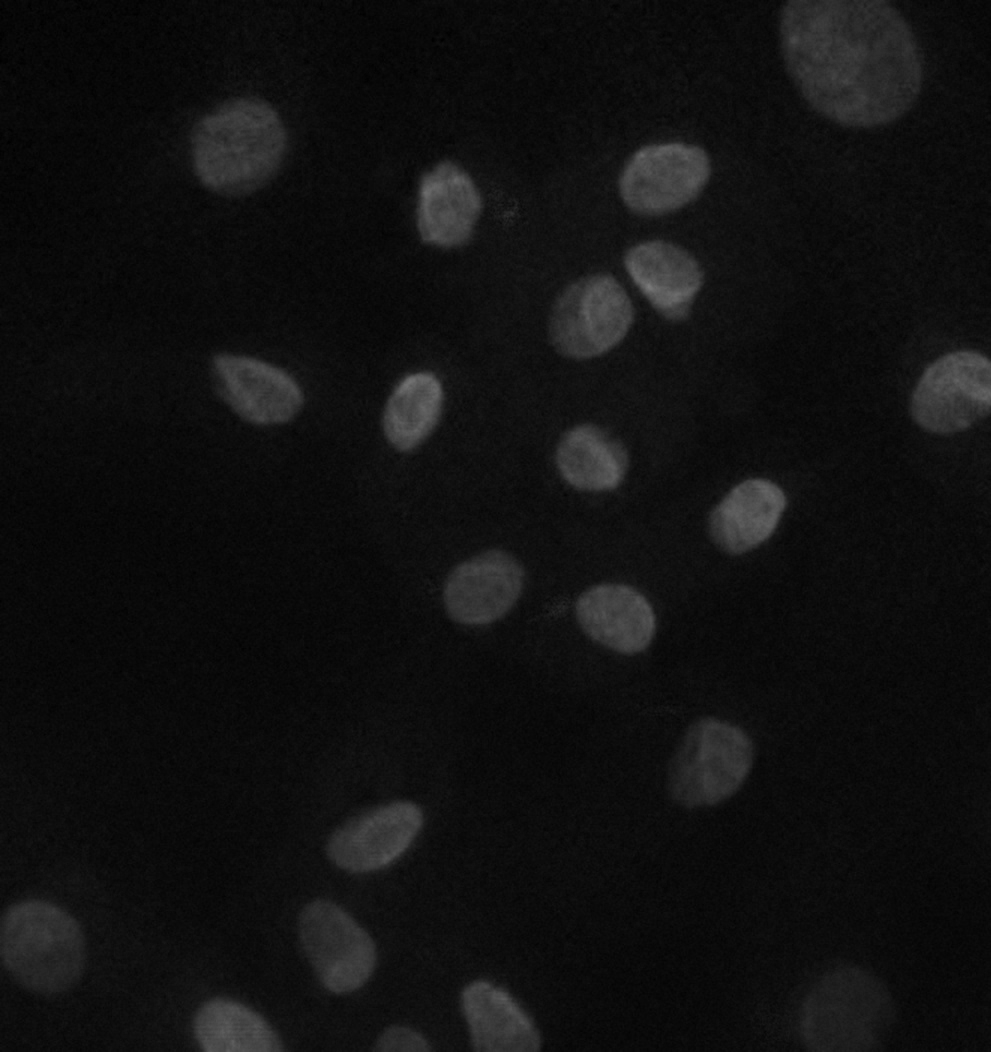

Supplement: Figure 2—source data 1. [file elife-84070-fig2-data1.zip › Figure 2 source data 1/Galectin-3/Galectin-3 ESX 24h DAPI.tif]

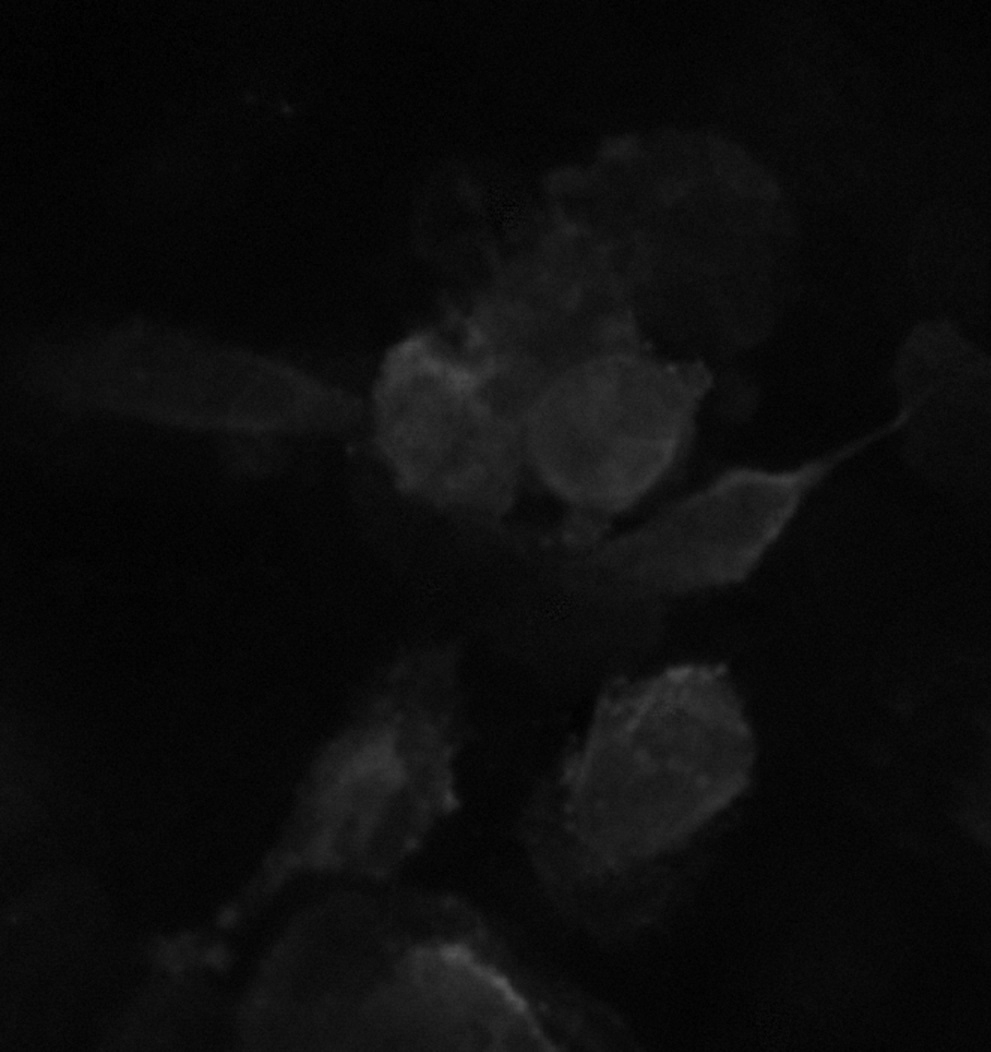

Supplement: Figure 2—source data 1. [file elife-84070-fig2-data1.zip › Figure 2 source data 1/Galectin-3/Galectin-3 ESX 24h AF647.tif]

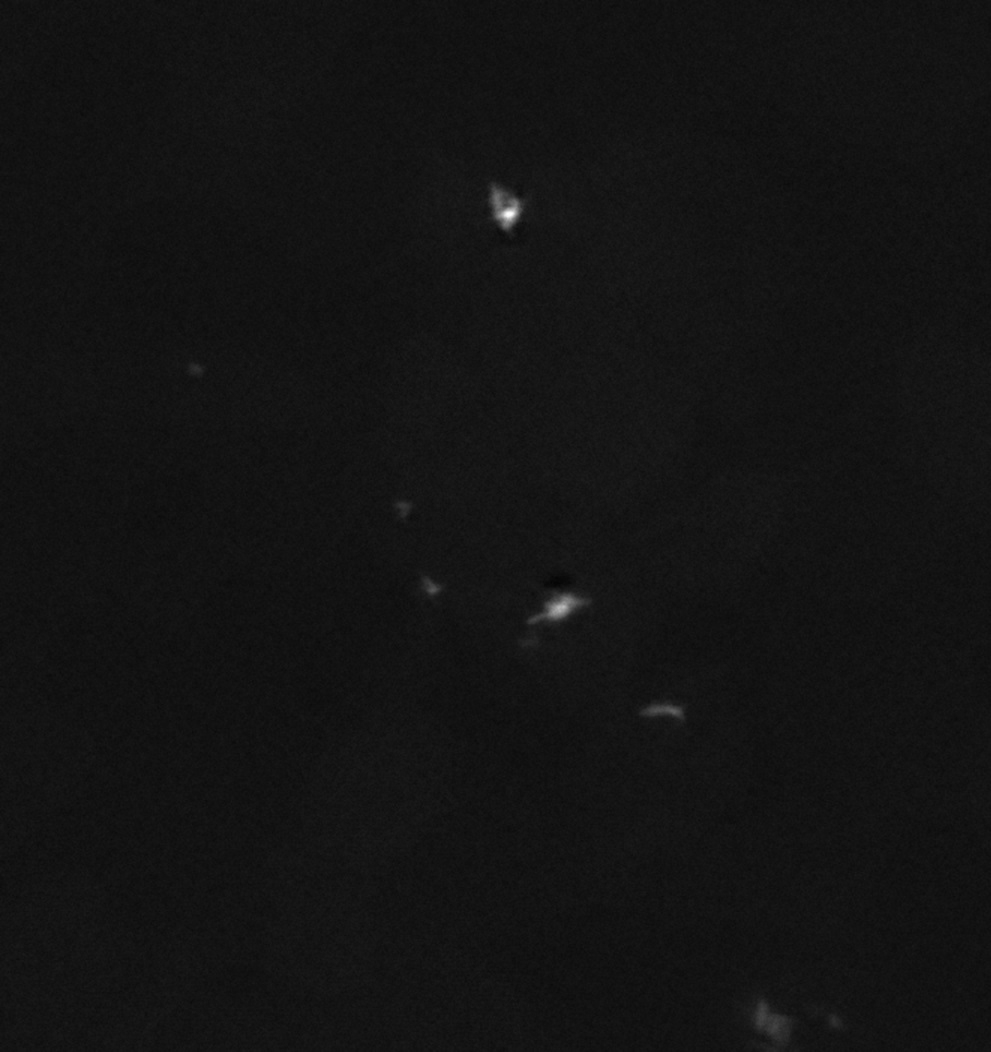

Supplement: Figure 2—source data 1. [file elife-84070-fig2-data1.zip › Figure 2 source data 1/Galectin-3/Galectin-3 ESX 24h GFP.tif]

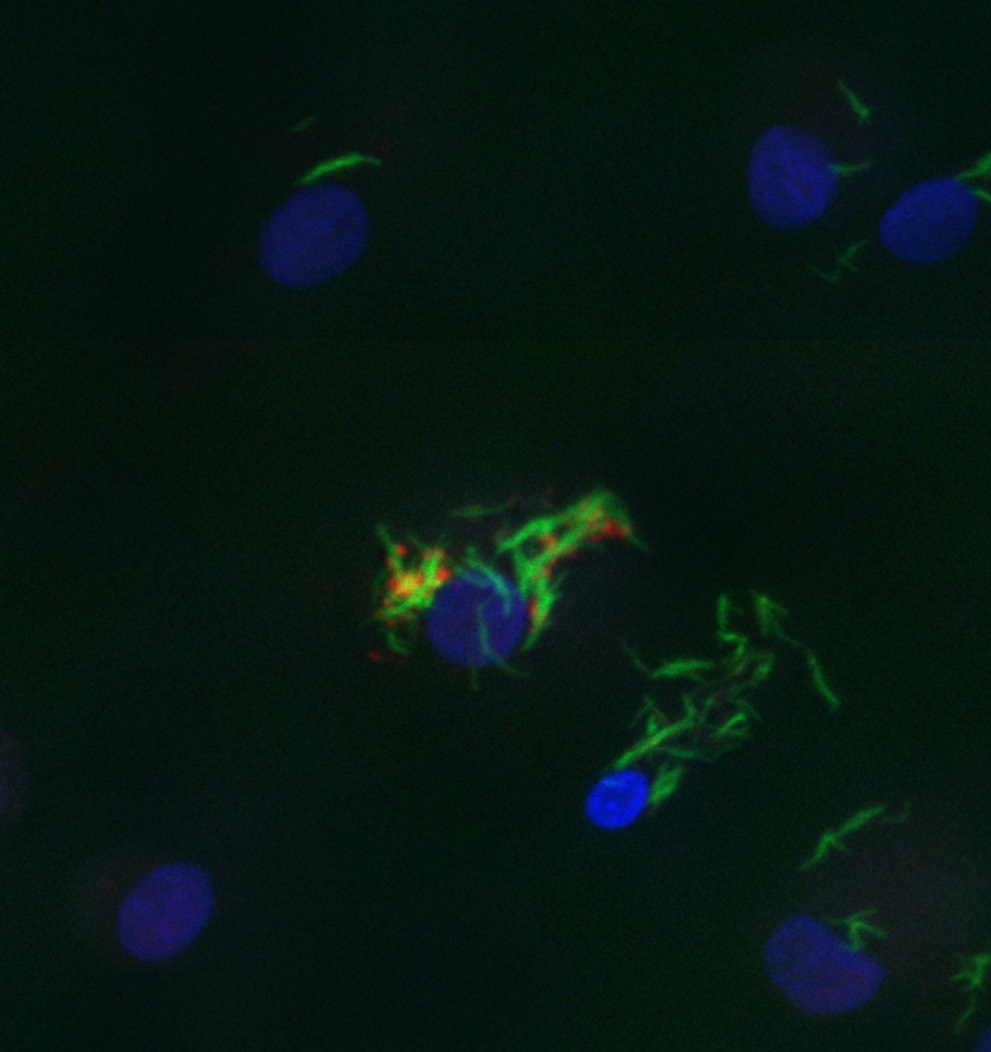

Supplement: Figure 2—source data 1. [file elife-84070-fig2-data1.zip › Figure 2 source data 1/LAMP-1/LAMP-1 120h.JPG]

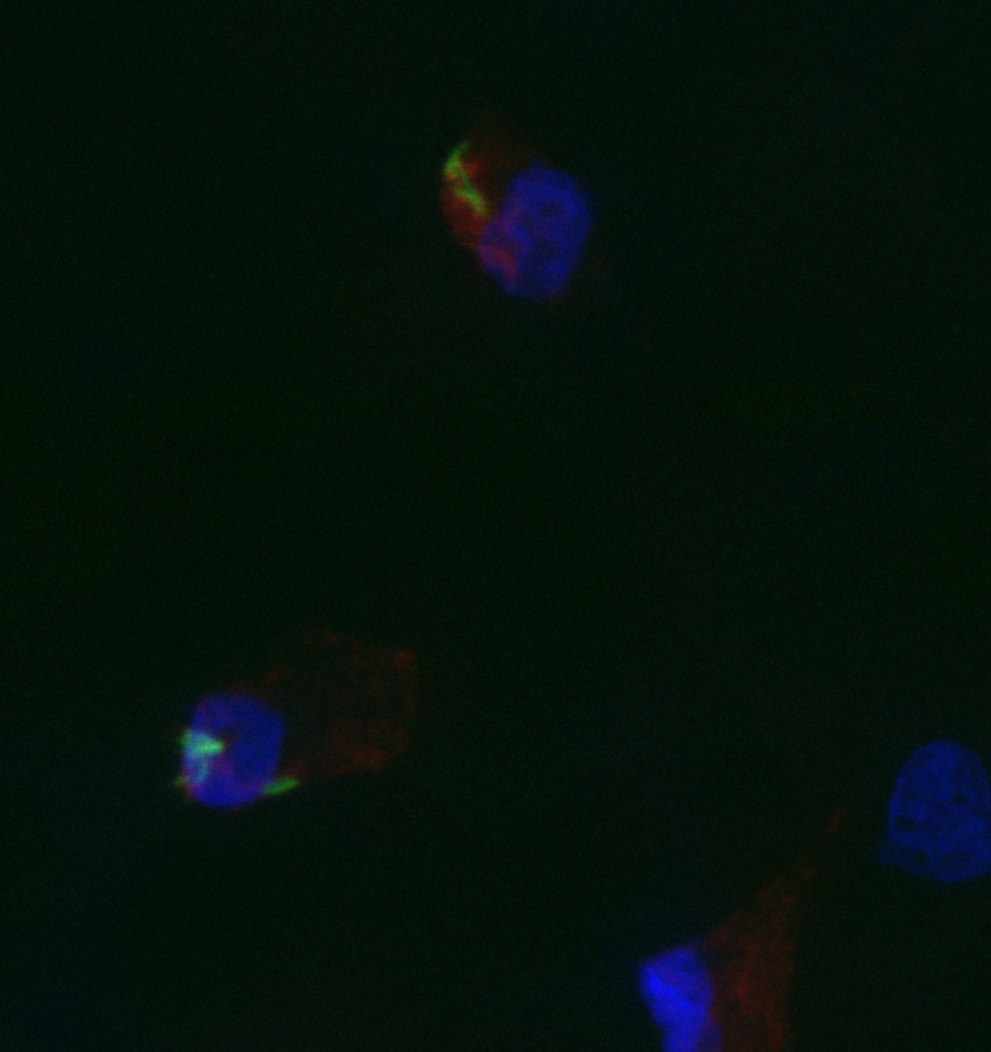

Supplement: Figure 2—source data 1. [file elife-84070-fig2-data1.zip › Figure 2 source data 1/LAMP-1/LAMP-1 24h.JPG]

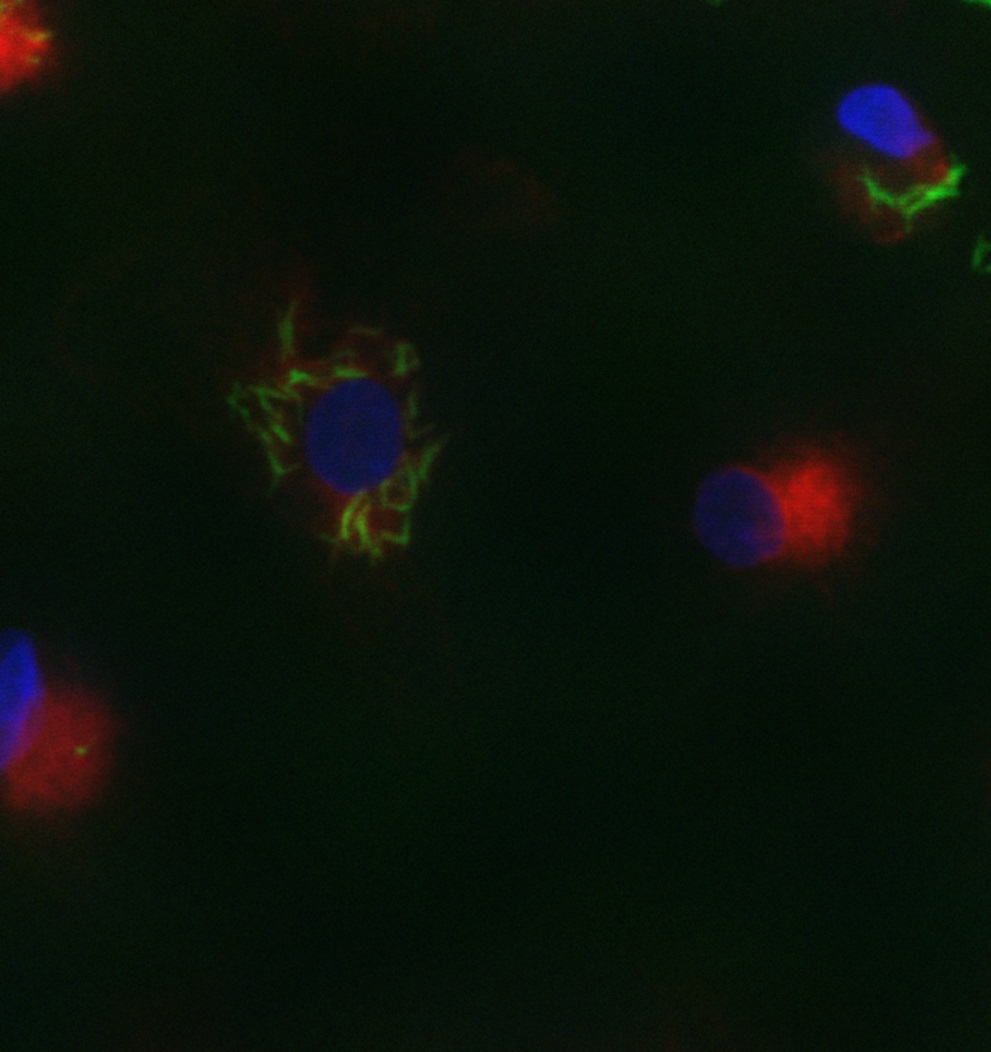

Supplement: Figure 2—source data 1. [file elife-84070-fig2-data1.zip › Figure 2 source data 1/LAMP-1/LAMP-1 72h.JPG]

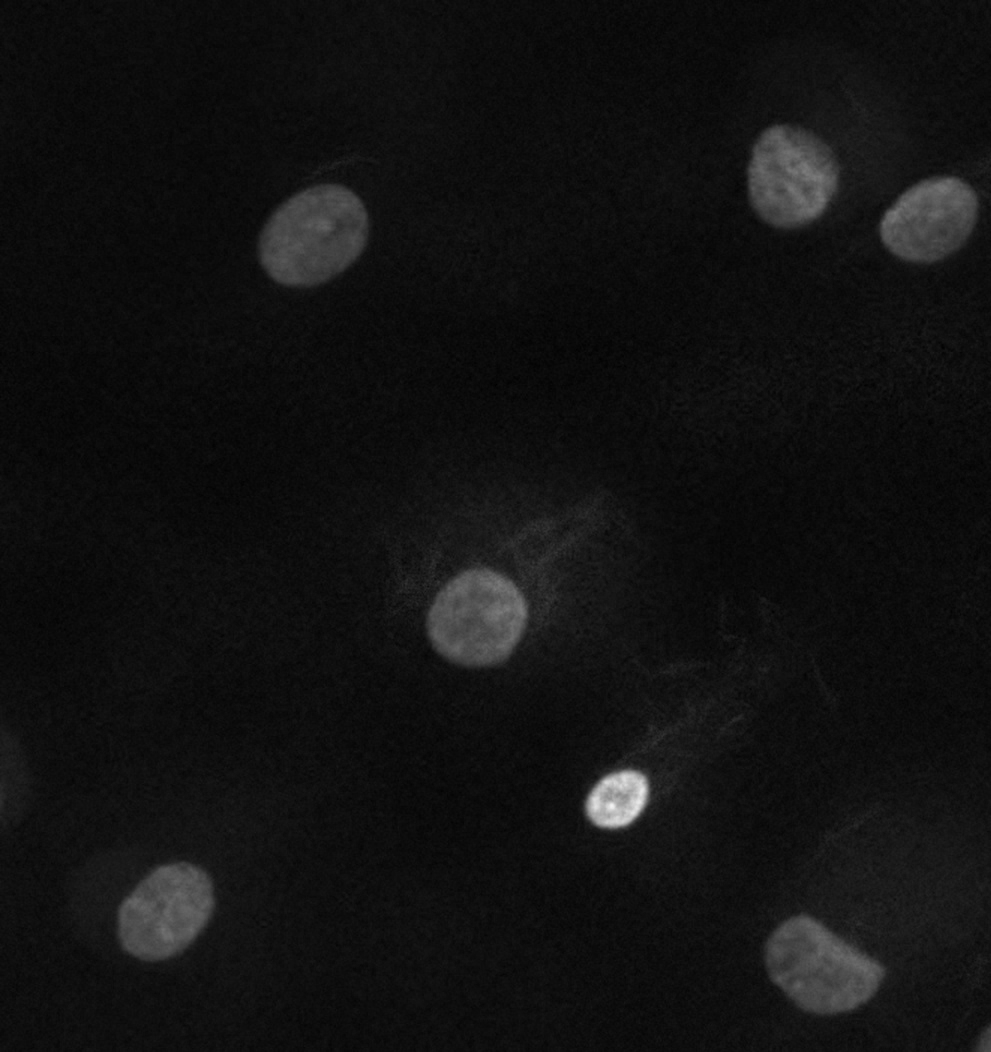

Supplement: Figure 2—source data 1. [file elife-84070-fig2-data1.zip › Figure 2 source data 1/LAMP-1/LAMP-1 120h DAPI.tif]

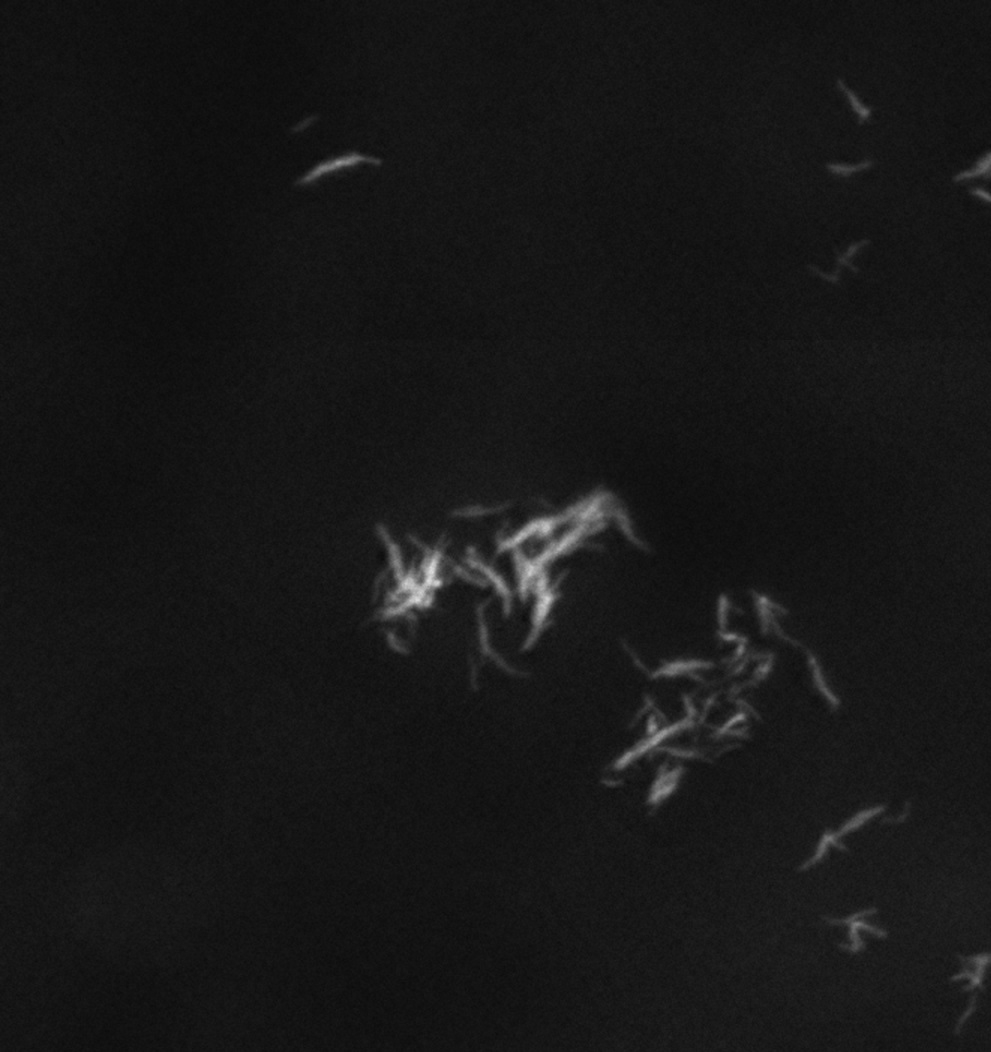

Supplement: Figure 2—source data 1. [file elife-84070-fig2-data1.zip › Figure 2 source data 1/LAMP-1/LAMP-1 120h GFP.tif]

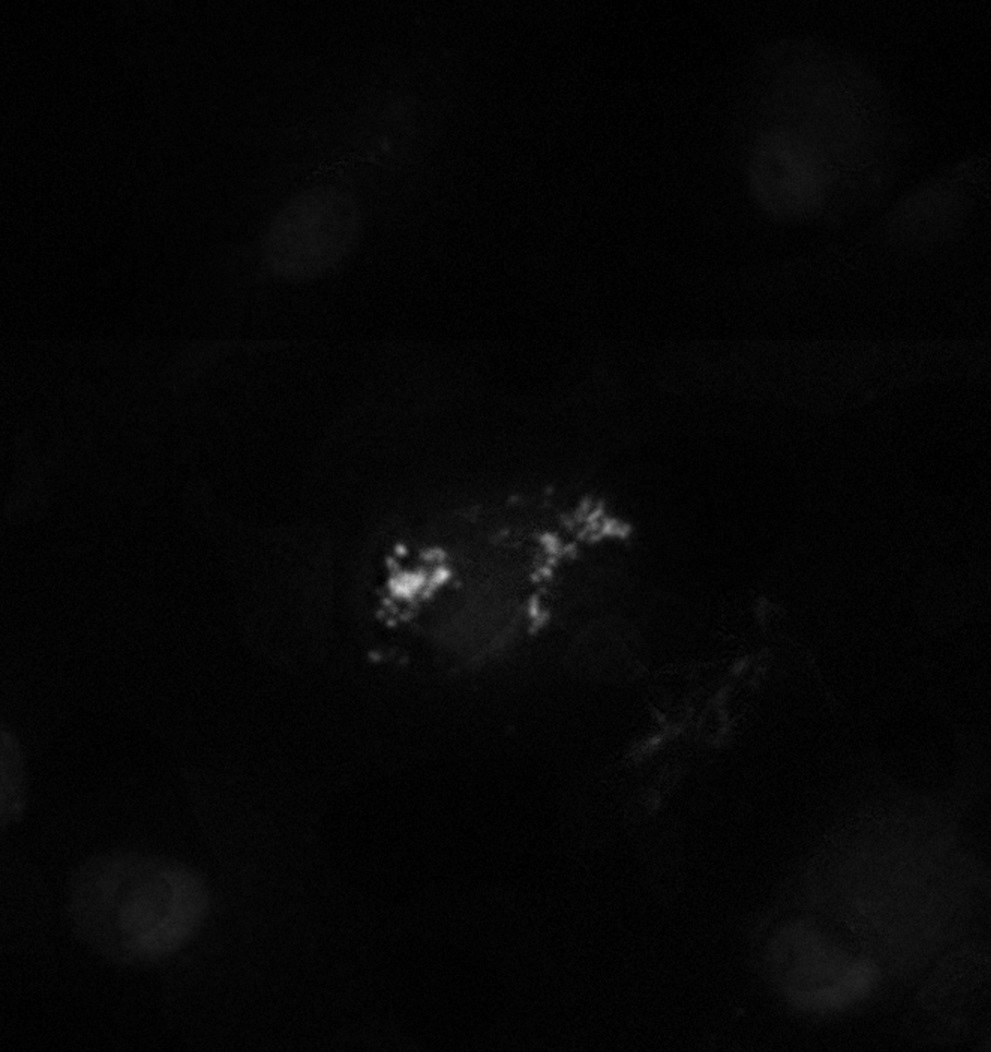

Supplement: Figure 2—source data 1. [file elife-84070-fig2-data1.zip › Figure 2 source data 1/LAMP-1/LAMP-1 120h AF647.tif]

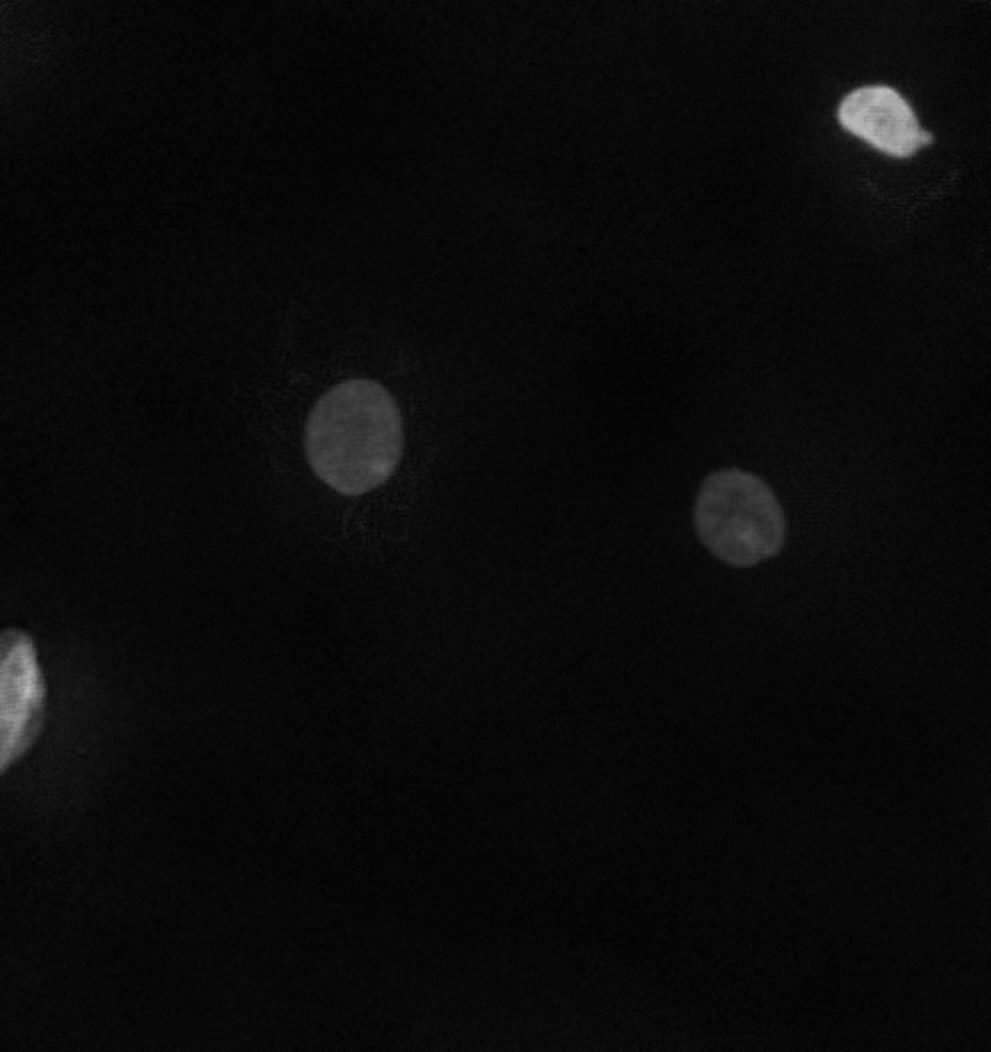

Supplement: Figure 2—source data 1. [file elife-84070-fig2-data1.zip › Figure 2 source data 1/LAMP-1/LAMP-1 72h DAPI.tif]

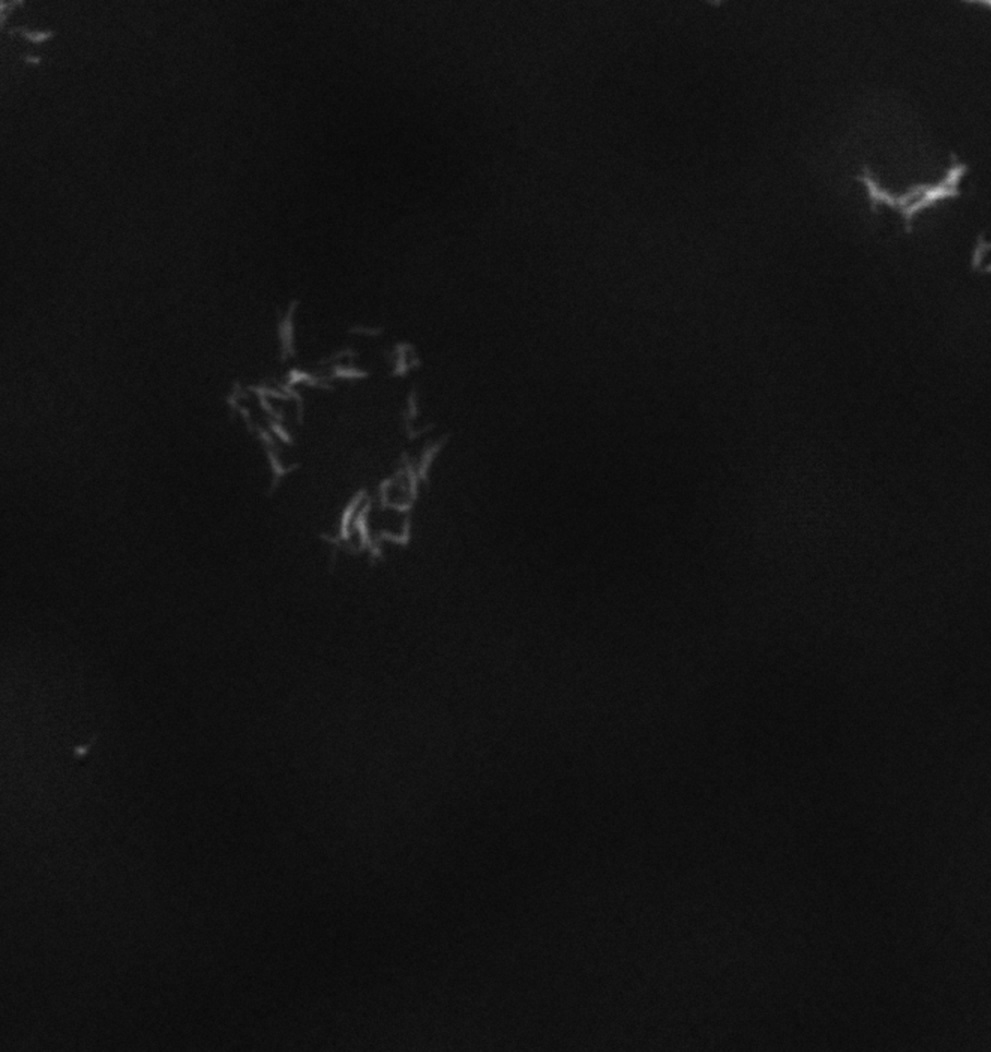

Supplement: Figure 2—source data 1. [file elife-84070-fig2-data1.zip › Figure 2 source data 1/LAMP-1/LAMP-1 72h GFP.tif]

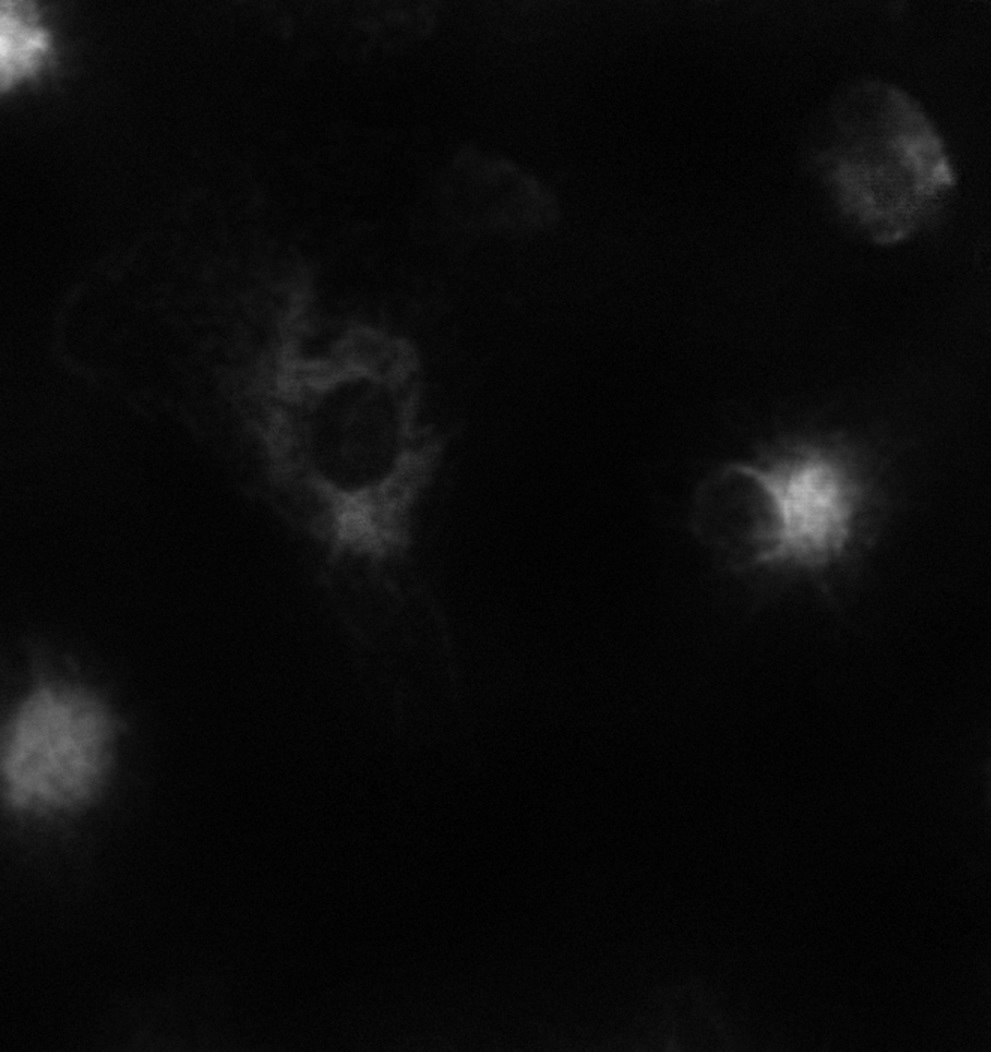

Supplement: Figure 2—source data 1. [file elife-84070-fig2-data1.zip › Figure 2 source data 1/LAMP-1/LAMP-1 72h AF647.tif]

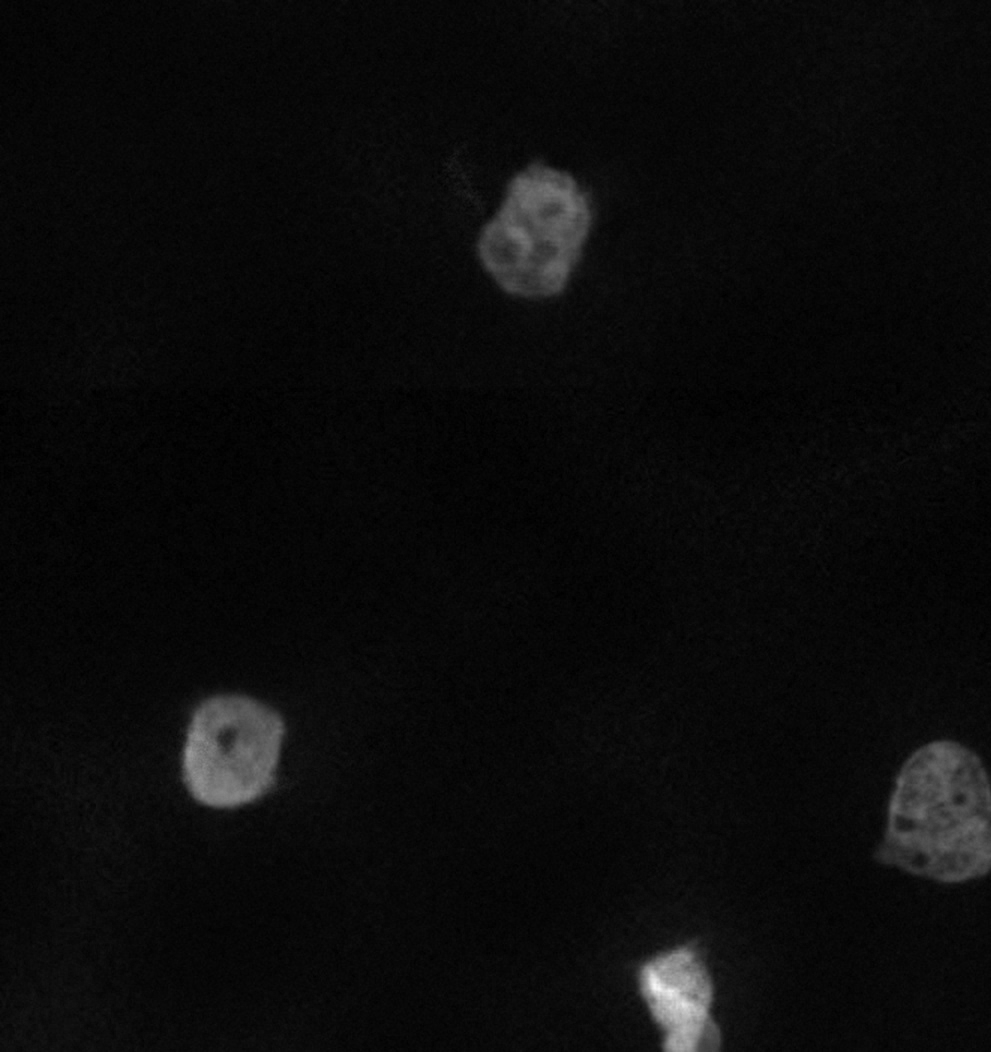

Supplement: Figure 2—source data 1. [file elife-84070-fig2-data1.zip › Figure 2 source data 1/LAMP-1/LAMP-1 24h DAPI.tif]

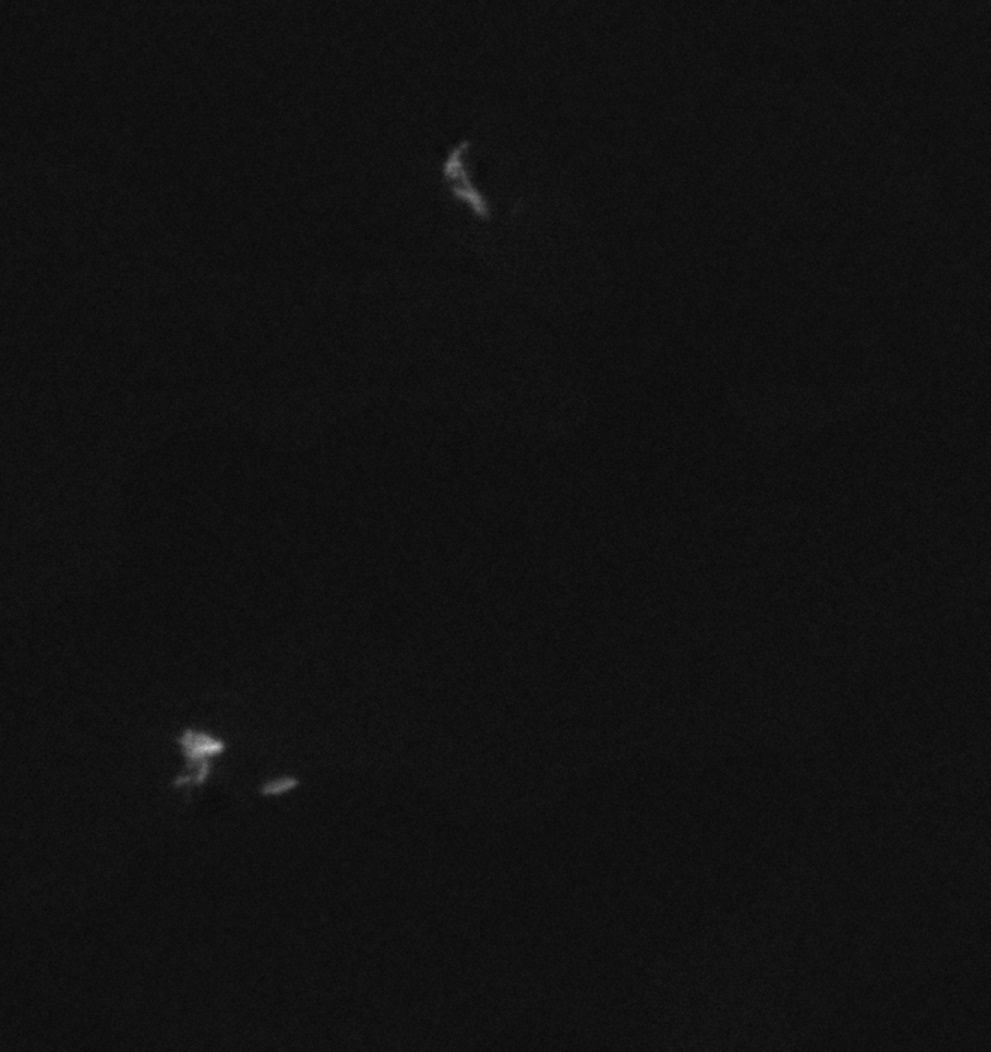

Supplement: Figure 2—source data 1. [file elife-84070-fig2-data1.zip › Figure 2 source data 1/LAMP-1/LAMP-1 24h GFP.tif]

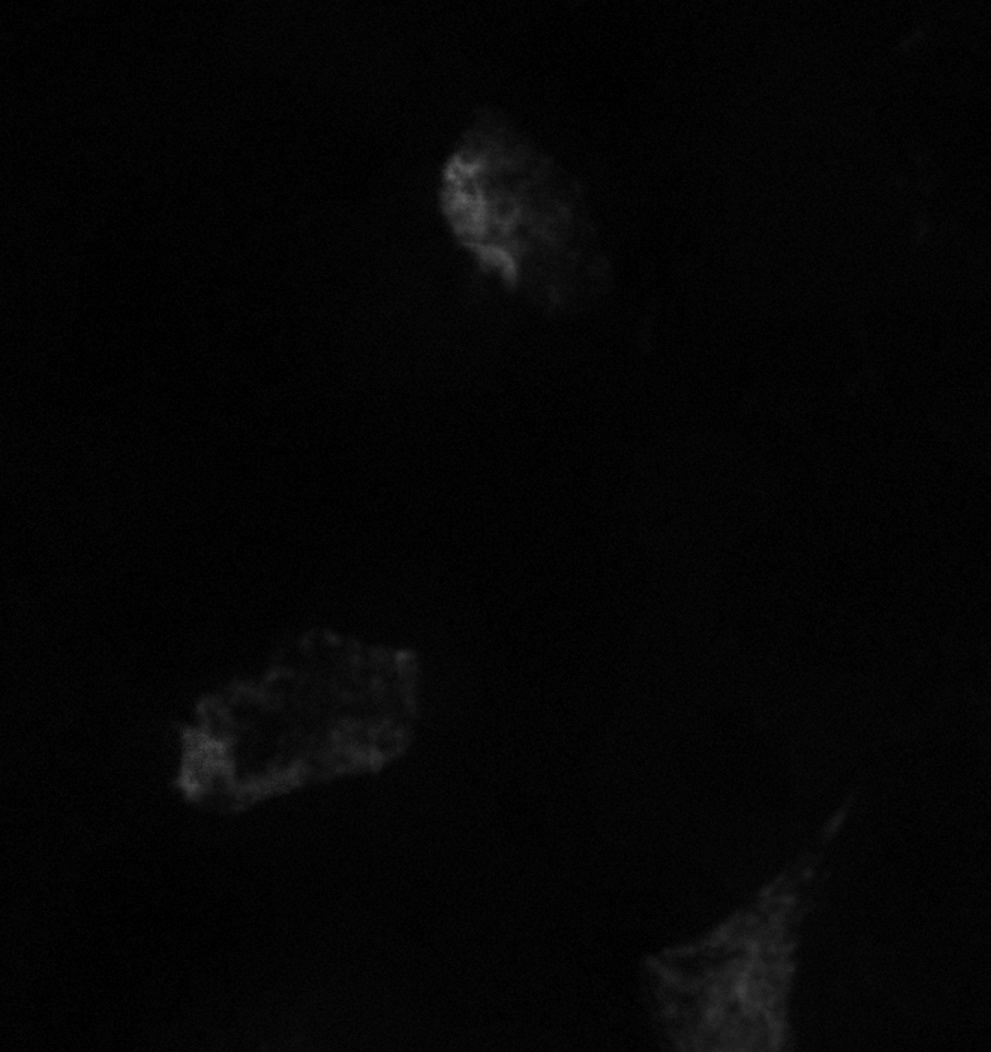

Supplement: Figure 2—source data 1. [file elife-84070-fig2-data1.zip › Figure 2 source data 1/LAMP-1/LAMP-1 24h AF647.tif]

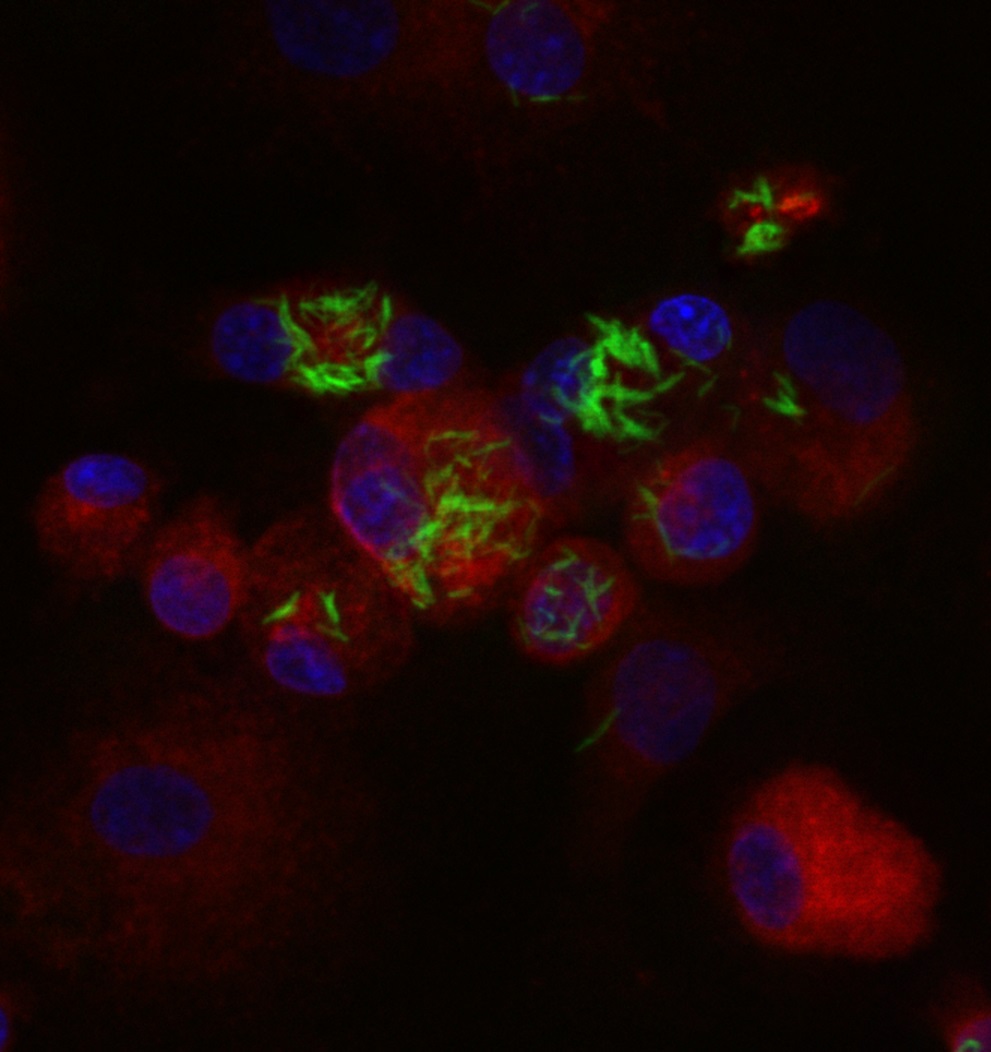

Supplement: Figure 2—source data 1. [file elife-84070-fig2-data1.zip › Figure 2 source data 1/MHC-I/MHC-I 120h.JPG]

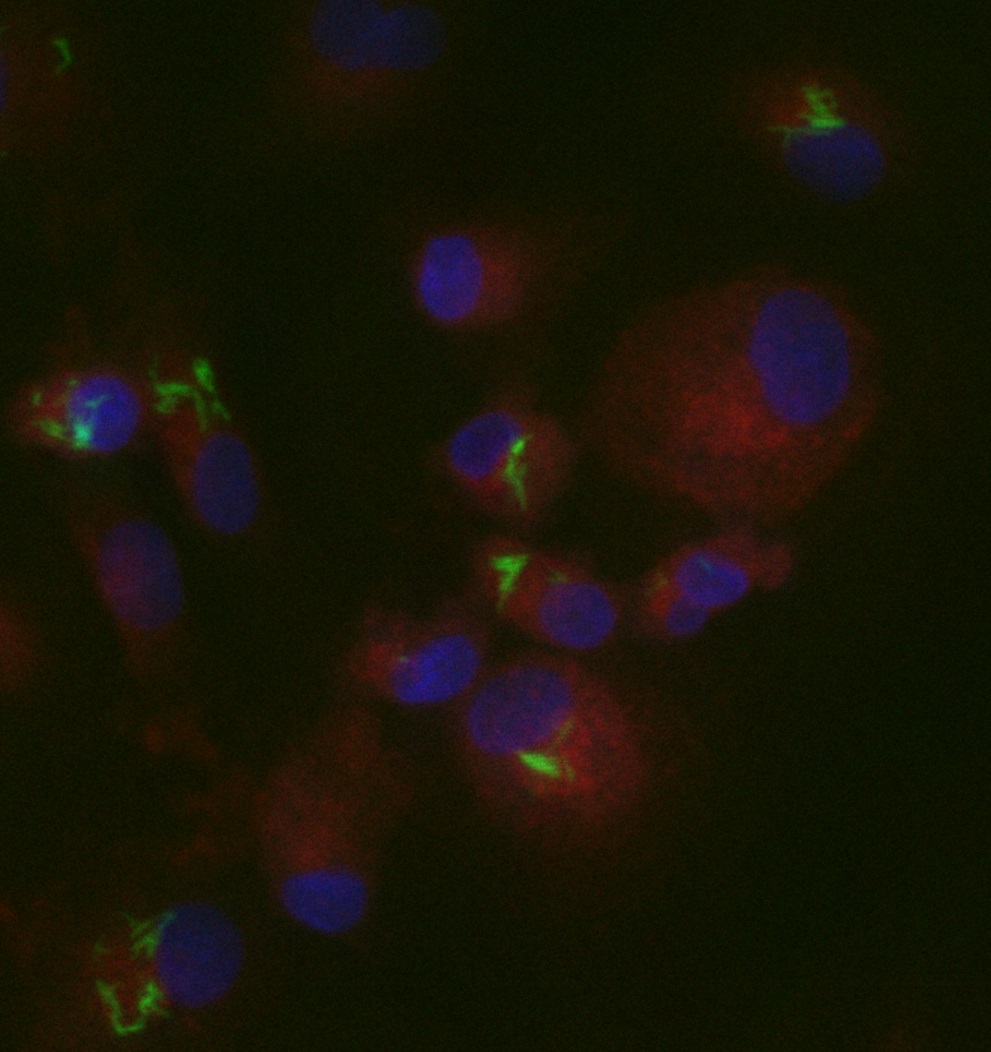

Supplement: Figure 2—source data 1. [file elife-84070-fig2-data1.zip › Figure 2 source data 1/MHC-I/MHC-I 24h.JPG]

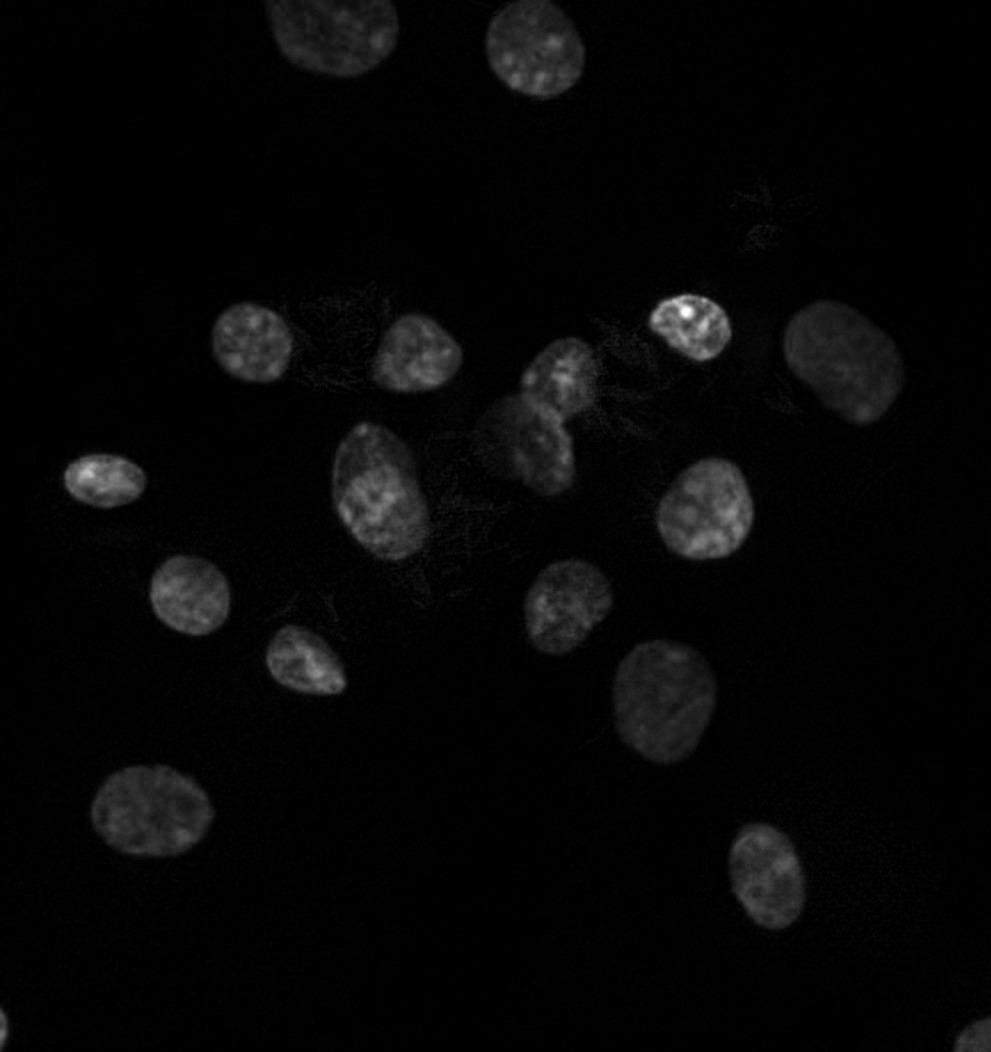

Supplement: Figure 2—source data 1. [file elife-84070-fig2-data1.zip › Figure 2 source data 1/MHC-I/MHC-I 120h DAPI.tif]

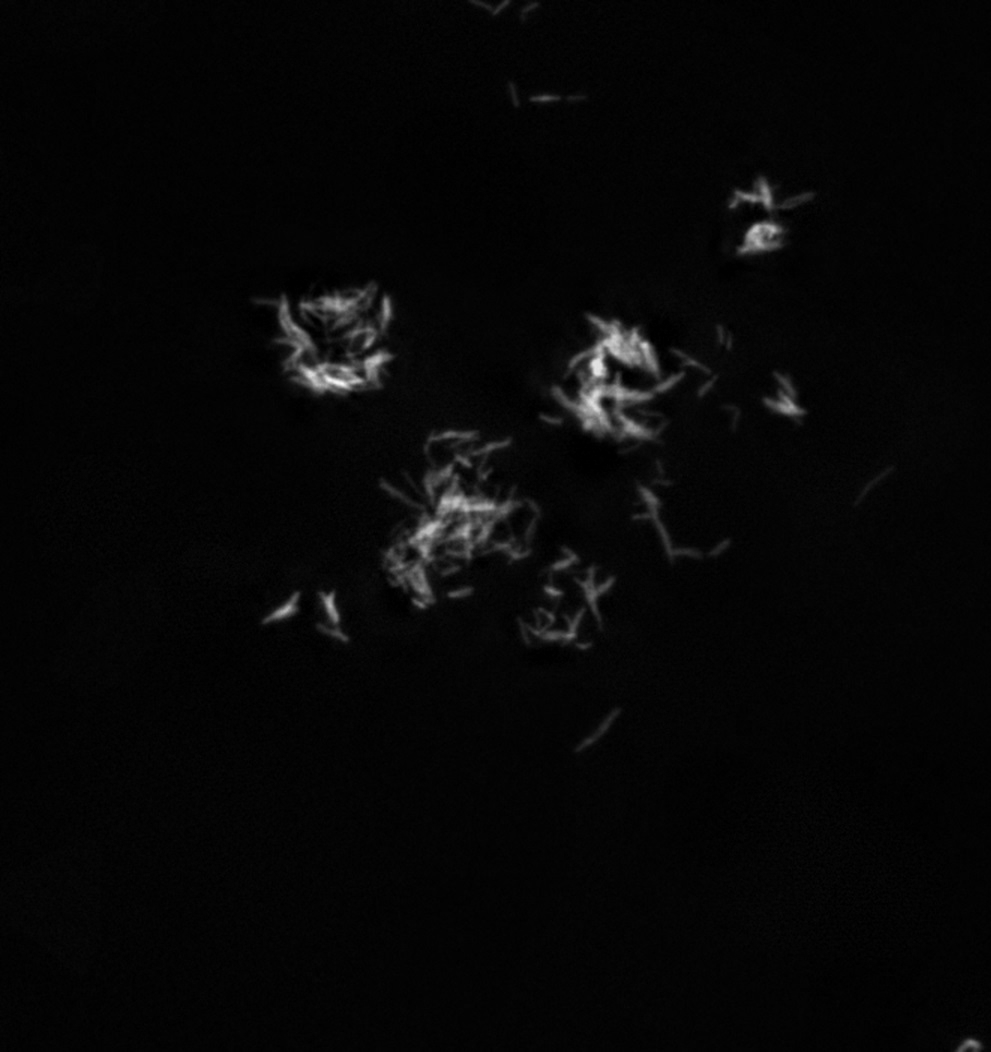

Supplement: Figure 2—source data 1. [file elife-84070-fig2-data1.zip › Figure 2 source data 1/MHC-I/MHC-I 120h GFP.tif]

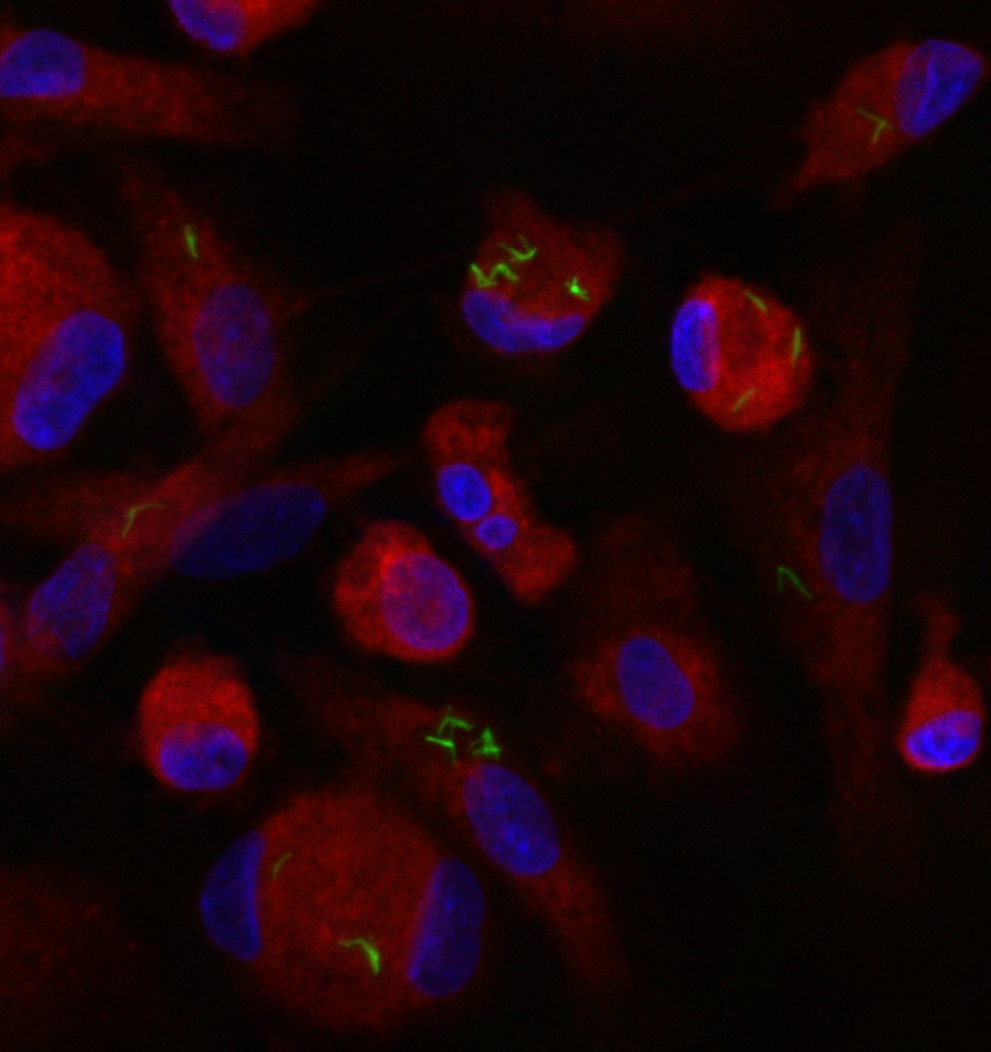

Supplement: Figure 2—source data 1. [file elife-84070-fig2-data1.zip › Figure 2 source data 1/MHC-I/MHC-I 72h.JPG]

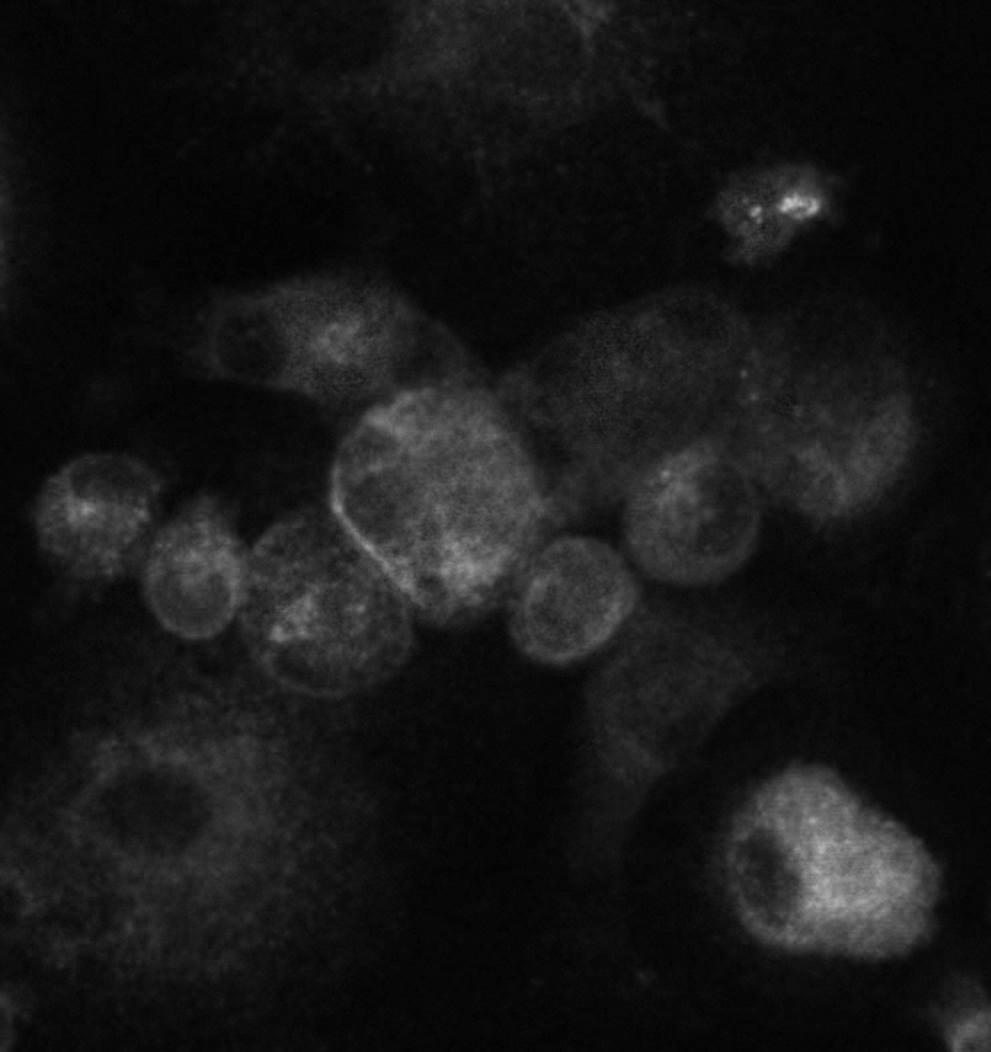

Supplement: Figure 2—source data 1. [file elife-84070-fig2-data1.zip › Figure 2 source data 1/MHC-I/MHC-I 120h AF647.tif]

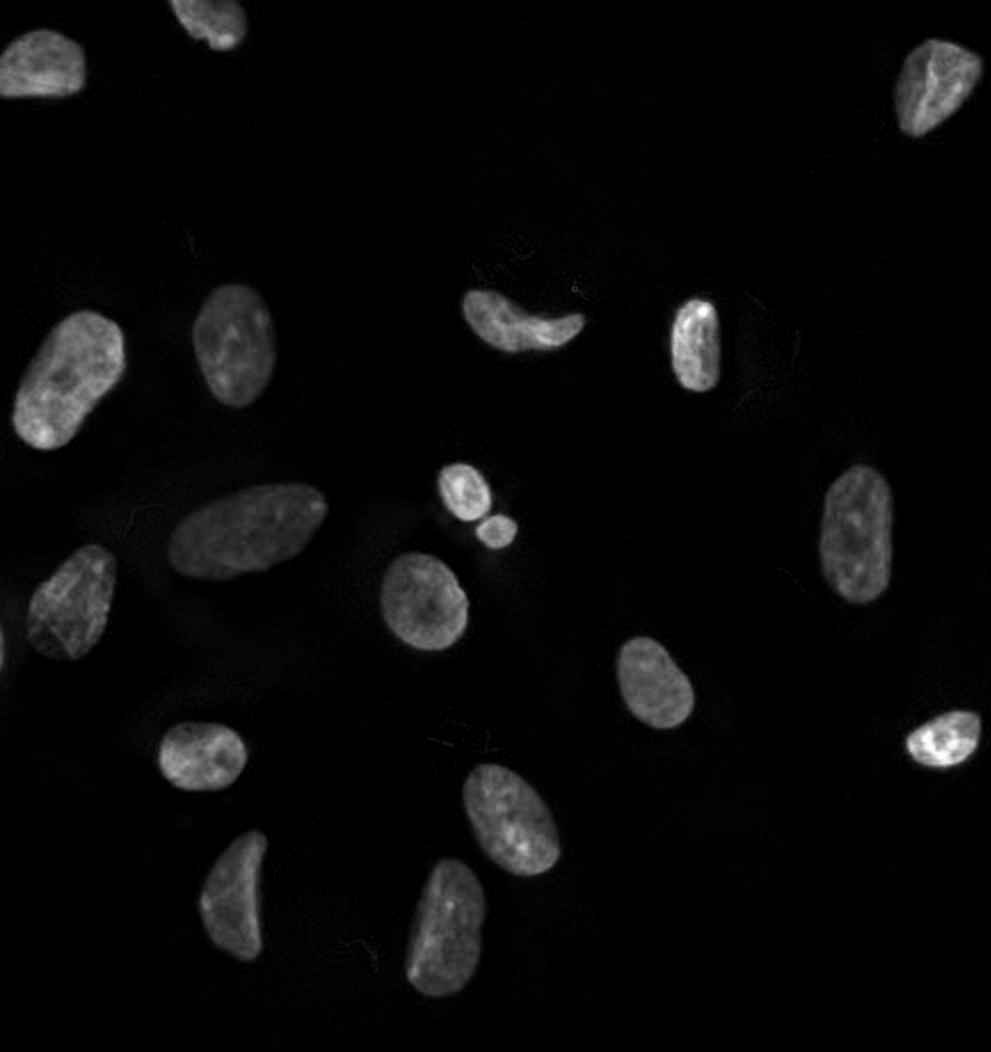

Supplement: Figure 2—source data 1. [file elife-84070-fig2-data1.zip › Figure 2 source data 1/MHC-I/MHC-I 72h DAPI.tif]

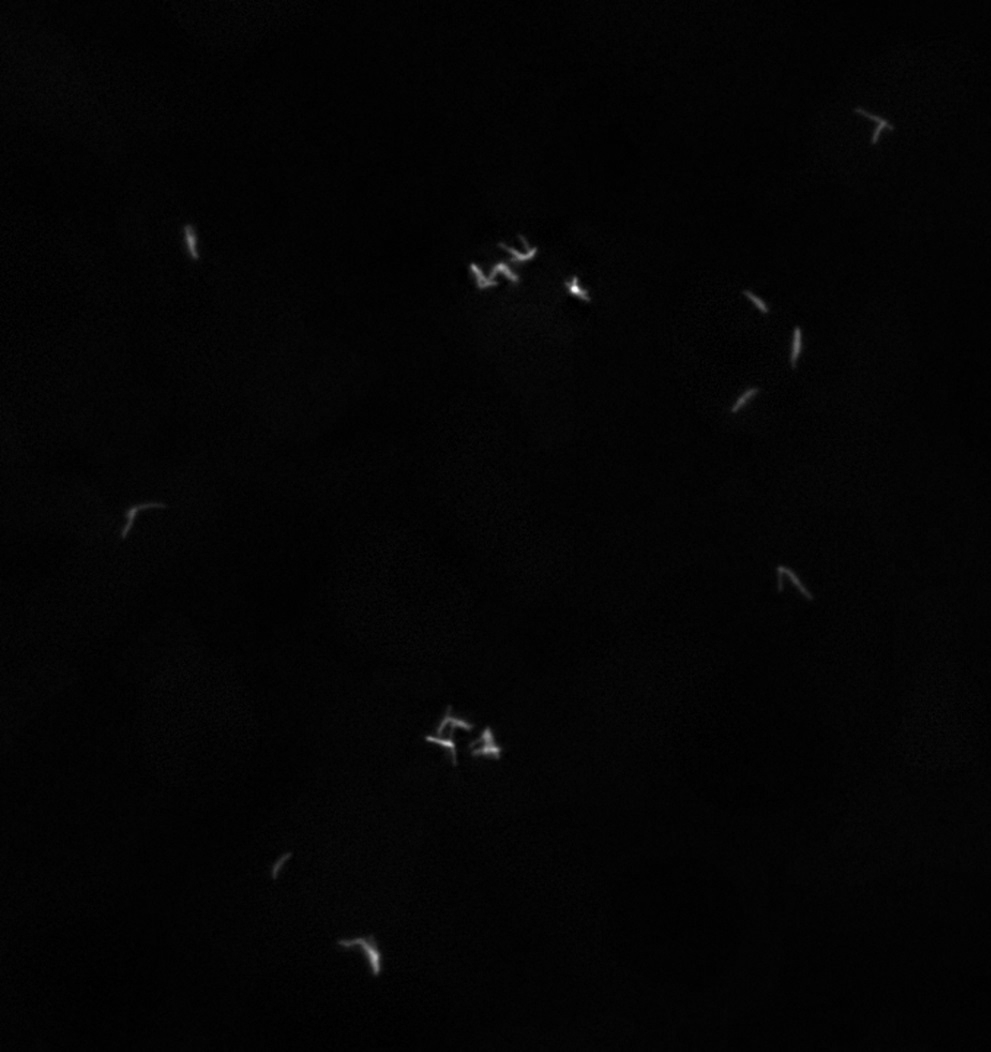

Supplement: Figure 2—source data 1. [file elife-84070-fig2-data1.zip › Figure 2 source data 1/MHC-I/MHC-I 72h GFP.tif]

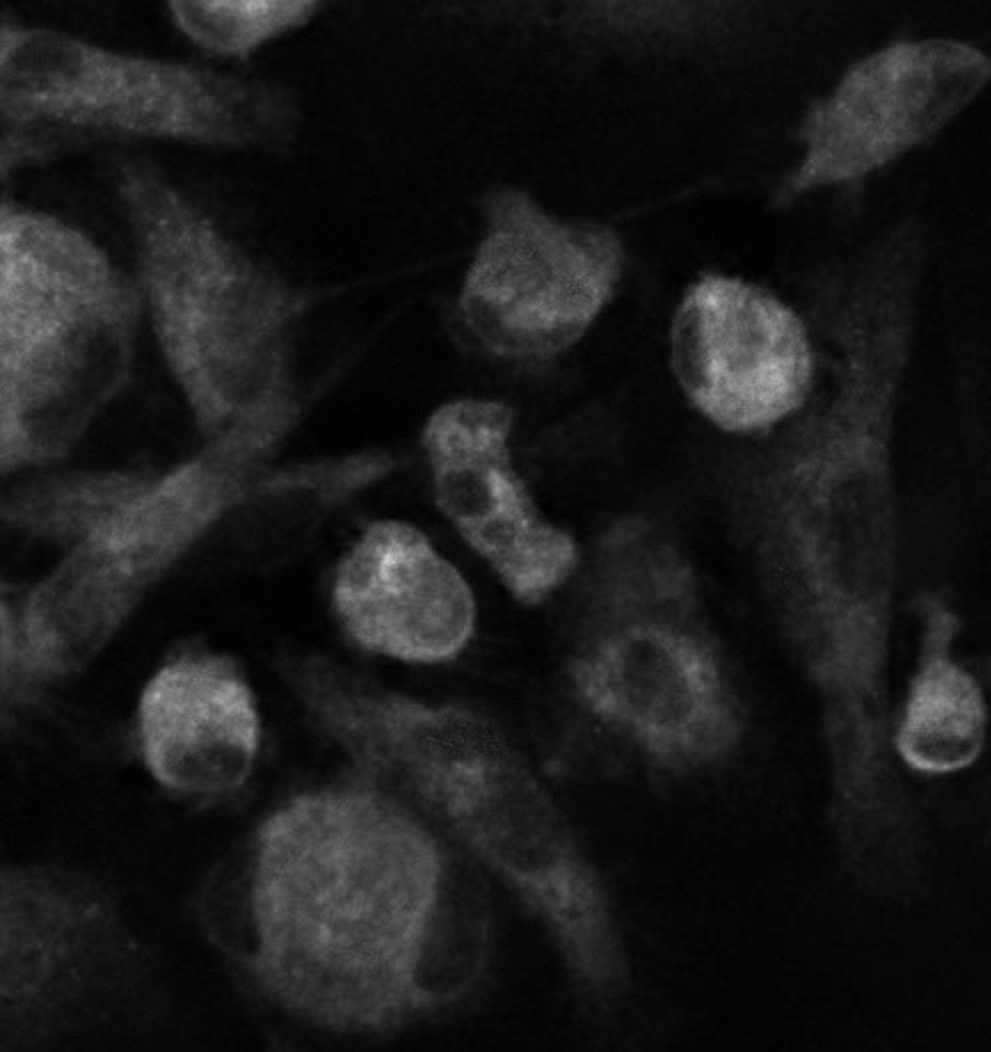

Supplement: Figure 2—source data 1. [file elife-84070-fig2-data1.zip › Figure 2 source data 1/MHC-I/MHC-I 72h AF647.tif]

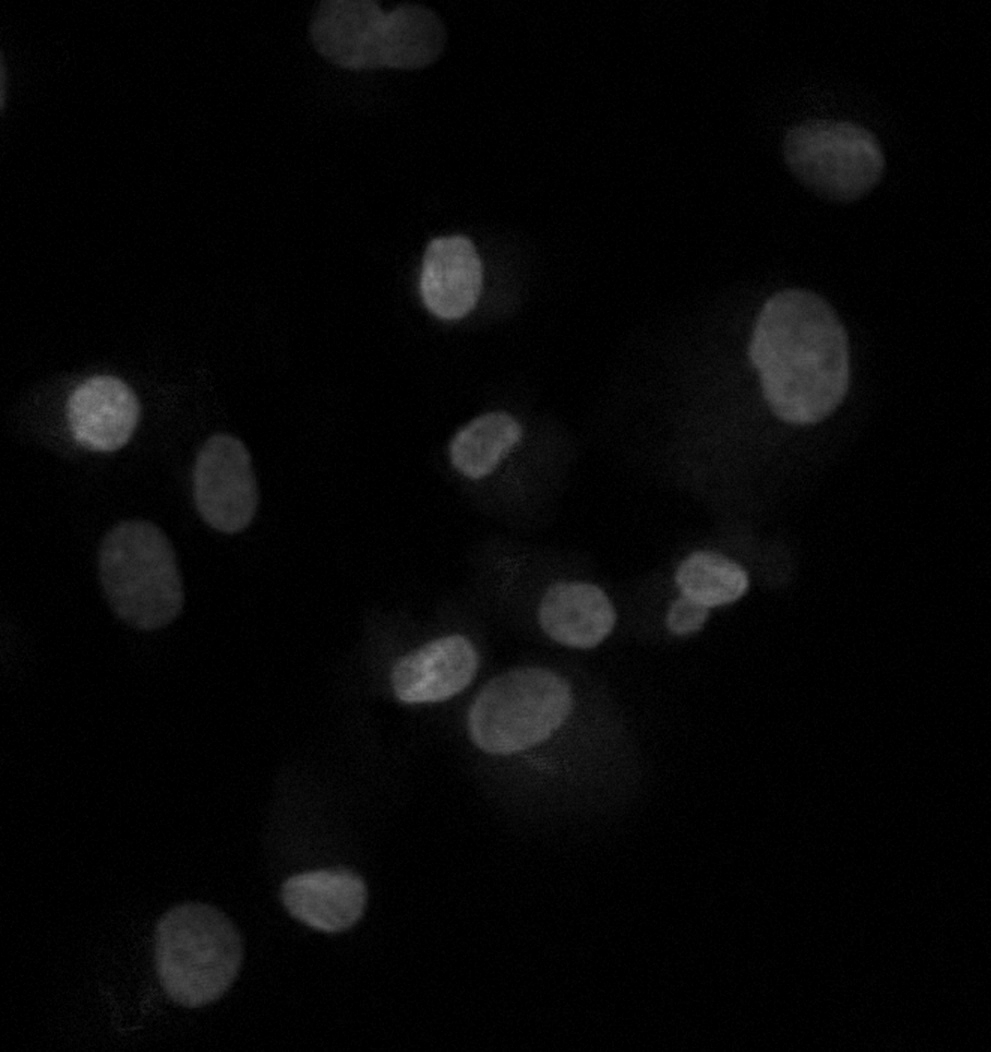

Supplement: Figure 2—source data 1. [file elife-84070-fig2-data1.zip › Figure 2 source data 1/MHC-I/MHC-I 24h DAPI.tif]

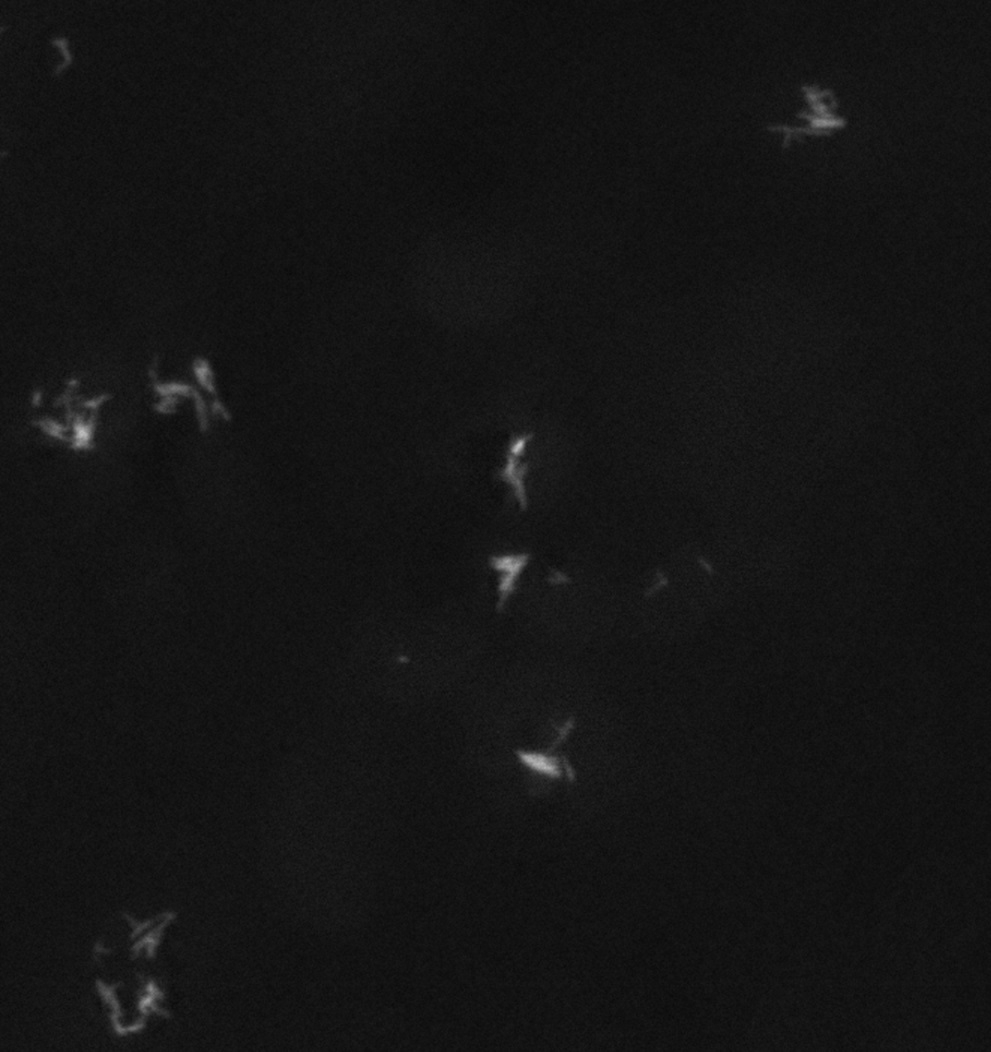

Supplement: Figure 2—source data 1. [file elife-84070-fig2-data1.zip › Figure 2 source data 1/MHC-I/MHC-I 24h GFP.tif]

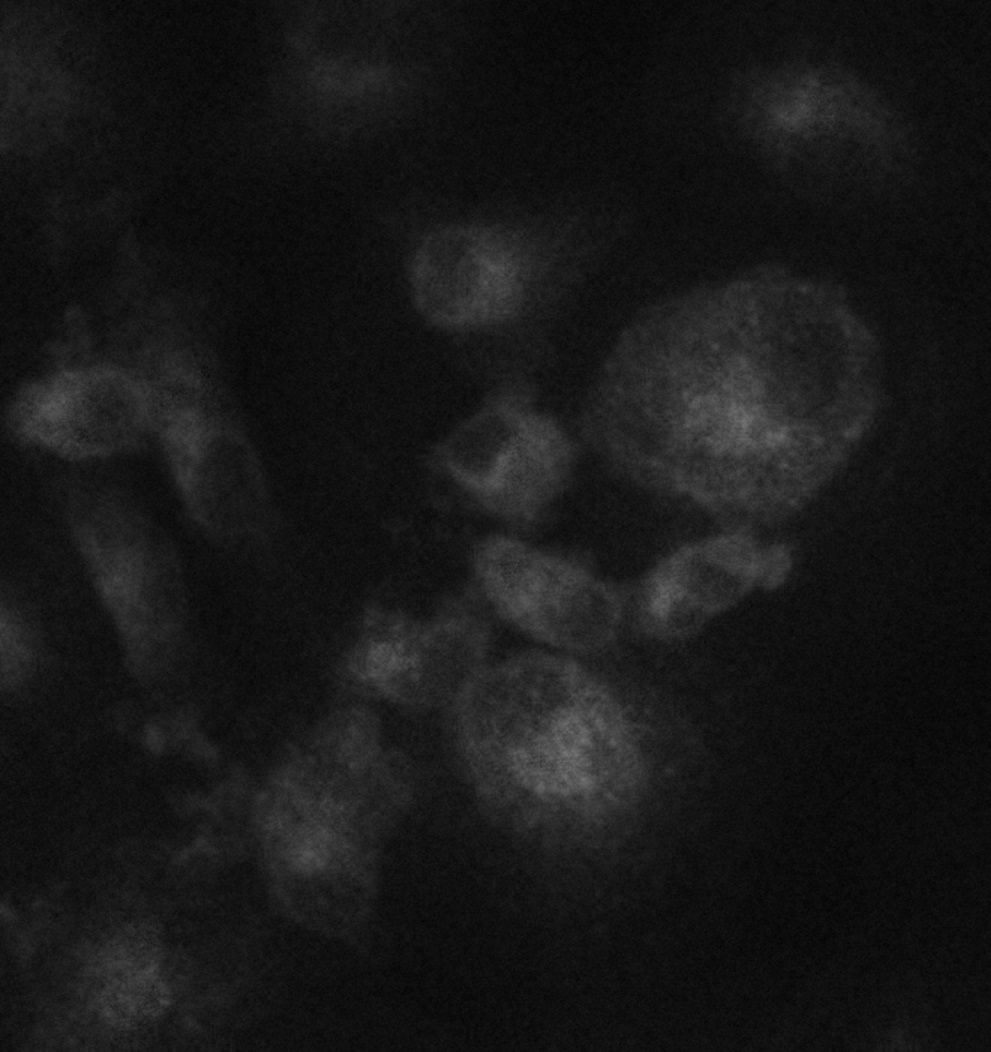

Supplement: Figure 2—source data 1. [file elife-84070-fig2-data1.zip › Figure 2 source data 1/MHC-I/MHC-I 24h AF647.tif]

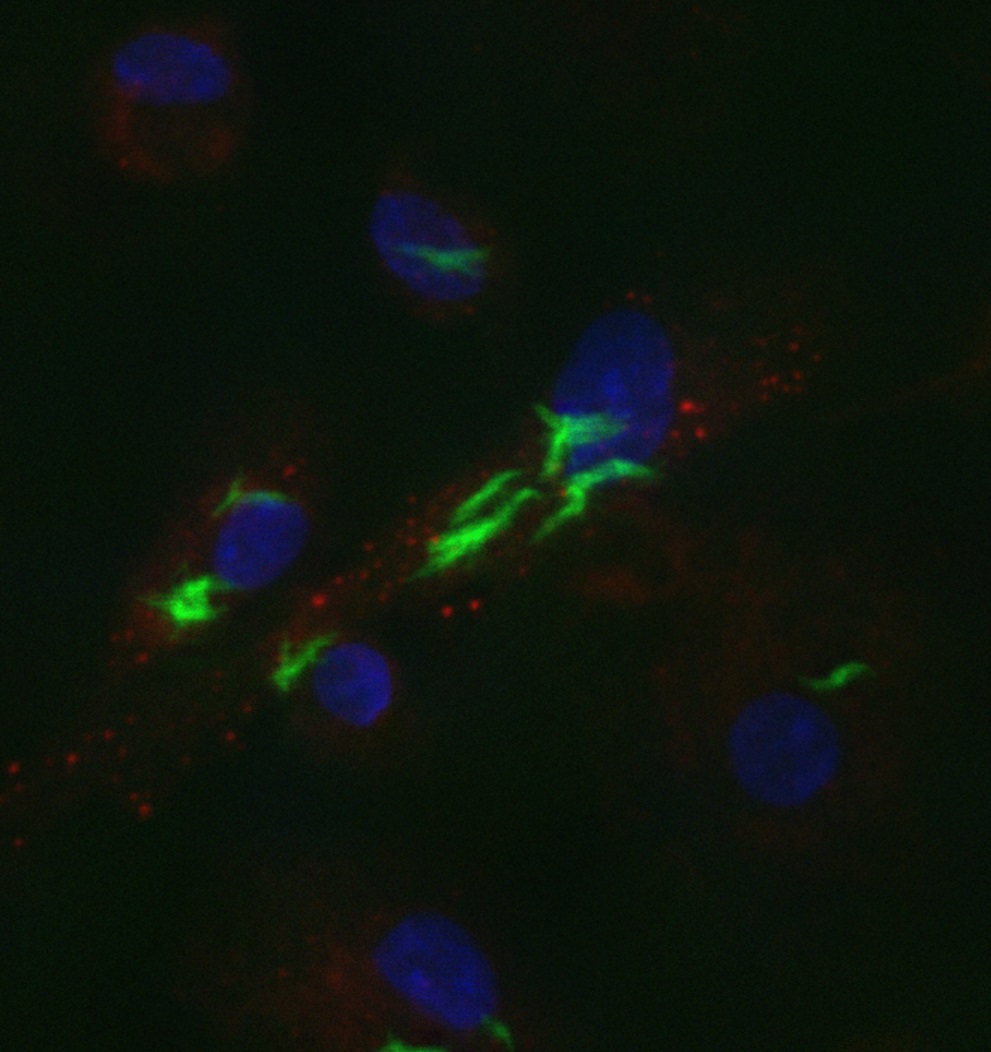

Supplement: Figure 2—source data 1. [file elife-84070-fig2-data1.zip › Figure 2 source data 1/P62/P62 ESX 120h.JPG]

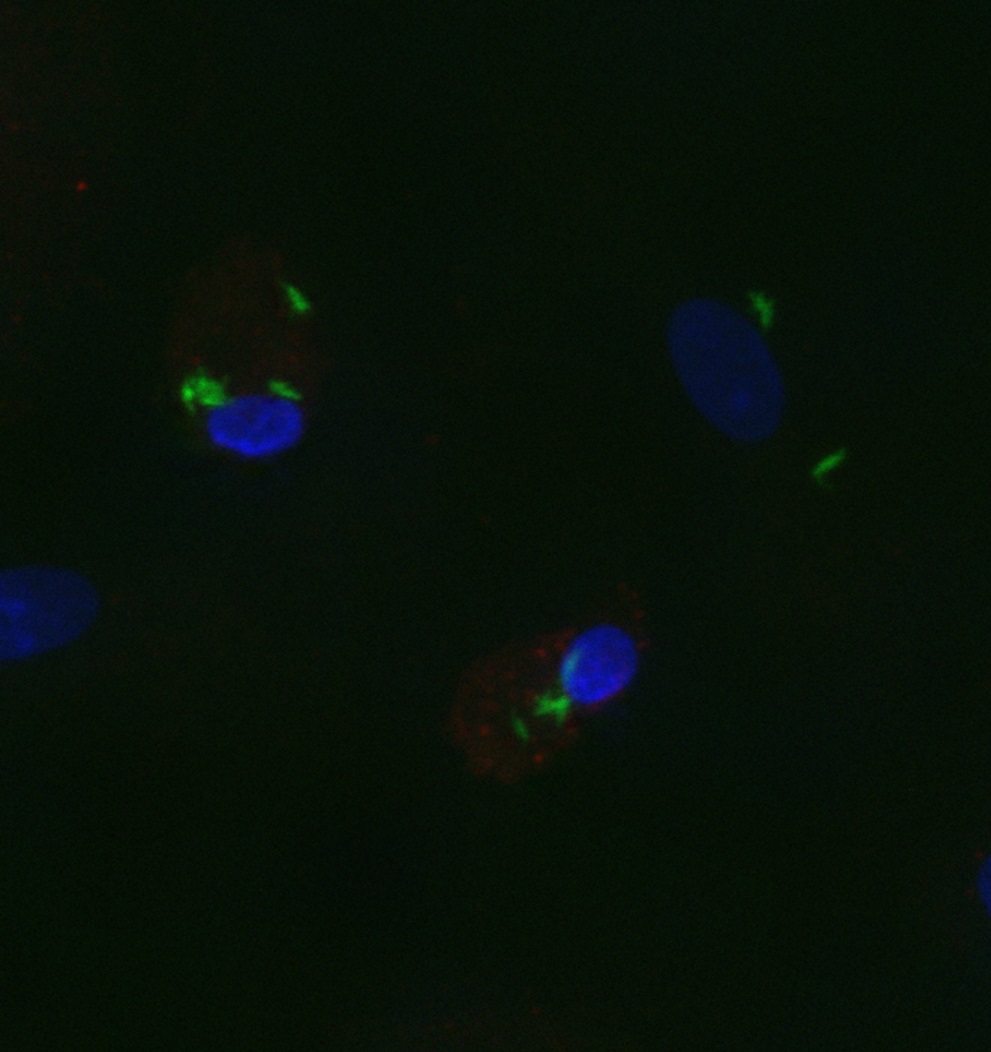

Supplement: Figure 2—source data 1. [file elife-84070-fig2-data1.zip › Figure 2 source data 1/P62/P62 ESX 24h.JPG]

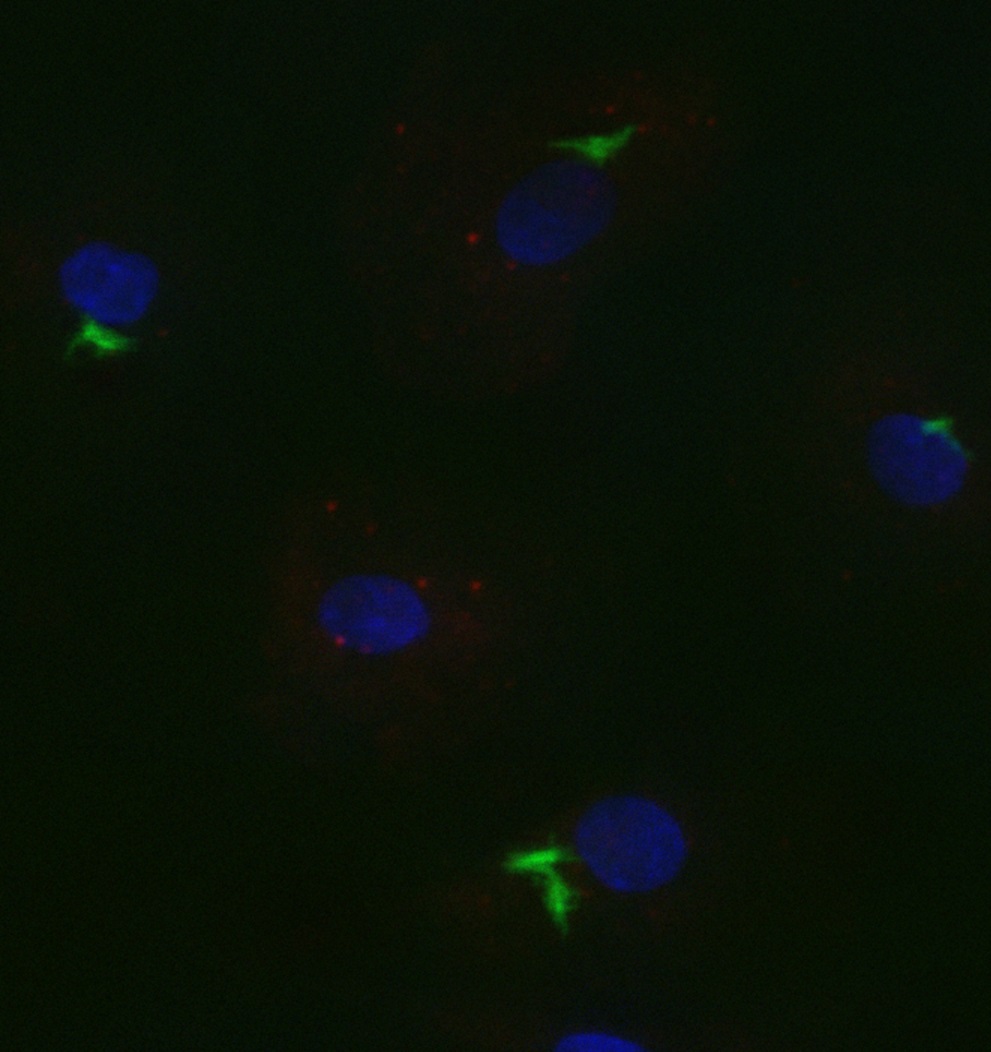

Supplement: Figure 2—source data 1. [file elife-84070-fig2-data1.zip › Figure 2 source data 1/P62/P62 ESX 72h.JPG]

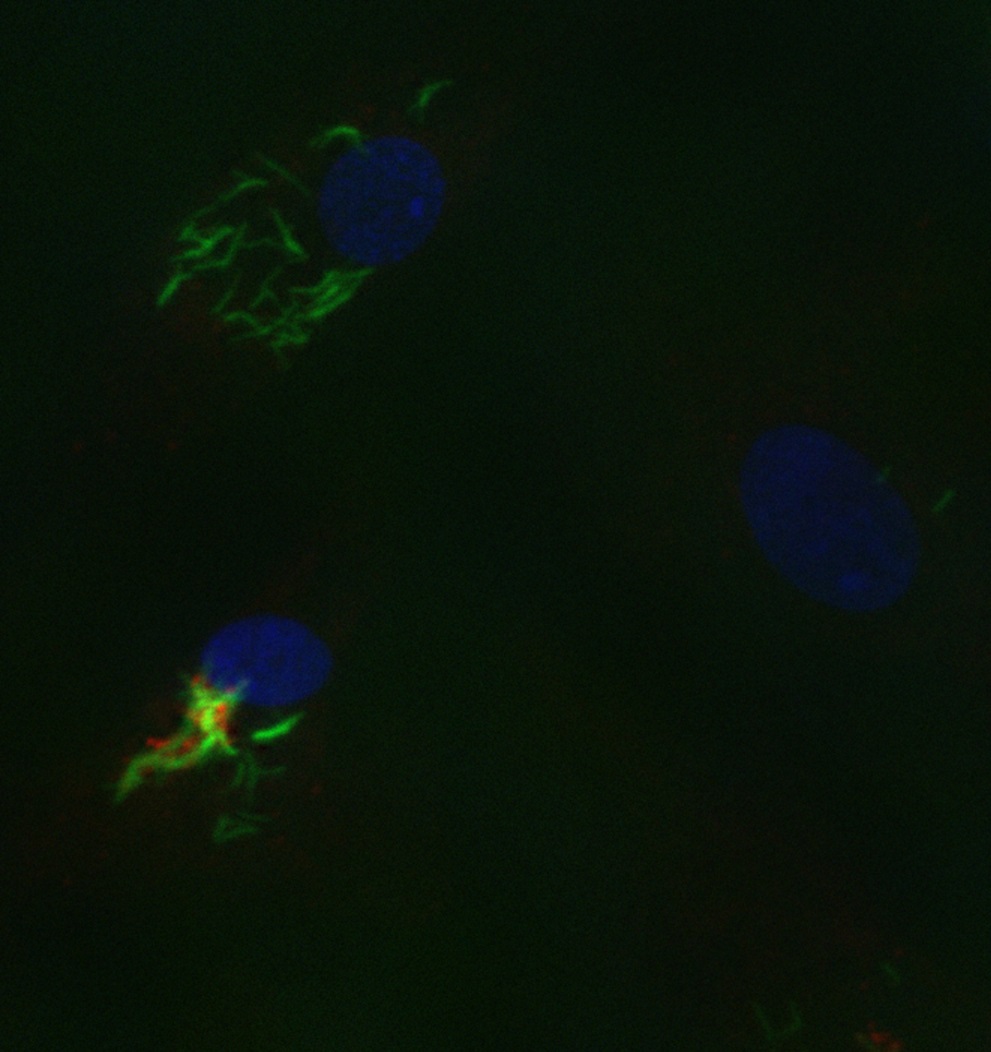

Supplement: Figure 2—source data 1. [file elife-84070-fig2-data1.zip › Figure 2 source data 1/P62/P62 WT 120h 2.JPG]

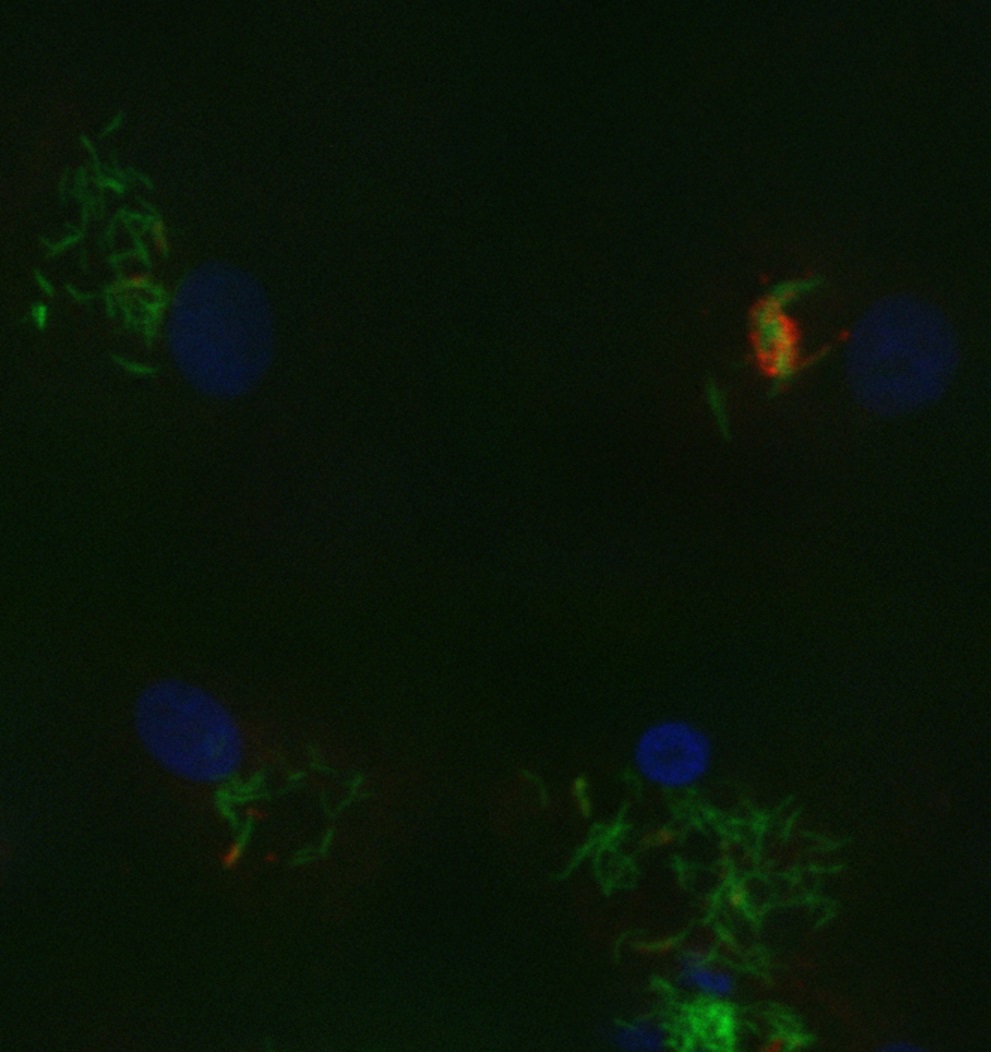

Supplement: Figure 2—source data 1. [file elife-84070-fig2-data1.zip › Figure 2 source data 1/P62/P62 WT 120h.JPG]

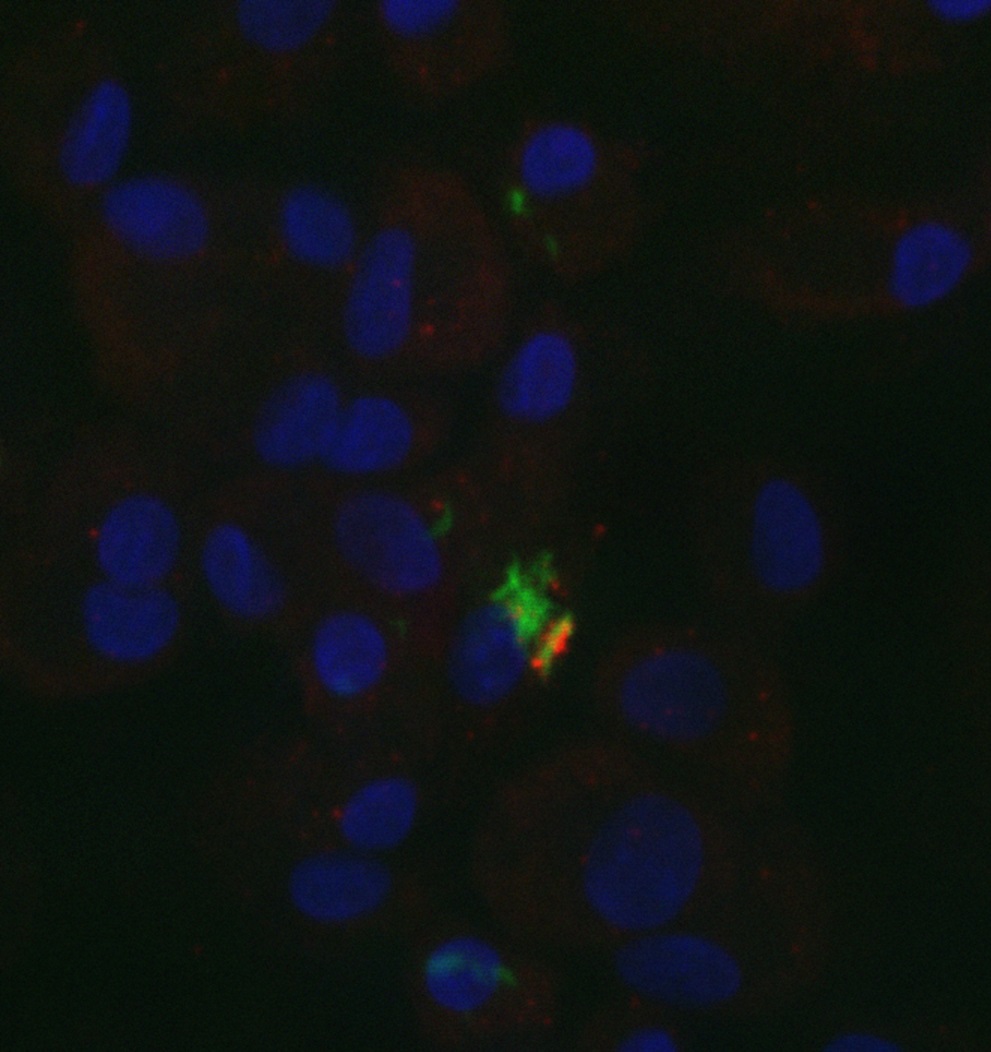

Supplement: Figure 2—source data 1. [file elife-84070-fig2-data1.zip › Figure 2 source data 1/P62/P62 WT 24h 2.JPG]

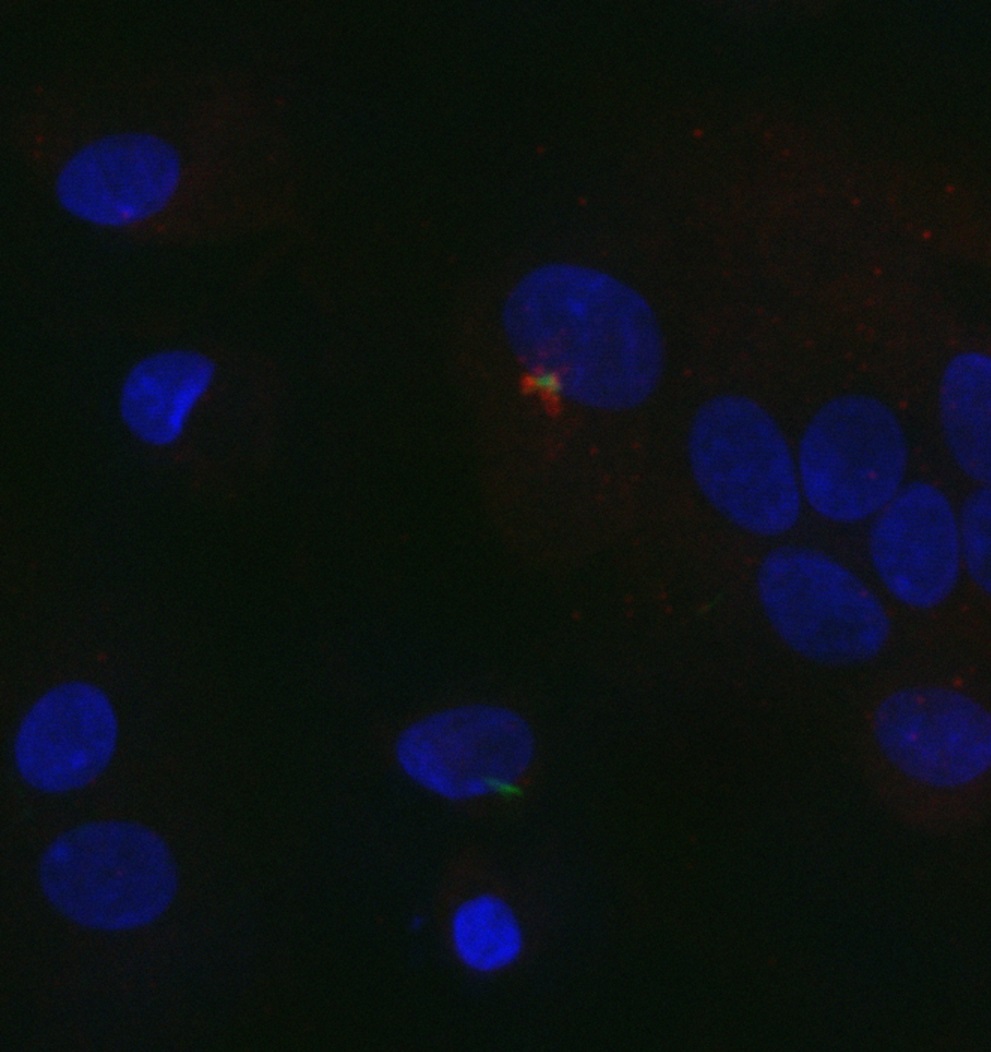

Supplement: Figure 2—source data 1. [file elife-84070-fig2-data1.zip › Figure 2 source data 1/P62/P62 WT 24h.JPG]

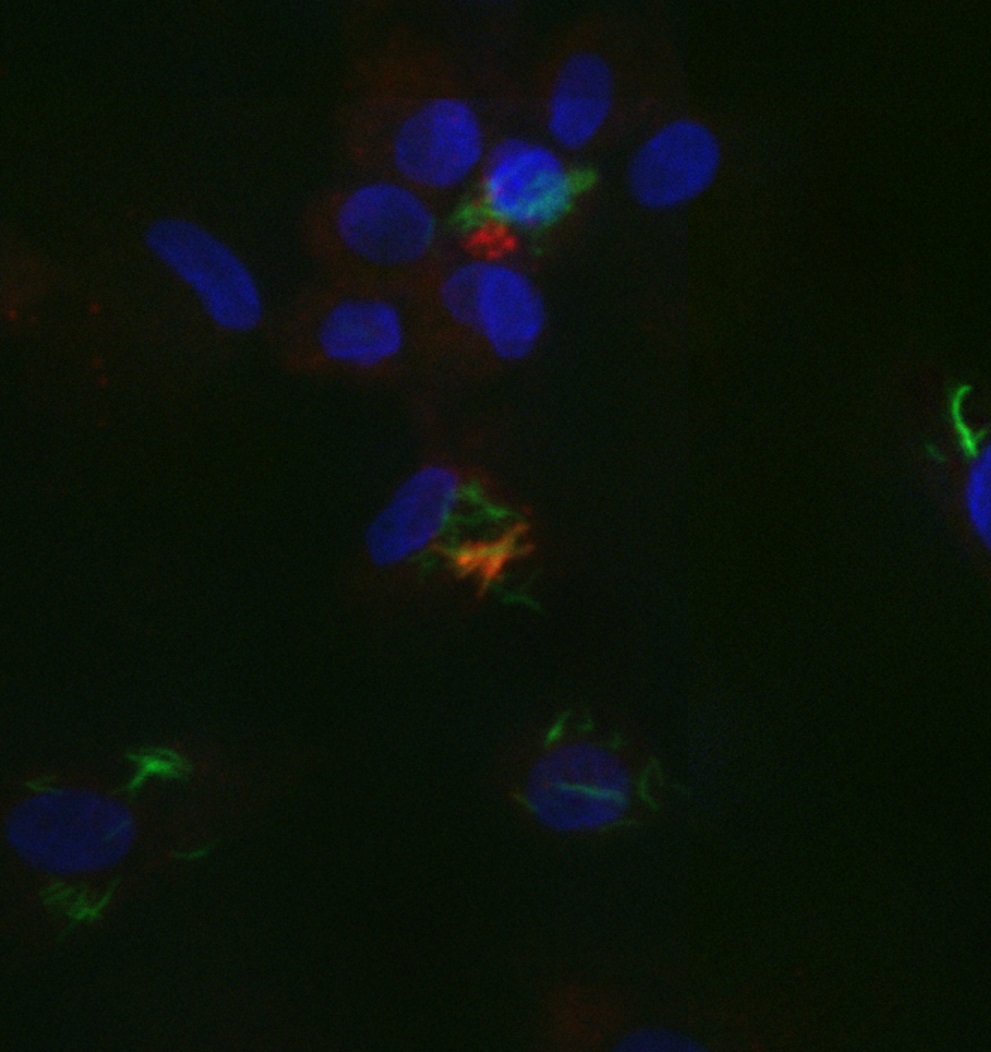

Supplement: Figure 2—source data 1. [file elife-84070-fig2-data1.zip › Figure 2 source data 1/P62/P62 WT 72h 2.JPG]

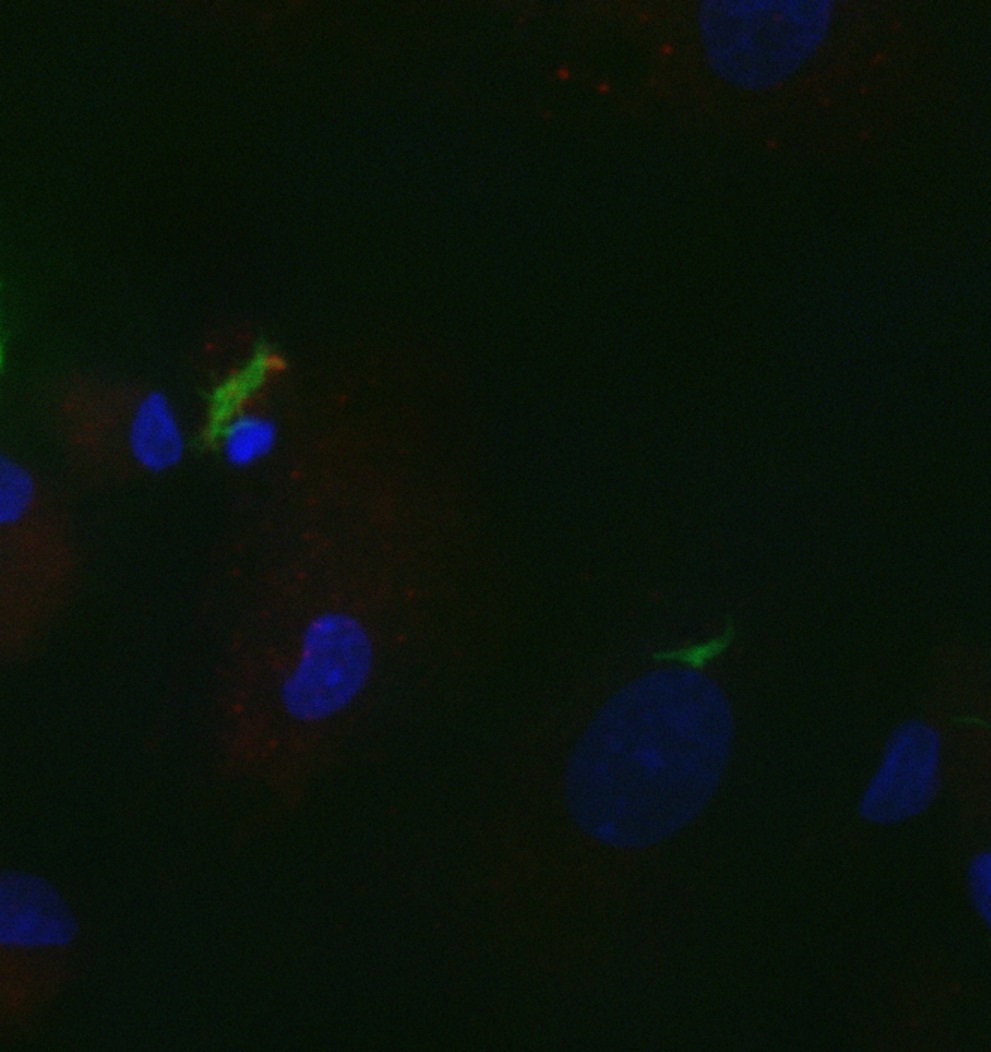

Supplement: Figure 2—source data 1. [file elife-84070-fig2-data1.zip › Figure 2 source data 1/P62/P62 WT 72h.JPG]

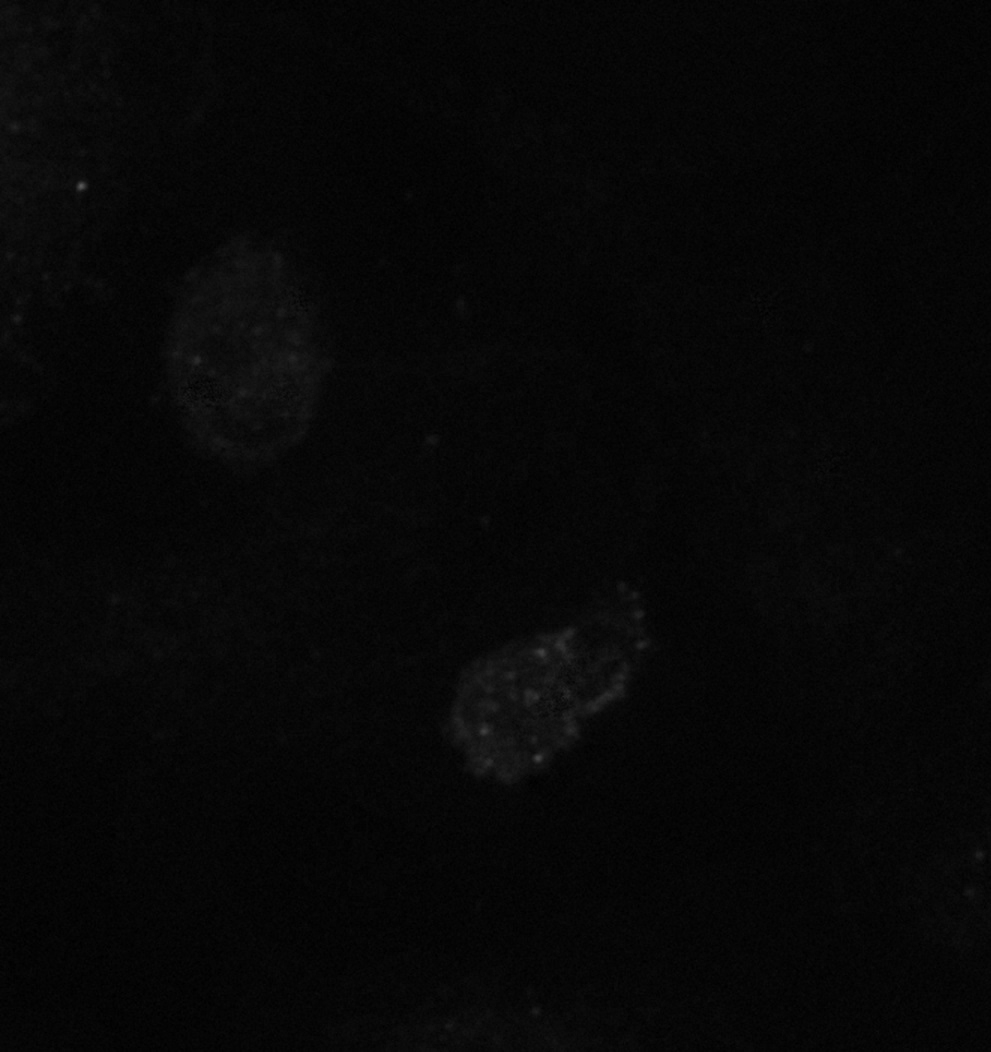

Supplement: Figure 2—source data 1. [file elife-84070-fig2-data1.zip › Figure 2 source data 1/P62/P62 ESX 24h AF647.tif]

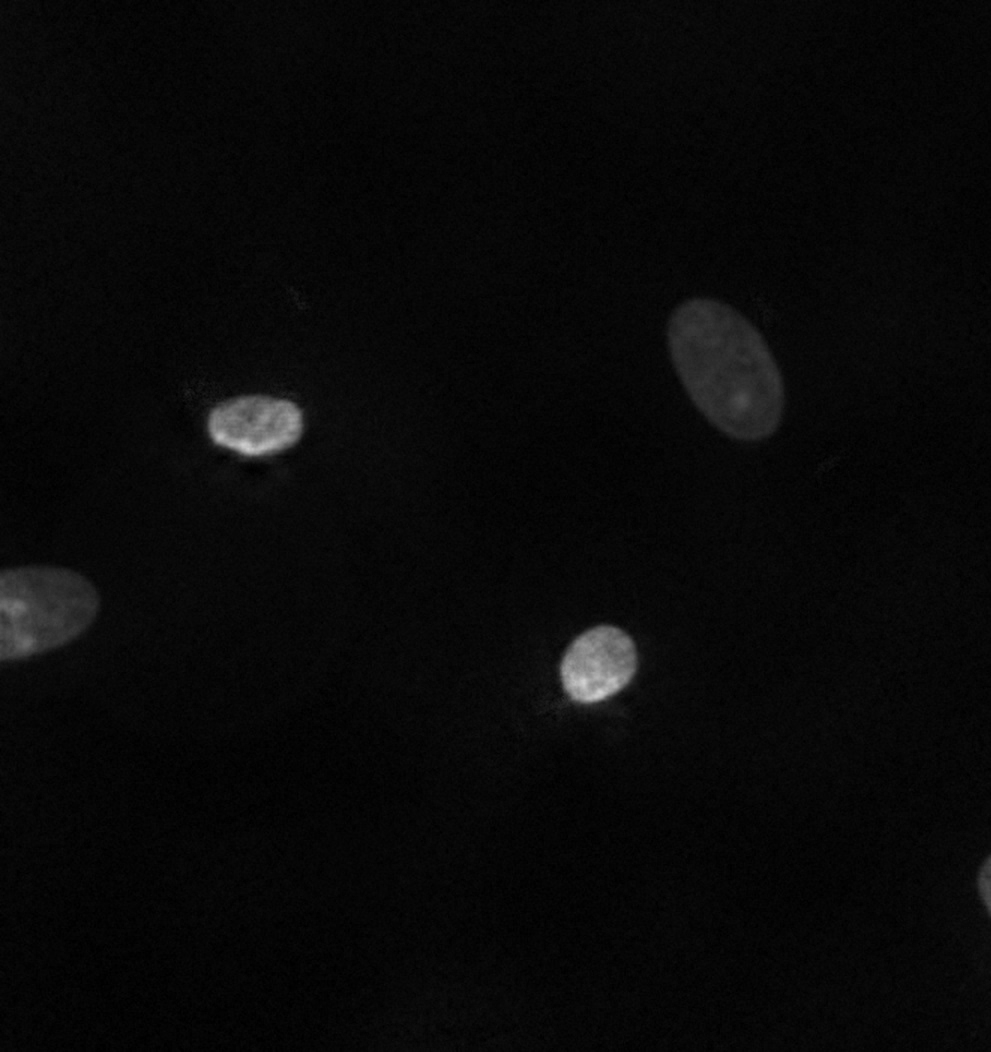

Supplement: Figure 2—source data 1. [file elife-84070-fig2-data1.zip › Figure 2 source data 1/P62/P62 ESX 24h DAPI.tif]

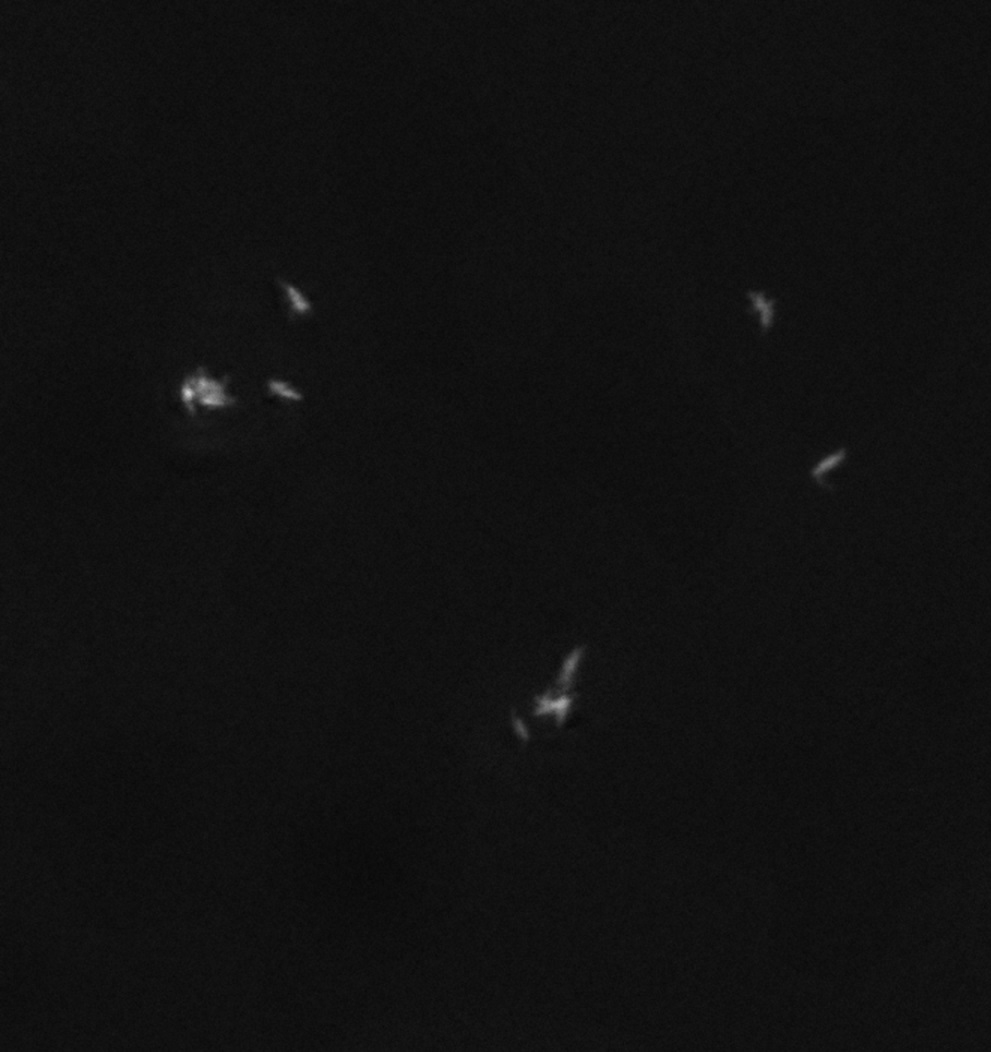

Supplement: Figure 2—source data 1. [file elife-84070-fig2-data1.zip › Figure 2 source data 1/P62/P62 ESX 24h GFP.tif]

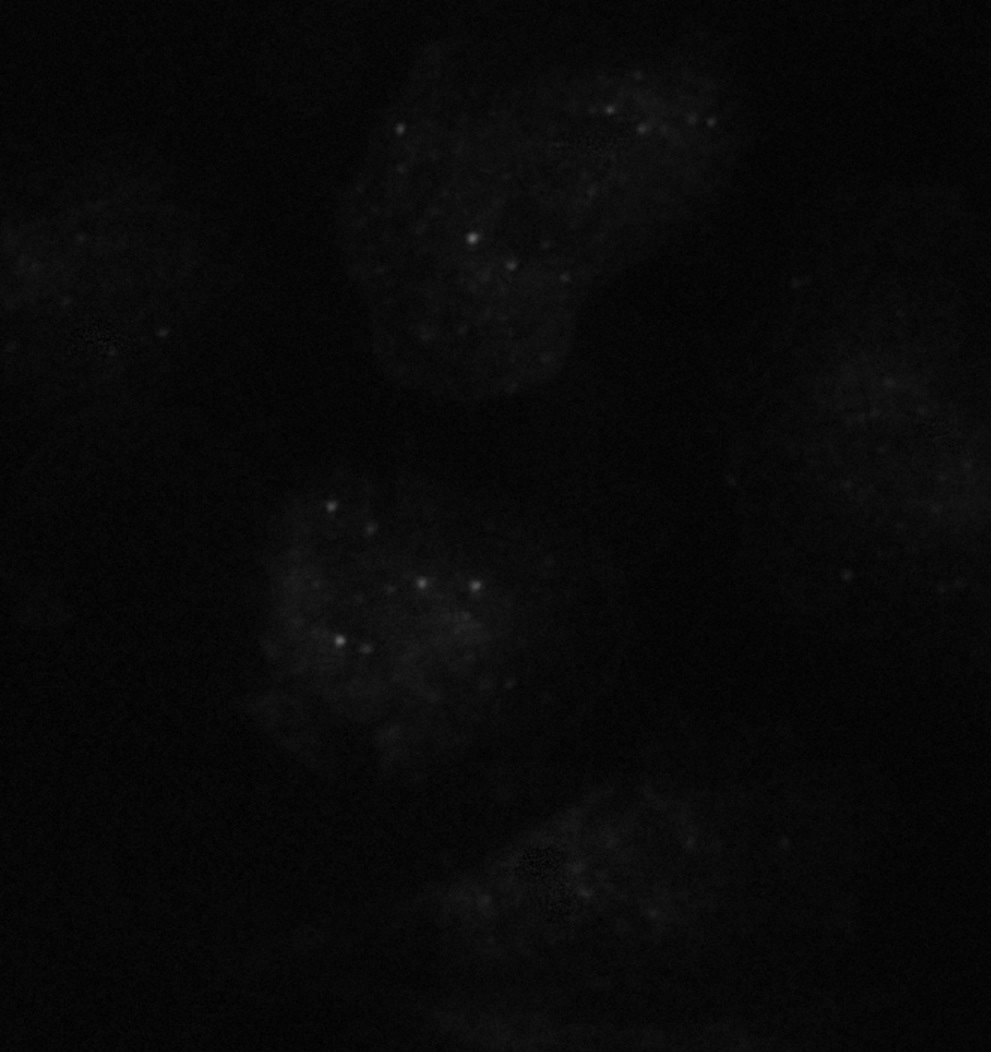

Supplement: Figure 2—source data 1. [file elife-84070-fig2-data1.zip › Figure 2 source data 1/P62/P62 ESX 72h AF647.tif]

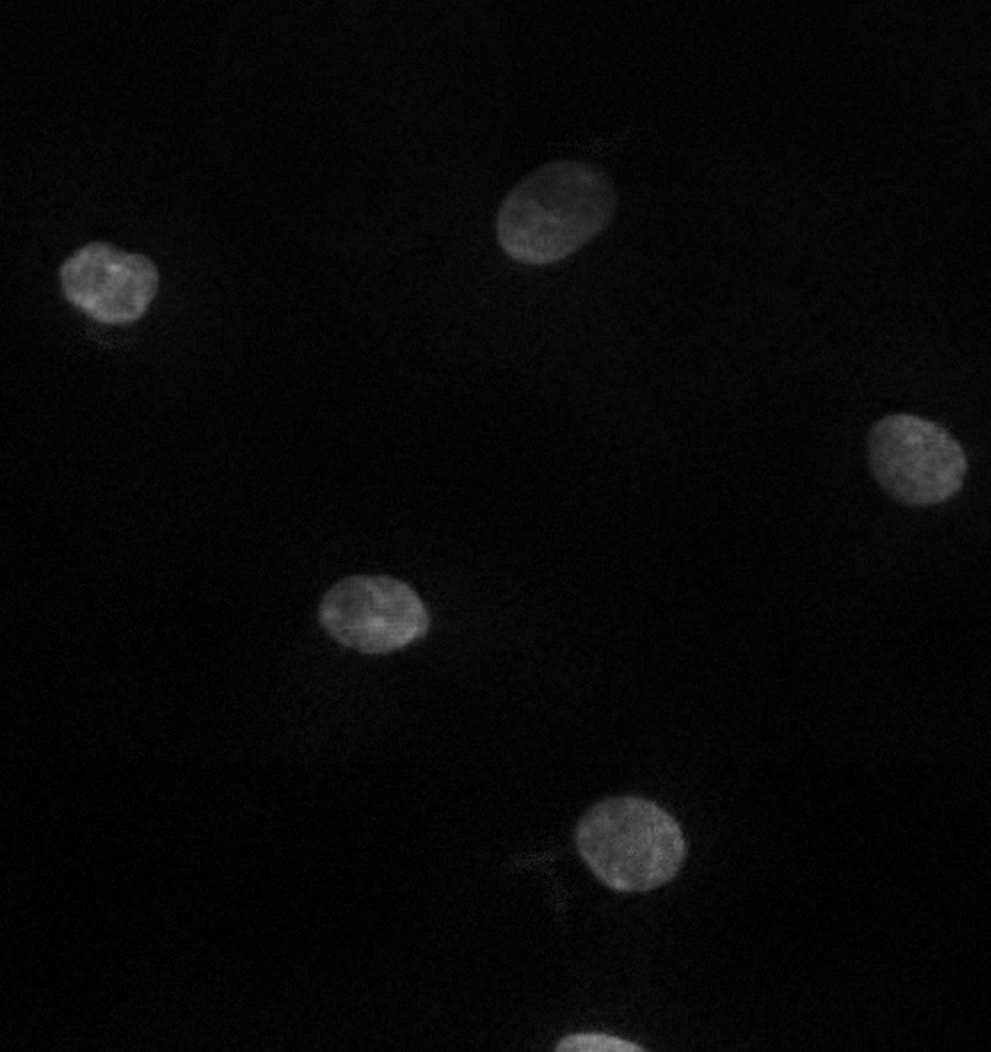

Supplement: Figure 2—source data 1. [file elife-84070-fig2-data1.zip › Figure 2 source data 1/P62/P62 ESX 72h DAPI.tif]

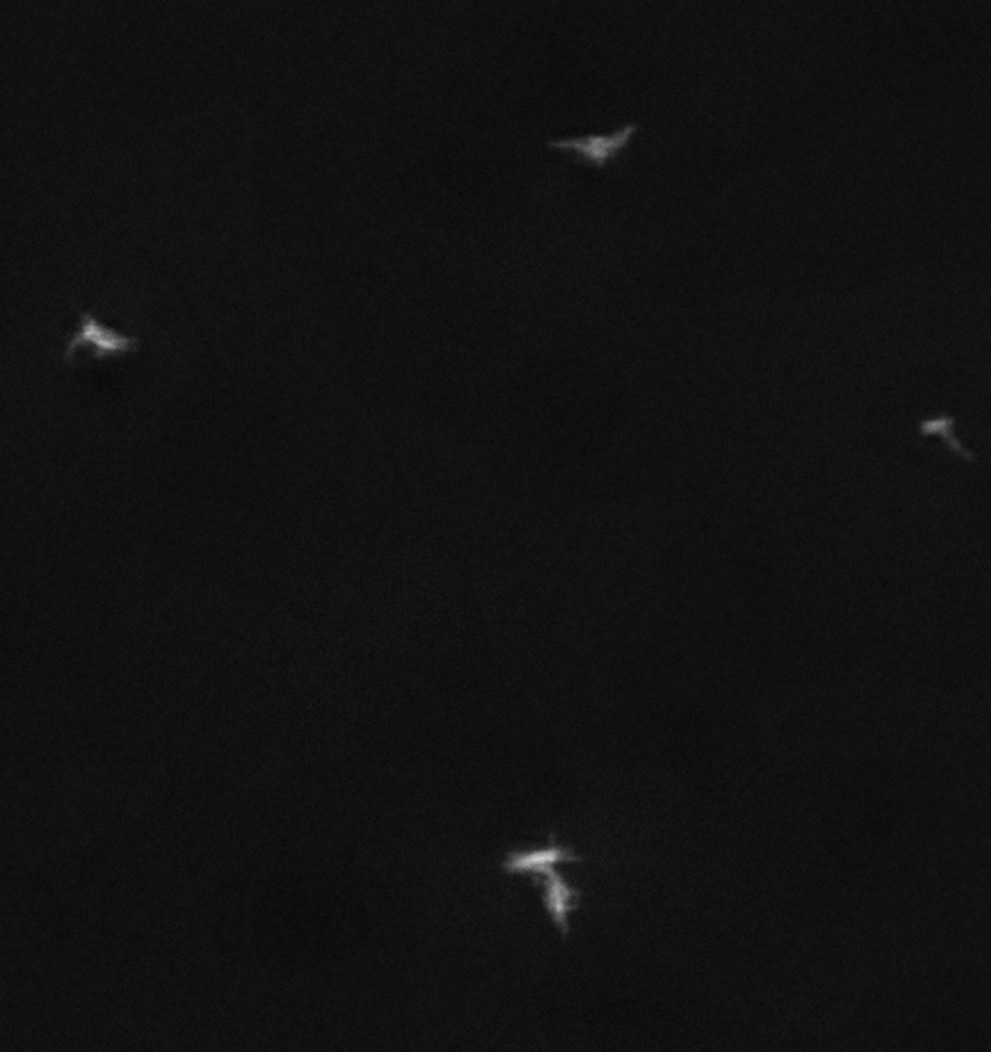

Supplement: Figure 2—source data 1. [file elife-84070-fig2-data1.zip › Figure 2 source data 1/P62/P62 ESX 72h GFP.tif]

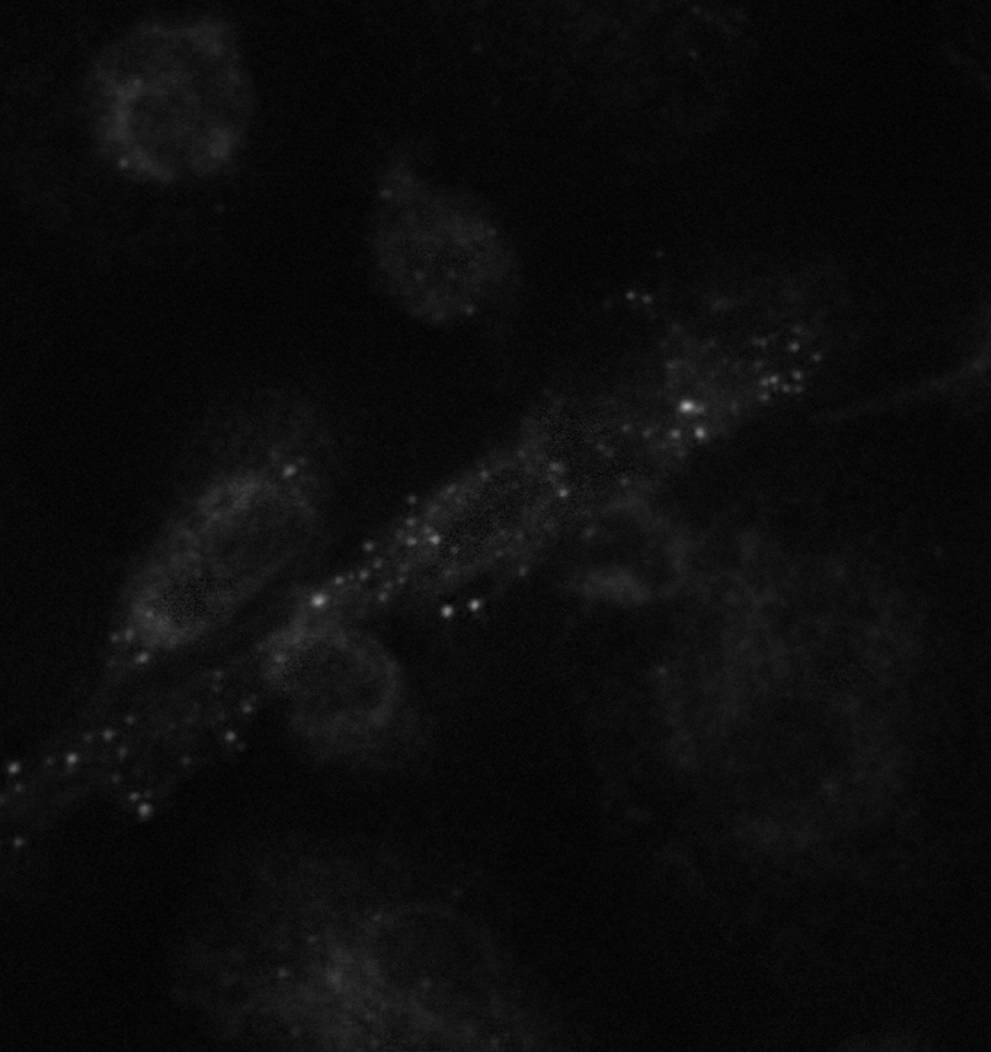

Supplement: Figure 2—source data 1. [file elife-84070-fig2-data1.zip › Figure 2 source data 1/P62/P62 ESX 120h AF647.tif]

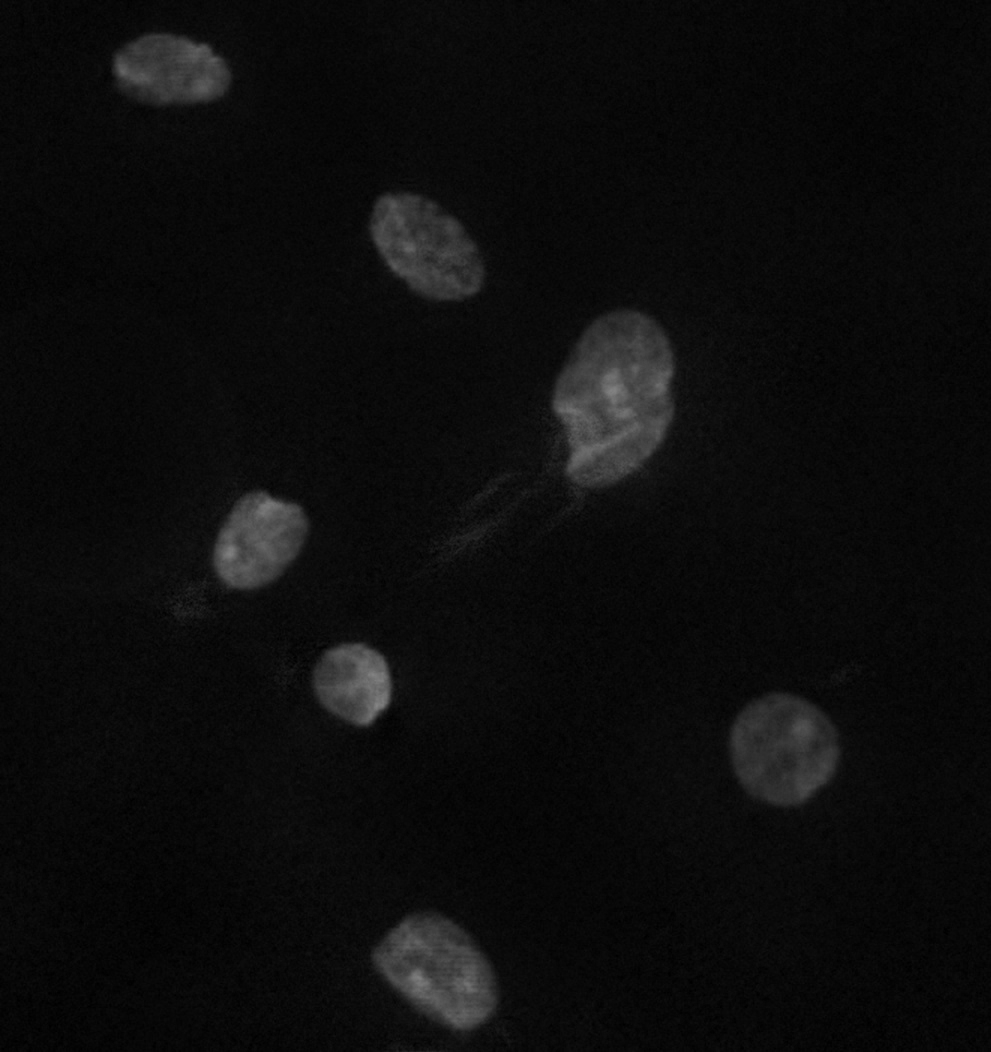

Supplement: Figure 2—source data 1. [file elife-84070-fig2-data1.zip › Figure 2 source data 1/P62/P62 ESX 120h DAPI.tif]

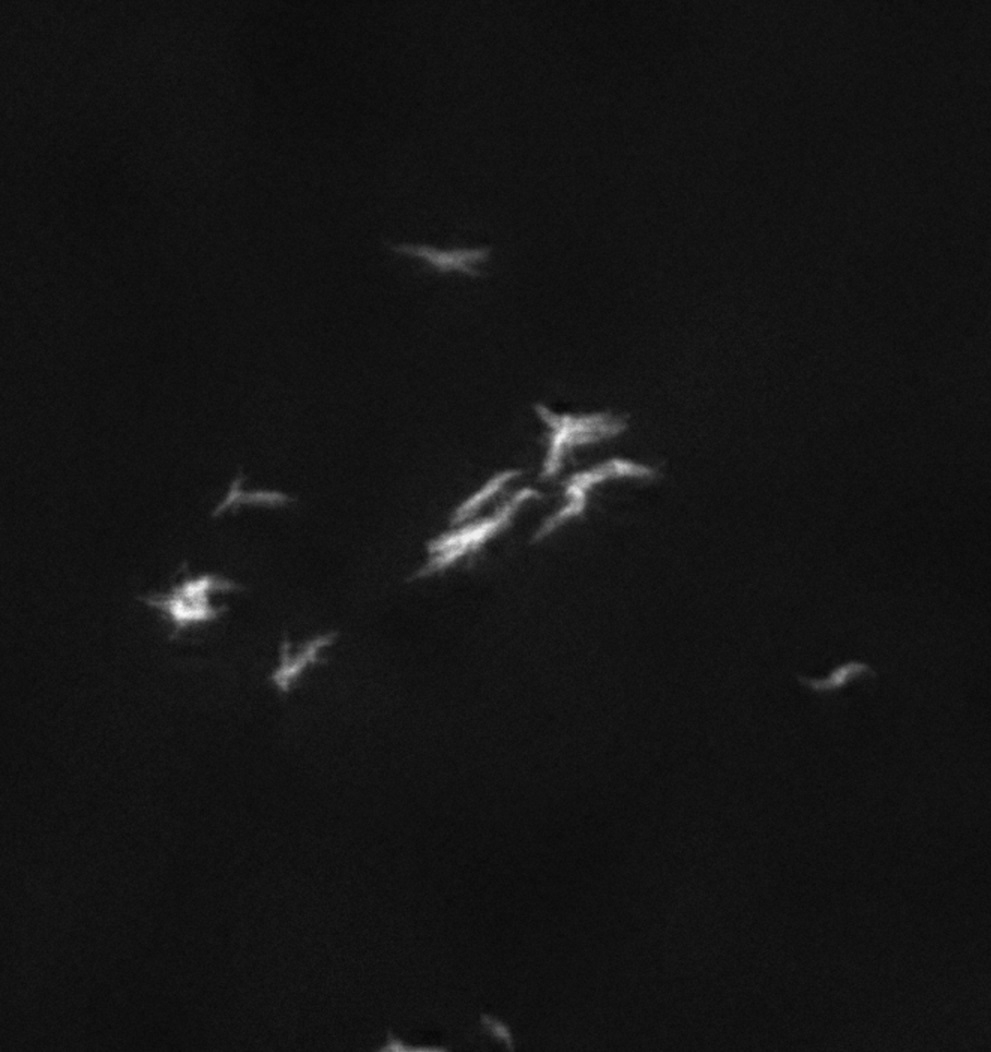

Supplement: Figure 2—source data 1. [file elife-84070-fig2-data1.zip › Figure 2 source data 1/P62/P62 ESX 120h GFP.tif]

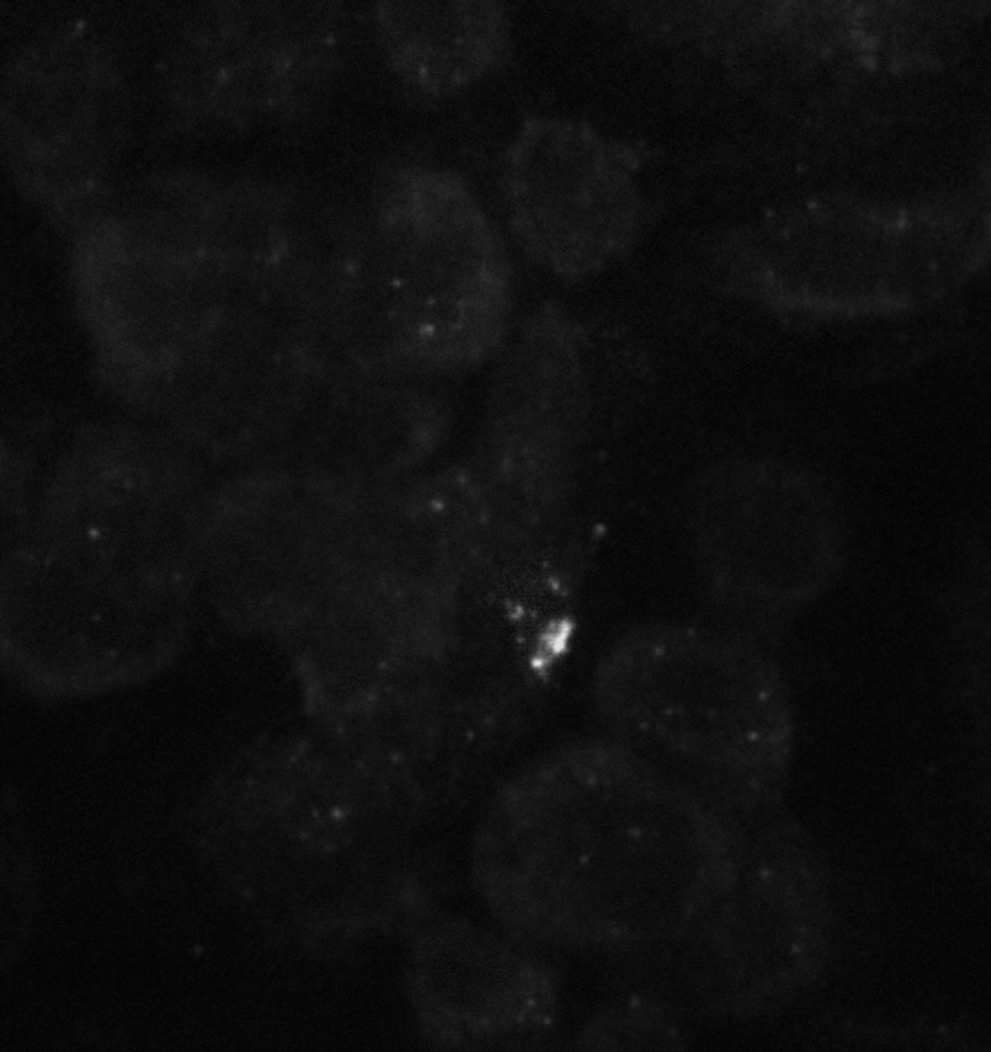

Supplement: Figure 2—source data 1. [file elife-84070-fig2-data1.zip › Figure 2 source data 1/P62/P62 WT 24h 2 AF647.tif]

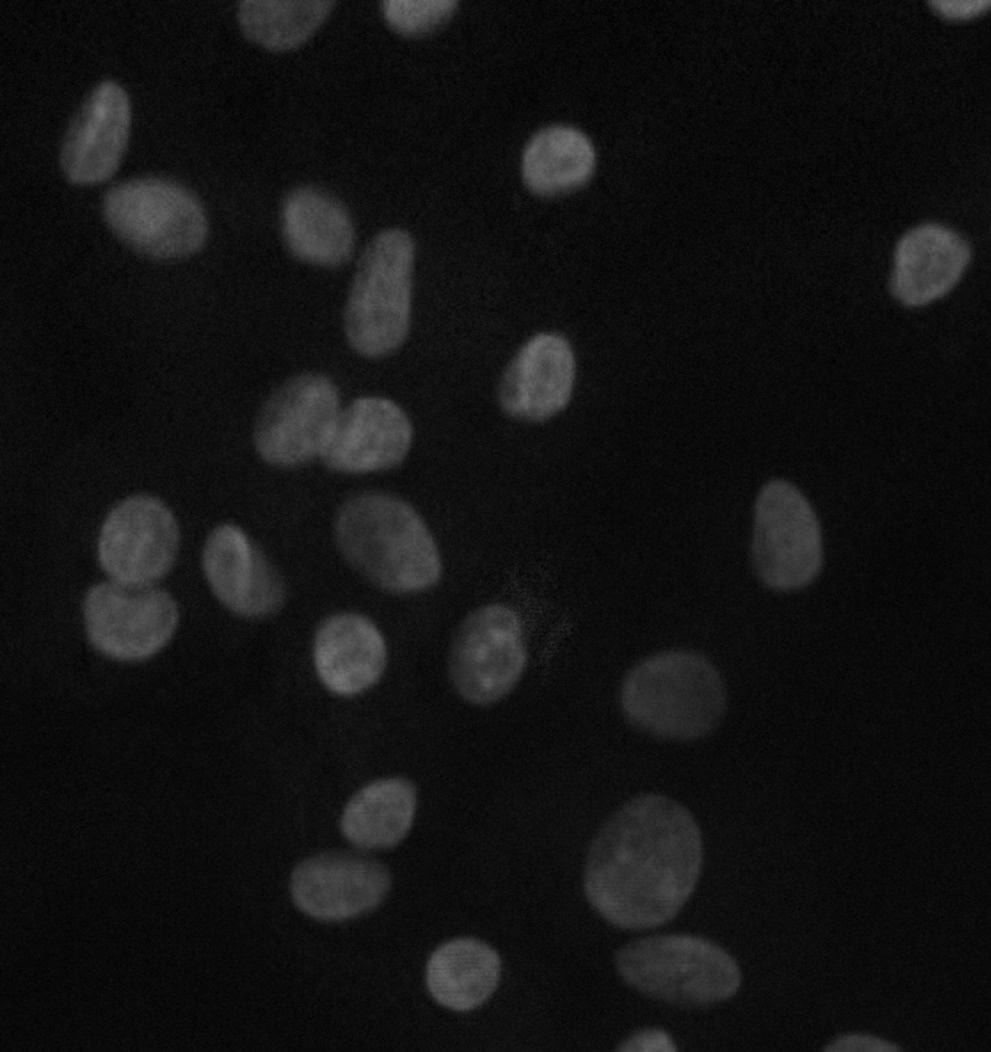

Supplement: Figure 2—source data 1. [file elife-84070-fig2-data1.zip › Figure 2 source data 1/P62/P62 WT 24h 2 DAPI.tif]

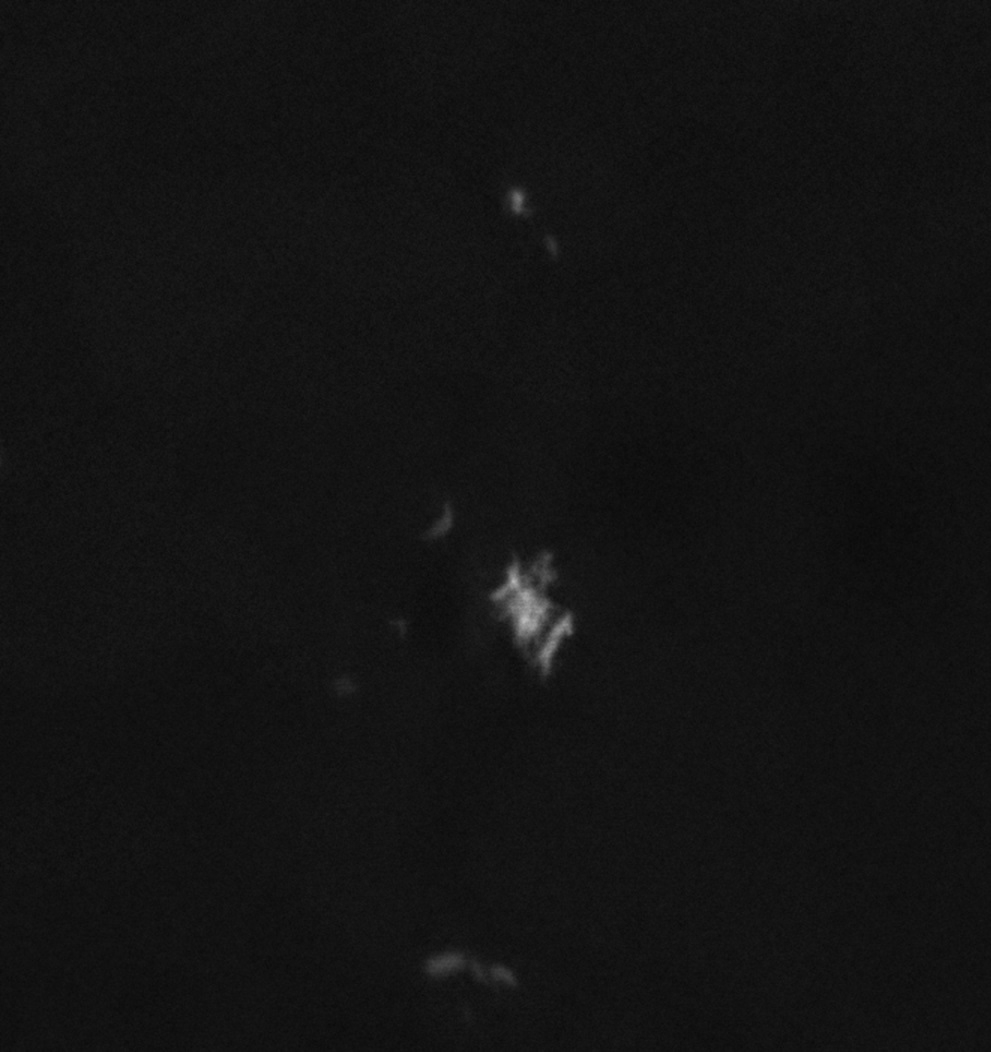

Supplement: Figure 2—source data 1. [file elife-84070-fig2-data1.zip › Figure 2 source data 1/P62/P62 WT 24h 2 GFP.tif]

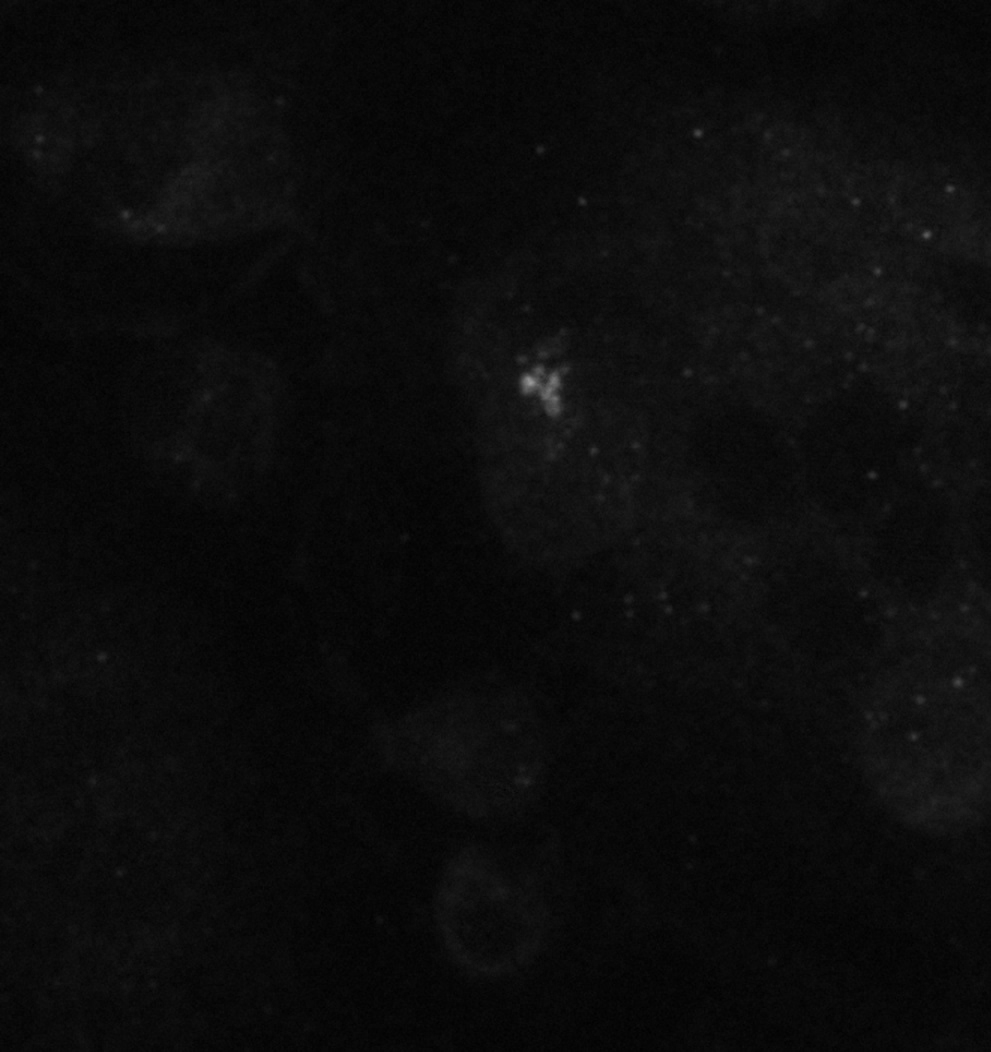

Supplement: Figure 2—source data 1. [file elife-84070-fig2-data1.zip › Figure 2 source data 1/P62/P62 WT 24h AF647.tif]

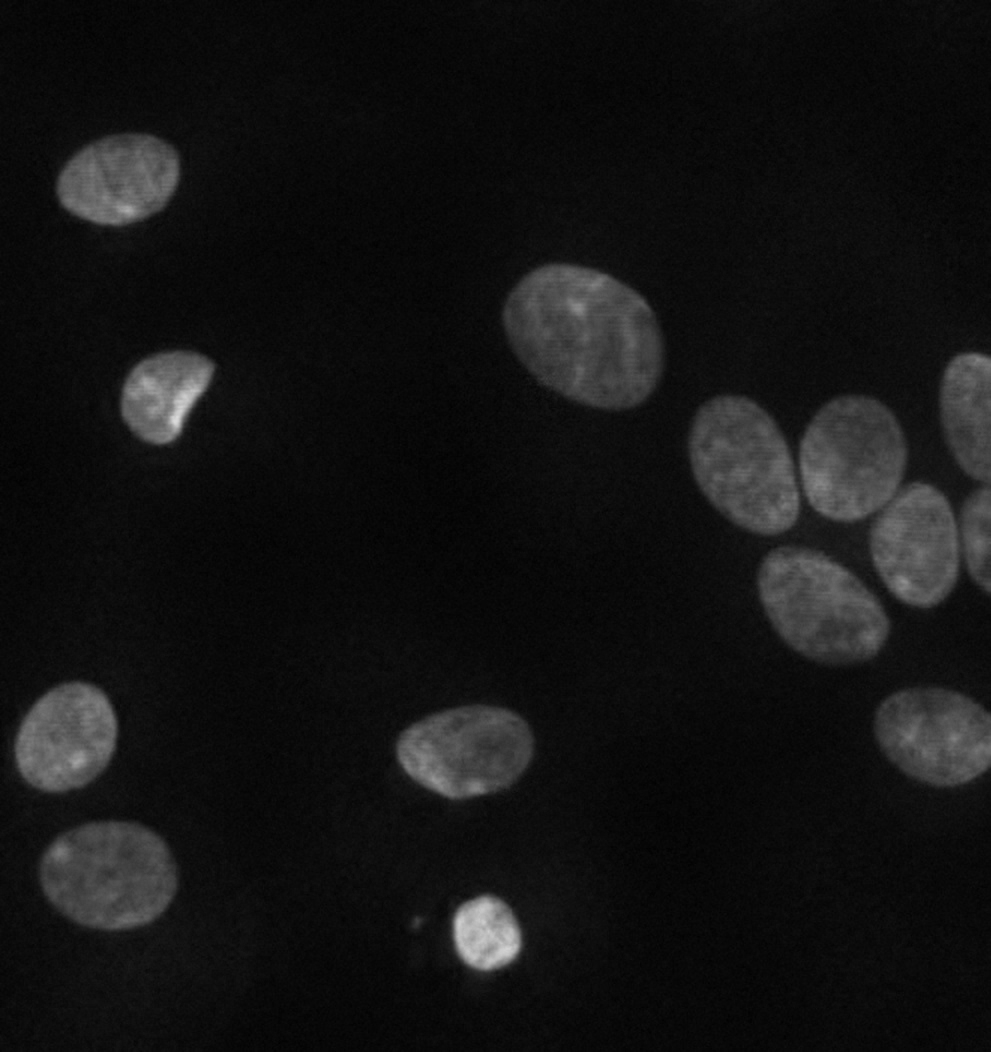

Supplement: Figure 2—source data 1. [file elife-84070-fig2-data1.zip › Figure 2 source data 1/P62/P62 WT 24h DAPI.tif]

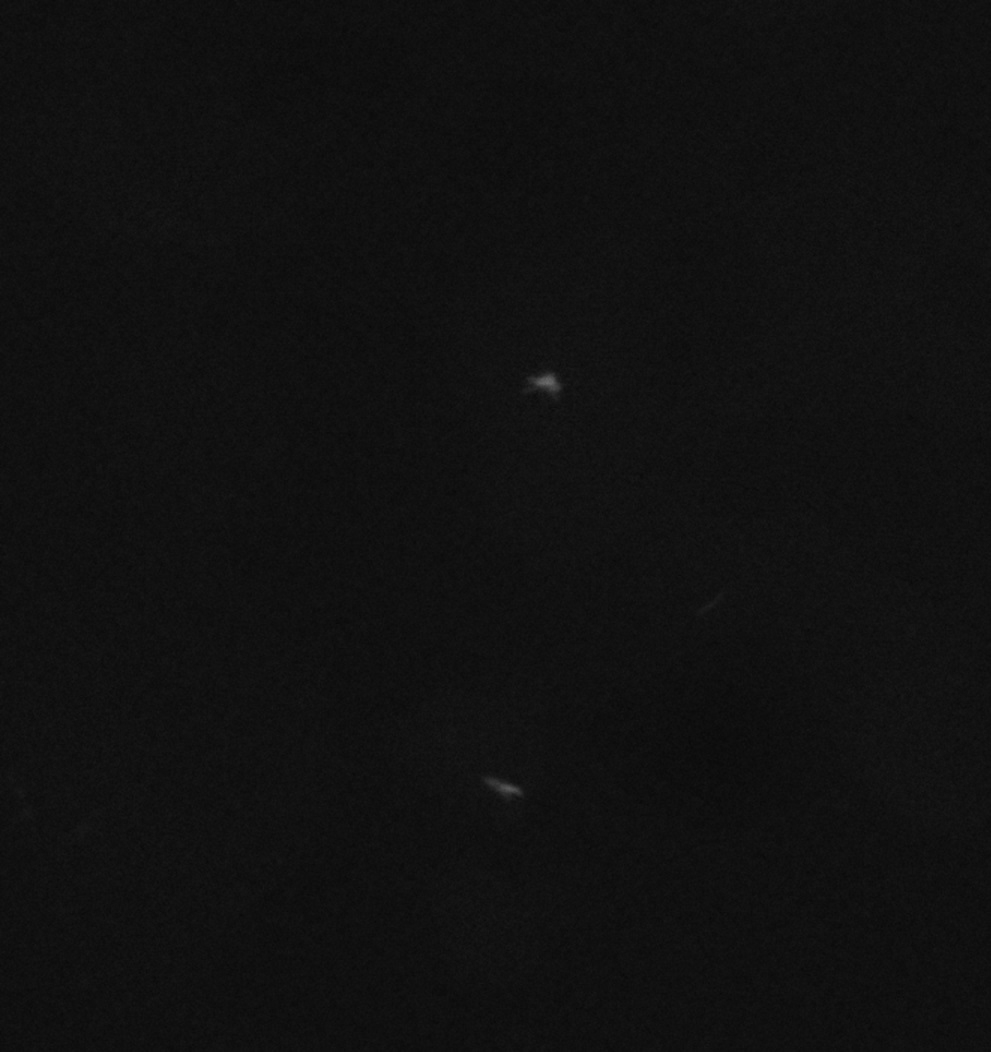

Supplement: Figure 2—source data 1. [file elife-84070-fig2-data1.zip › Figure 2 source data 1/P62/P62 WT 24h GFP.tif]

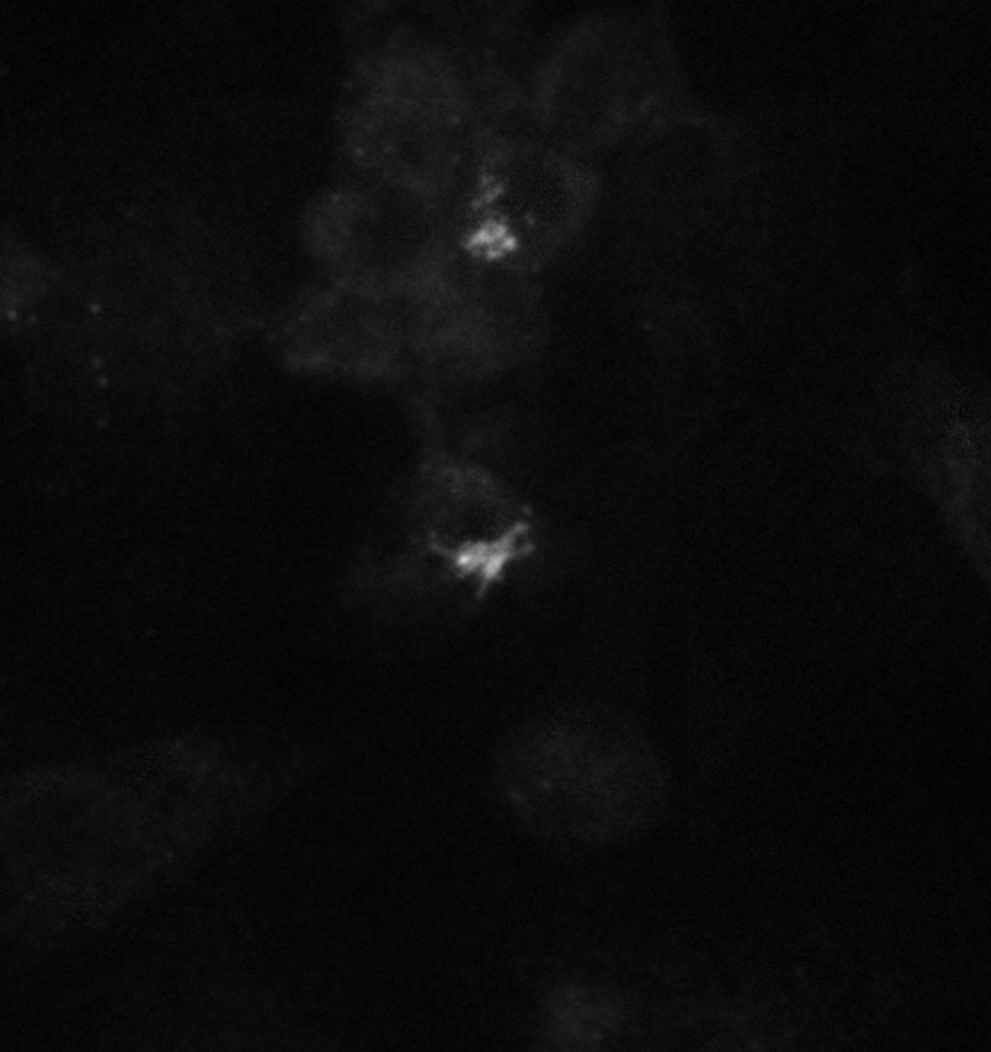

Supplement: Figure 2—source data 1. [file elife-84070-fig2-data1.zip › Figure 2 source data 1/P62/P62 WT 72h 2 AF647.tif]

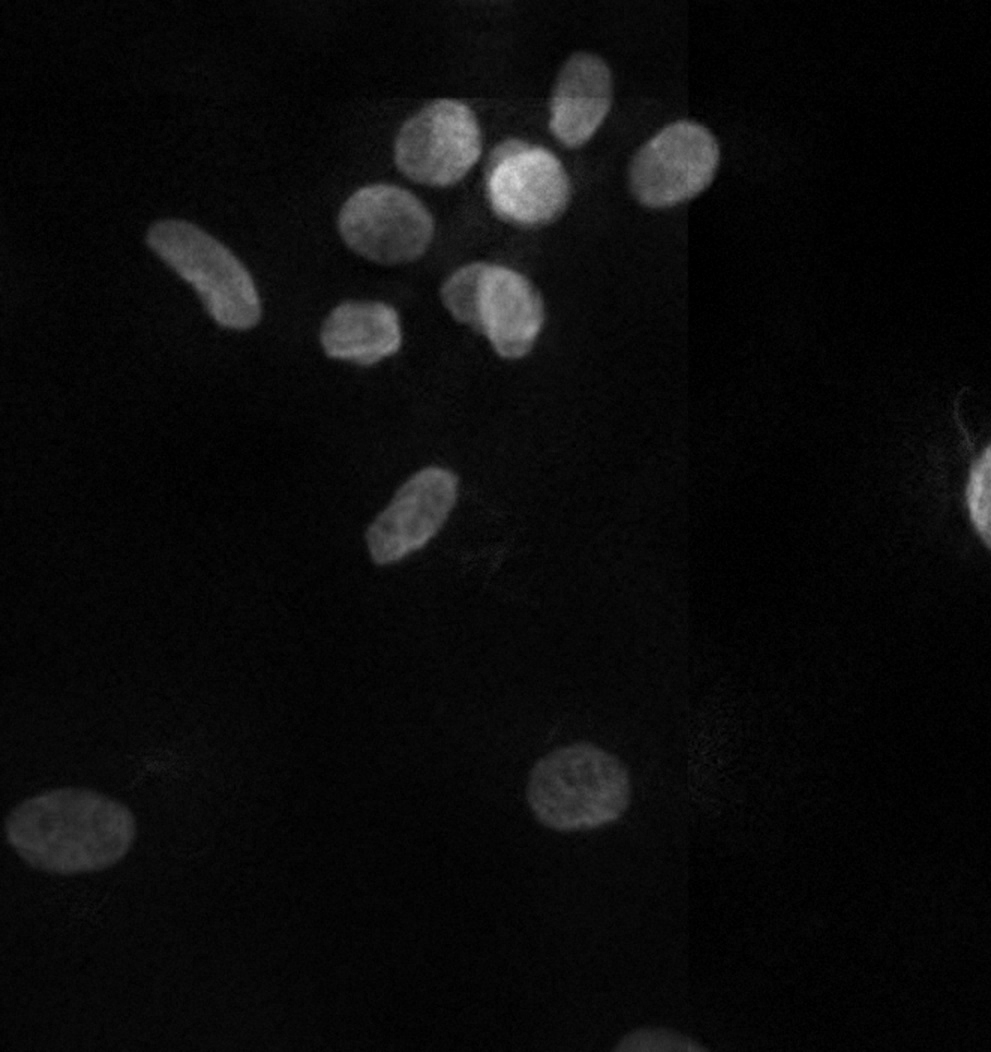

Supplement: Figure 2—source data 1. [file elife-84070-fig2-data1.zip › Figure 2 source data 1/P62/P62 WT 72h 2 DAPI.tif]

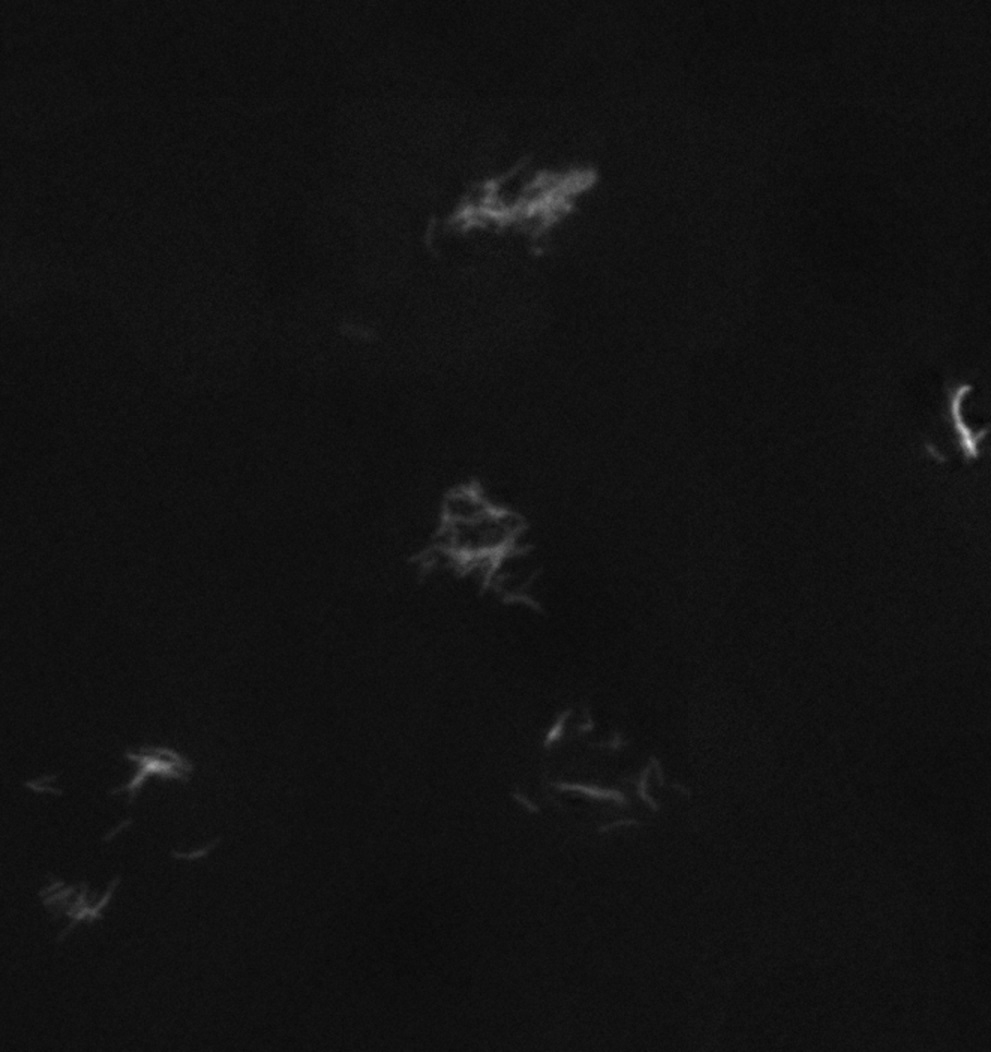

Supplement: Figure 2—source data 1. [file elife-84070-fig2-data1.zip › Figure 2 source data 1/P62/P62 WT 72h 2 GFP.tif]

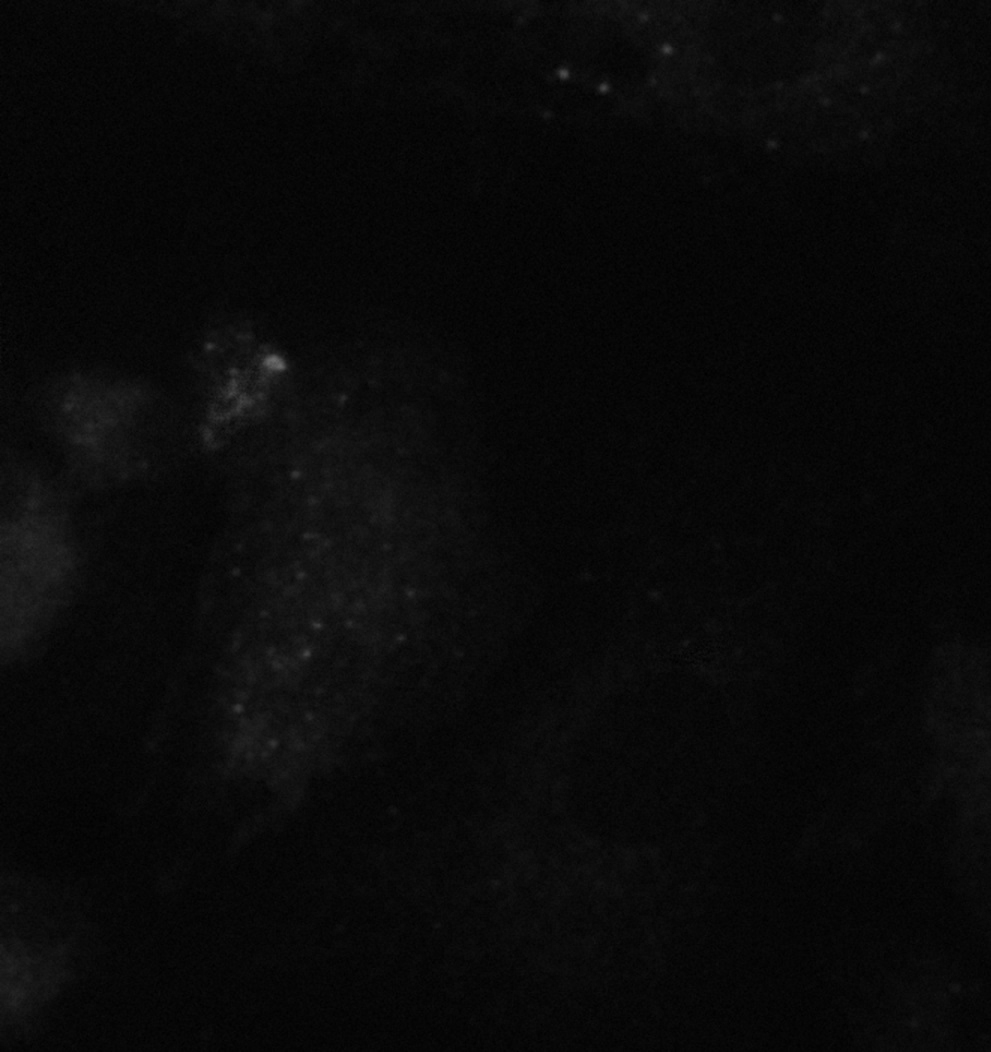

Supplement: Figure 2—source data 1. [file elife-84070-fig2-data1.zip › Figure 2 source data 1/P62/P62 WT 72h AF647.tif]

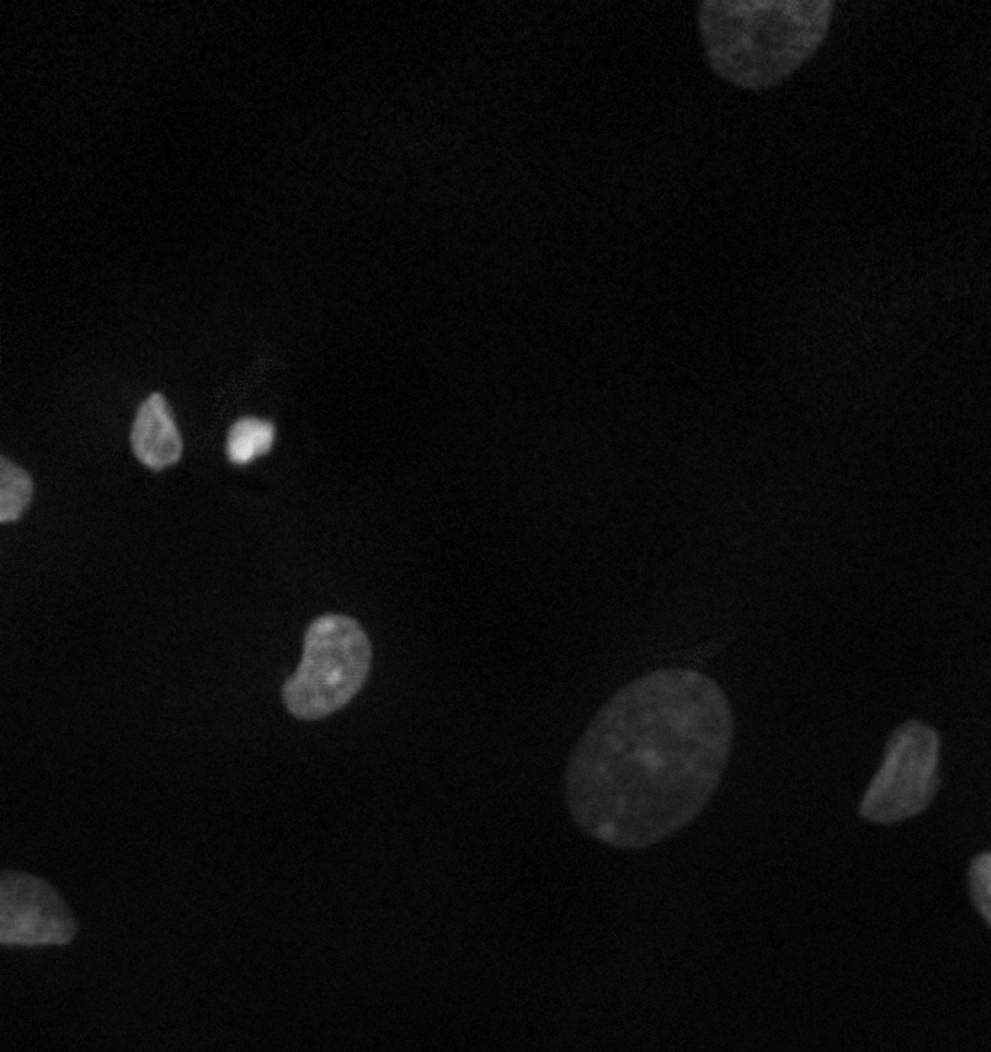

Supplement: Figure 2—source data 1. [file elife-84070-fig2-data1.zip › Figure 2 source data 1/P62/P62 WT 72h DAPI.tif]

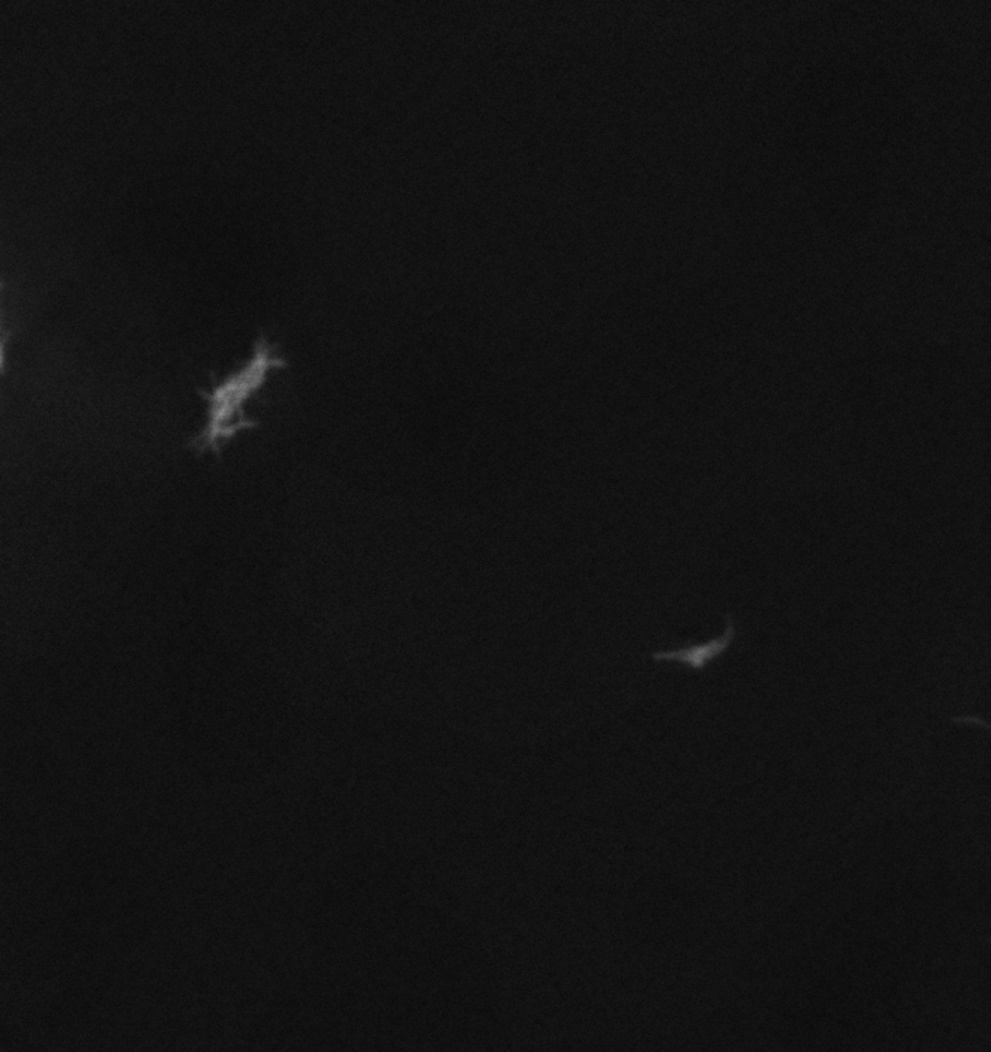

Supplement: Figure 2—source data 1. [file elife-84070-fig2-data1.zip › Figure 2 source data 1/P62/P62 WT 72h GFP.tif]

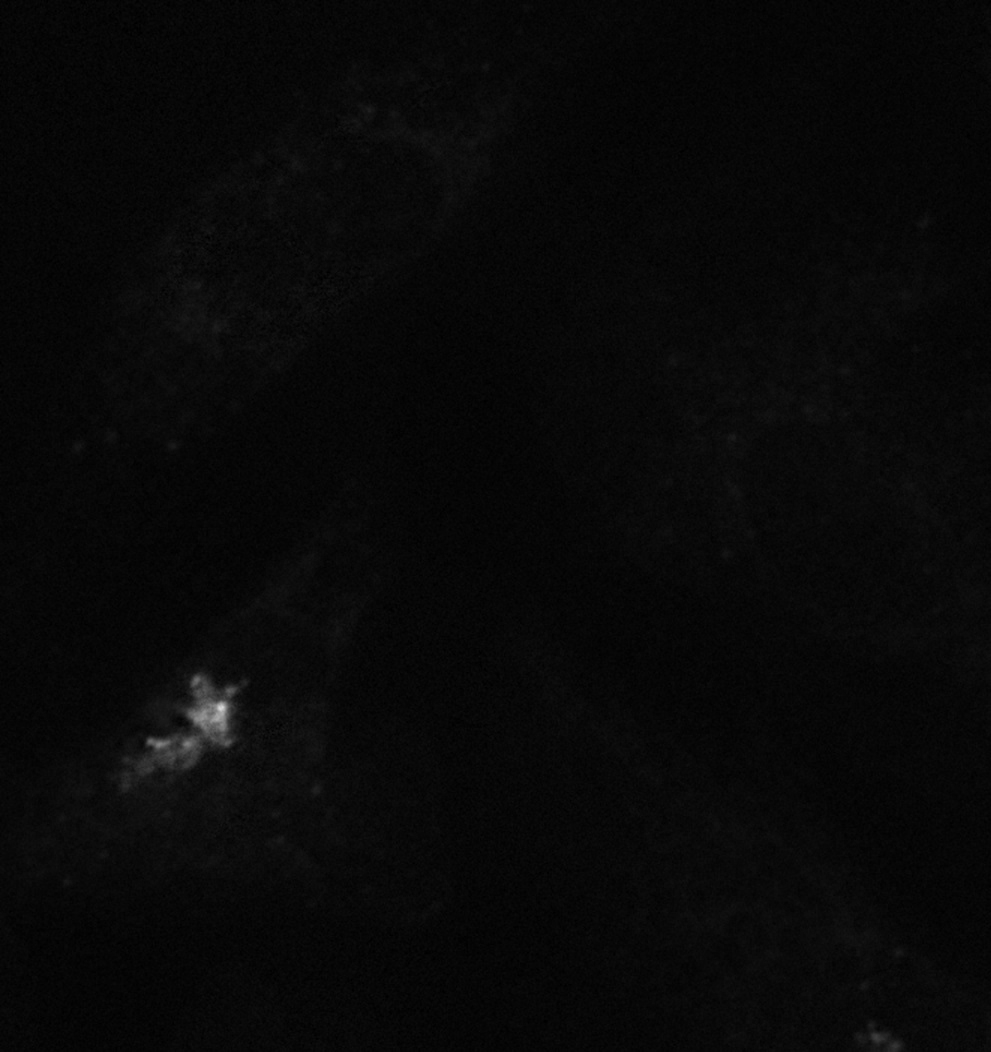

Supplement: Figure 2—source data 1. [file elife-84070-fig2-data1.zip › Figure 2 source data 1/P62/P62 WT 120h 2 AF647.tif]

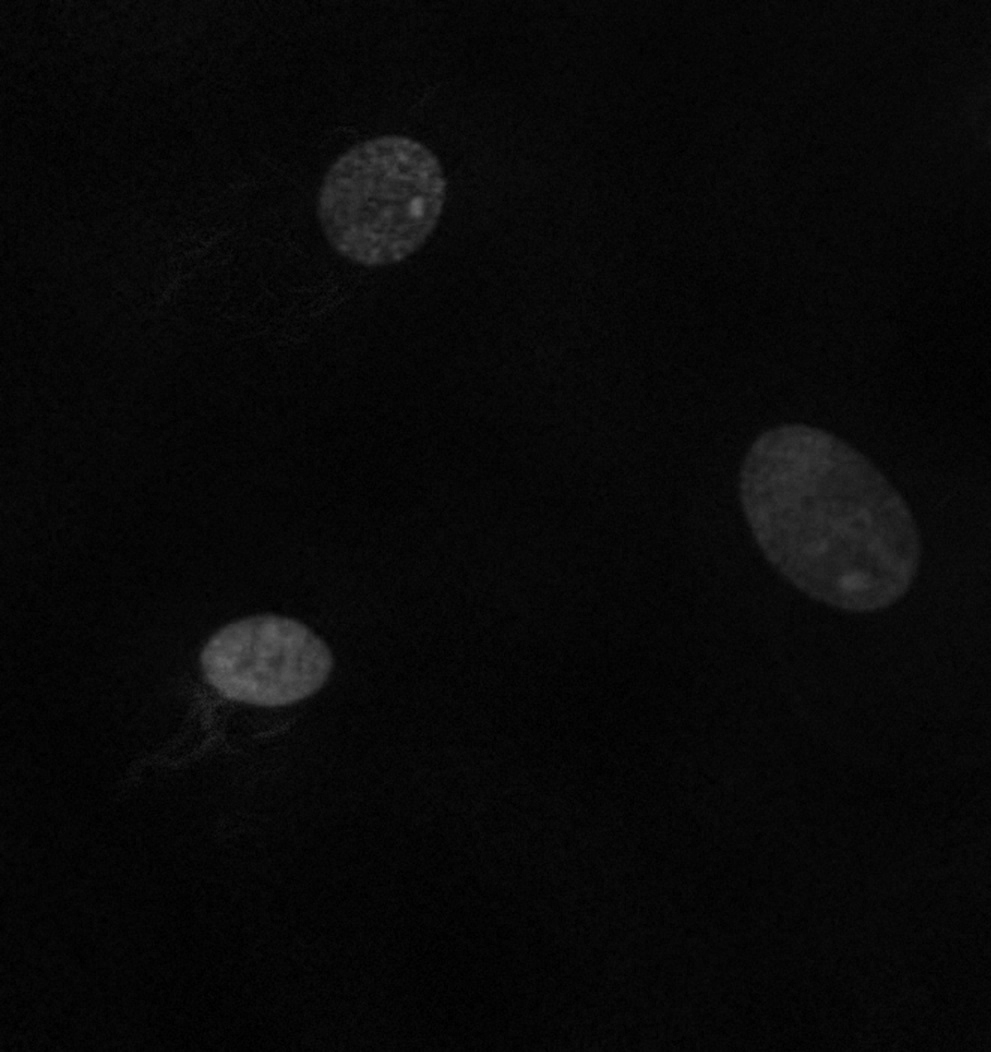

Supplement: Figure 2—source data 1. [file elife-84070-fig2-data1.zip › Figure 2 source data 1/P62/P62 WT 120h 2 DAPI.tif]

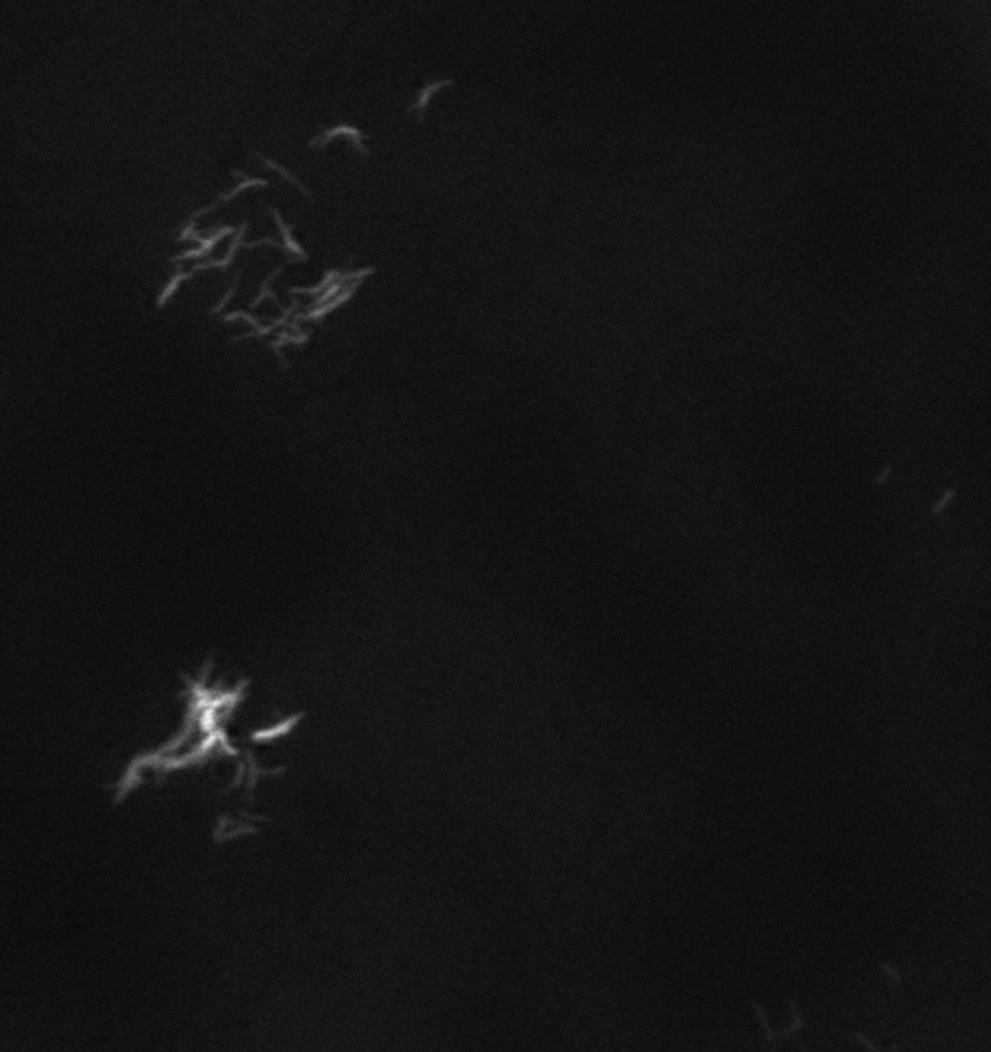

Supplement: Figure 2—source data 1. [file elife-84070-fig2-data1.zip › Figure 2 source data 1/P62/P62 WT 120h 2 GFP.tif]

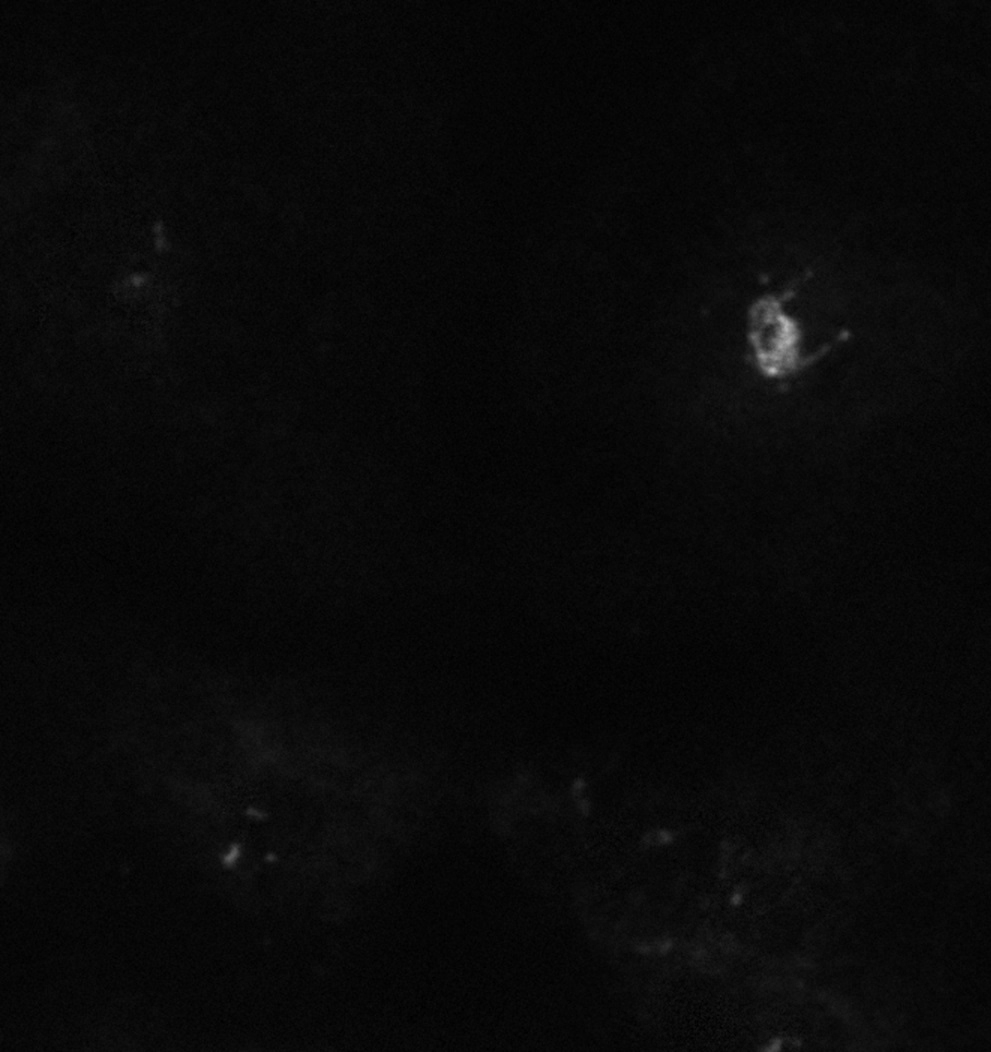

Supplement: Figure 2—source data 1. [file elife-84070-fig2-data1.zip › Figure 2 source data 1/P62/P62 WT 120h AF647.tif]

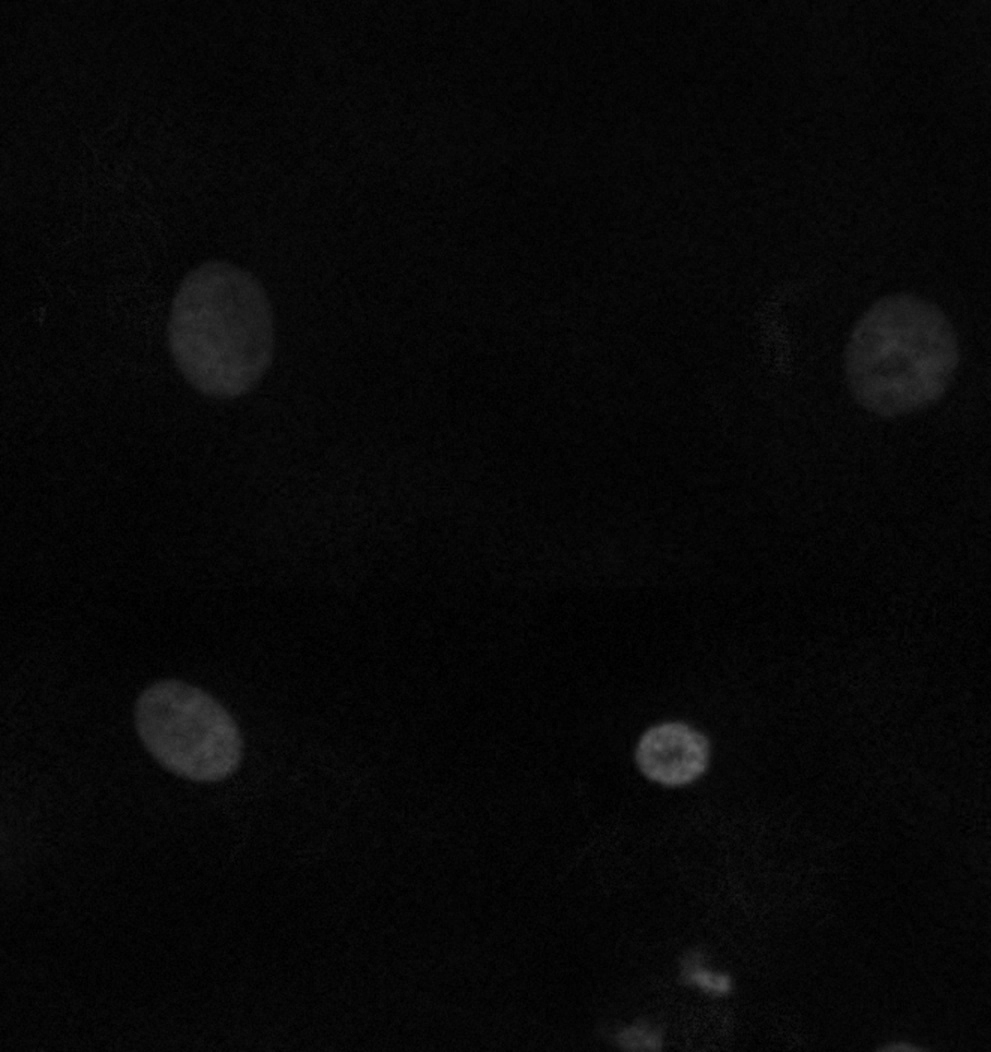

Supplement: Figure 2—source data 1. [file elife-84070-fig2-data1.zip › Figure 2 source data 1/P62/P62 WT 120h DAPI.tif]

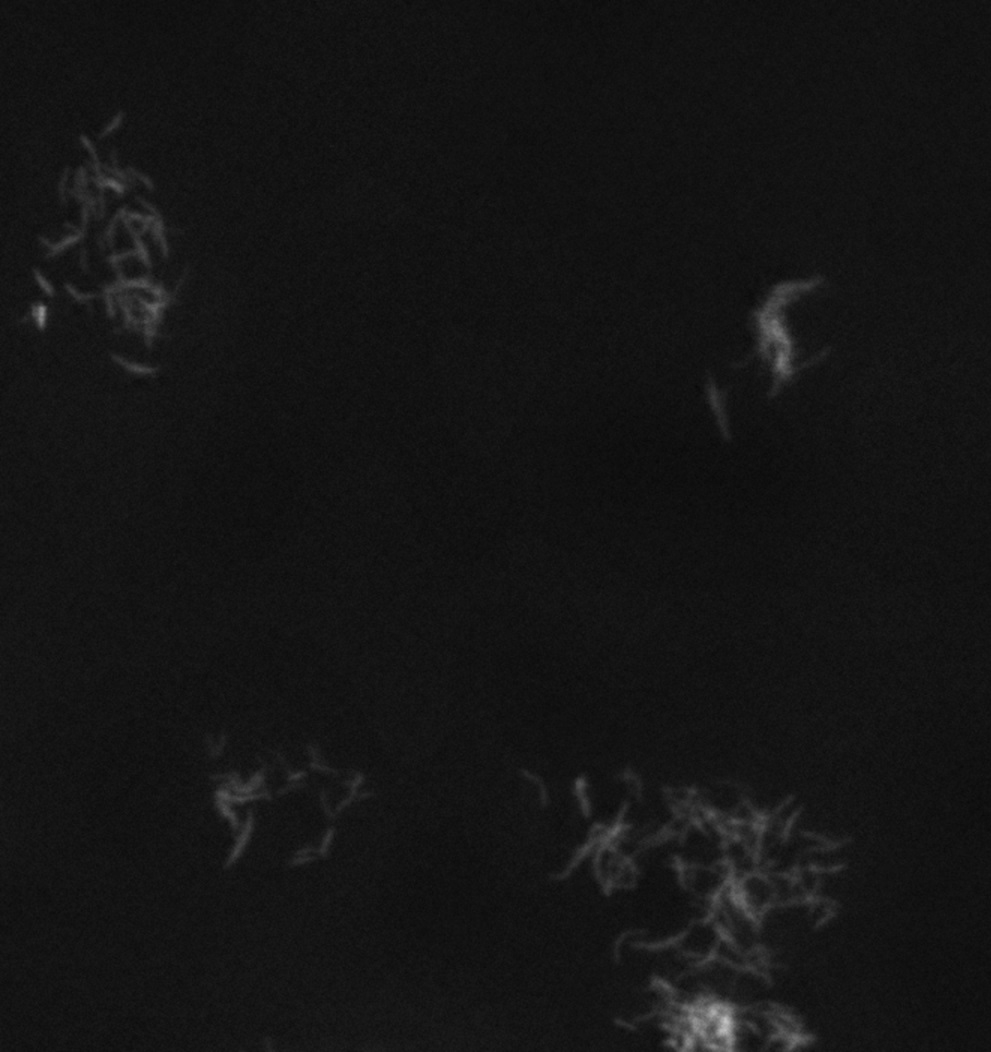

Supplement: Figure 2—source data 1. [file elife-84070-fig2-data1.zip › Figure 2 source data 1/P62/P62 WT 120h GFP.tif]

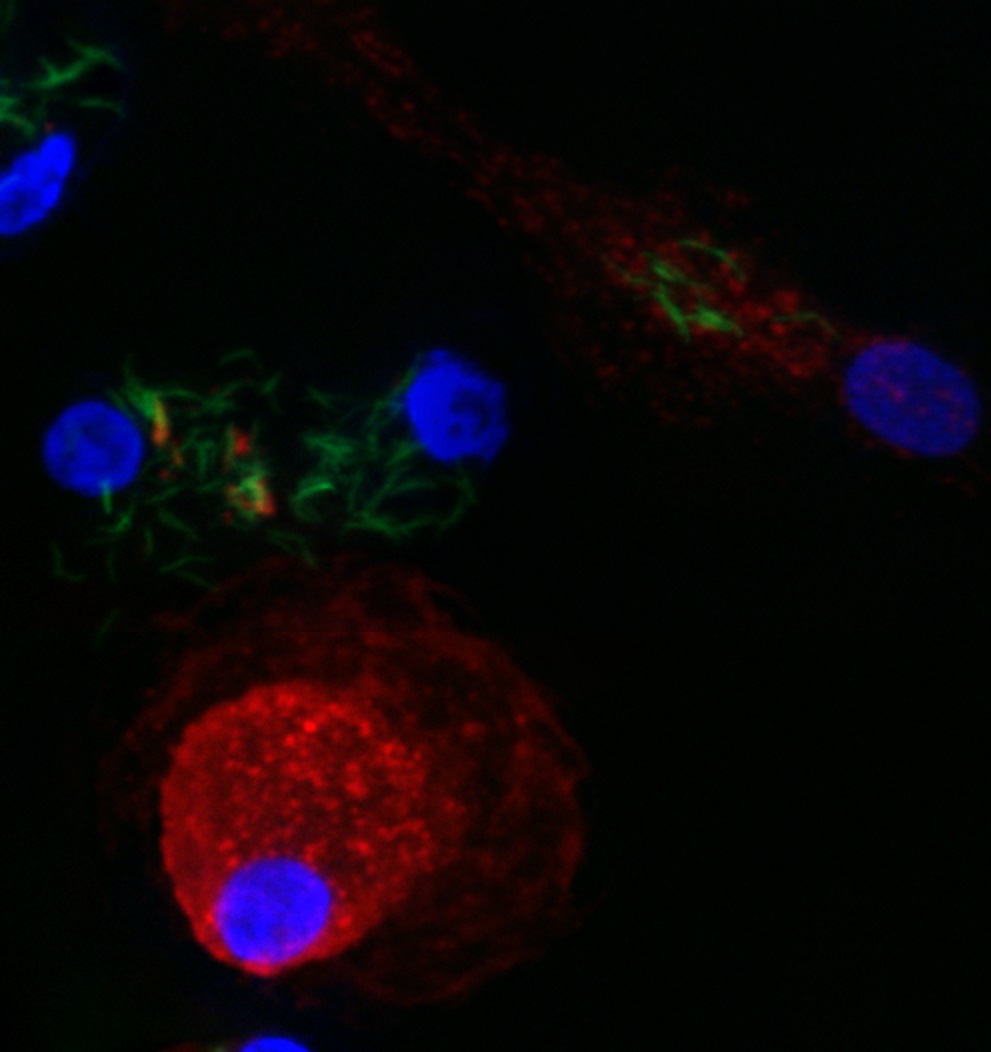

Supplement: Figure 2—source data 1. [file elife-84070-fig2-data1.zip › Figure 2 source data 1/Secondary ctrl/Gal3 +primary no scalebar.JPG]

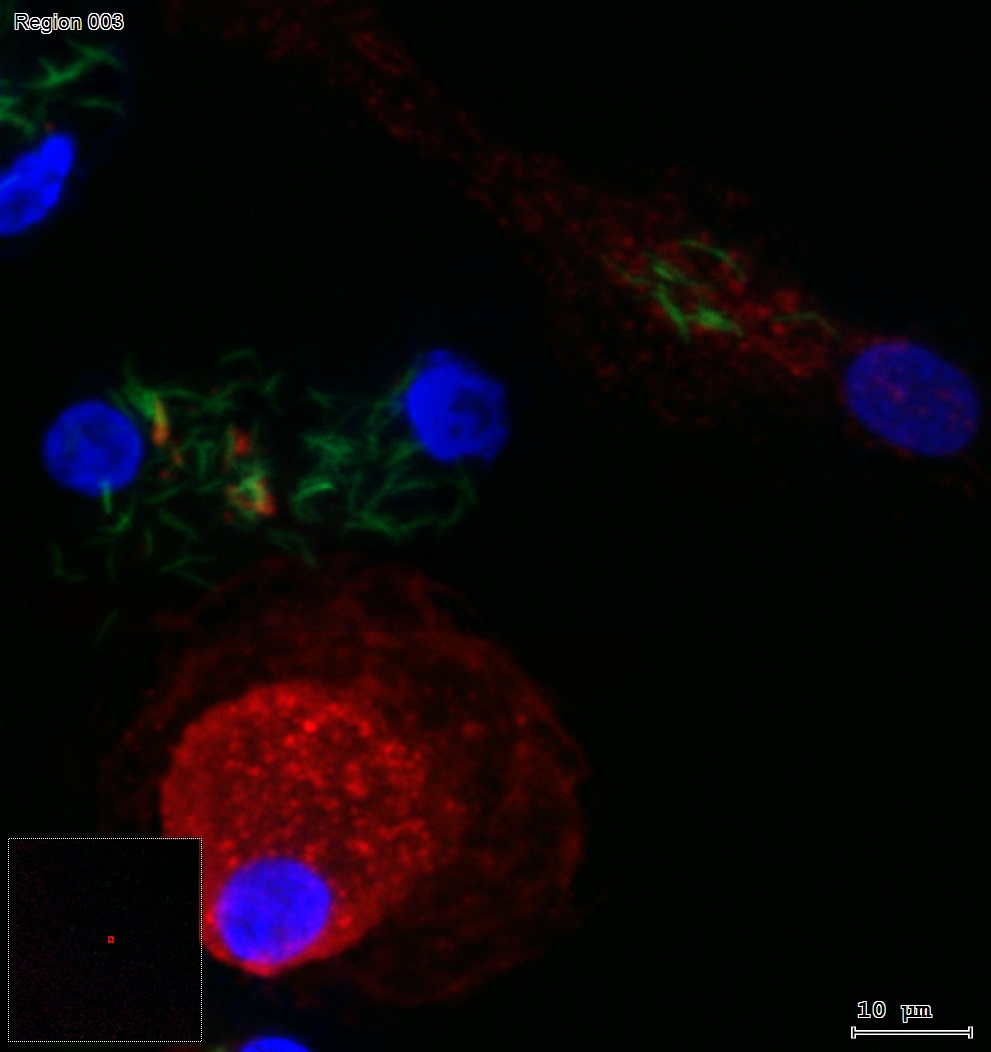

Supplement: Figure 2—source data 1. [file elife-84070-fig2-data1.zip › Figure 2 source data 1/Secondary ctrl/Gal3 +primary.JPG]

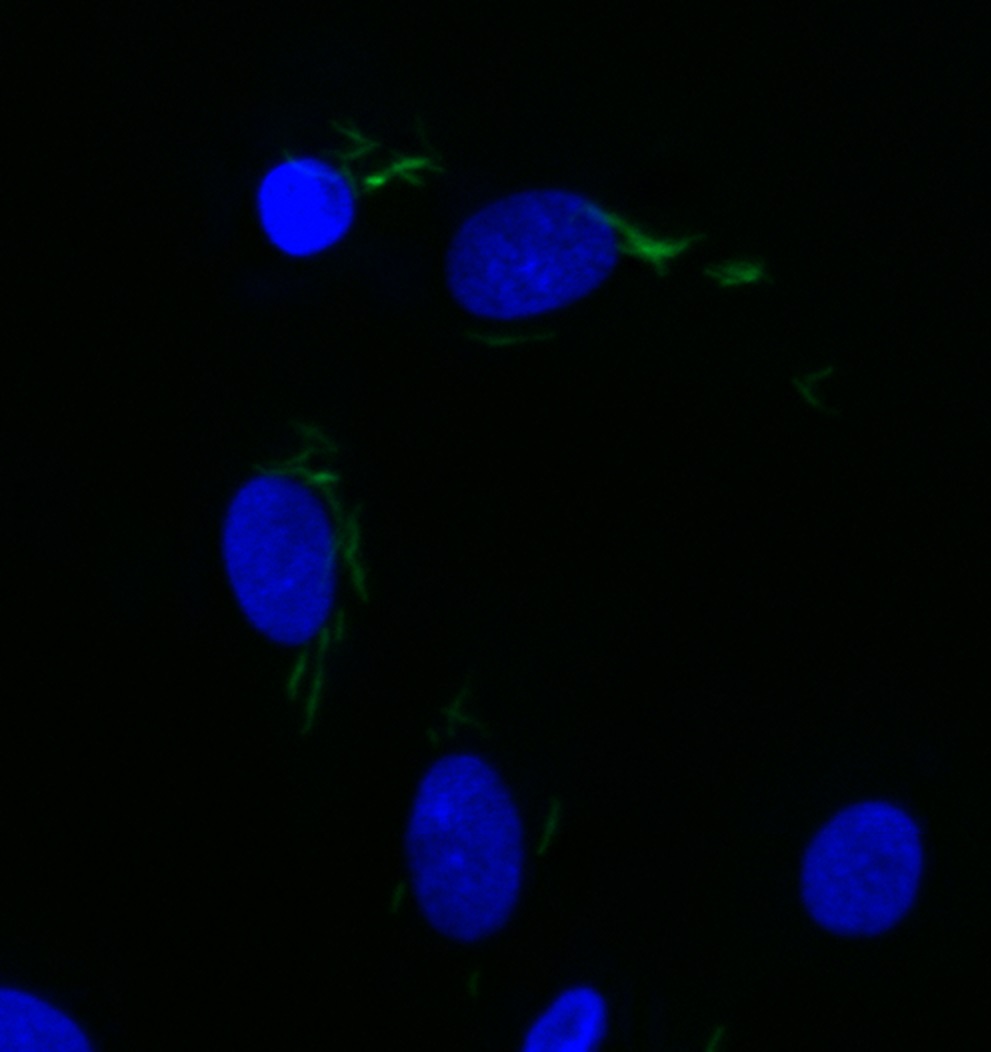

Supplement: Figure 2—source data 1. [file elife-84070-fig2-data1.zip › Figure 2 source data 1/Secondary ctrl/Gal3 secondary_only.JPG]

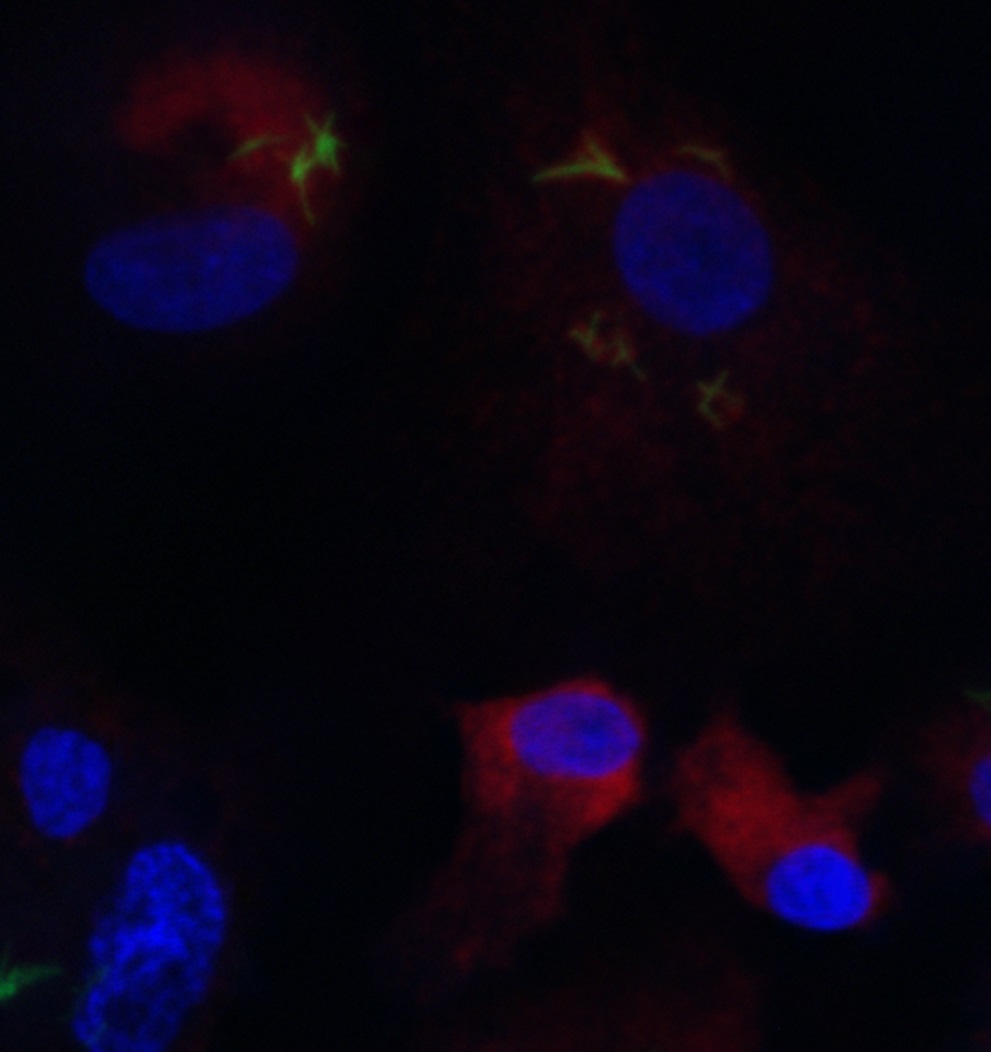

Supplement: Figure 2—source data 1. [file elife-84070-fig2-data1.zip › Figure 2 source data 1/Secondary ctrl/LAMP-1 +primary.JPG]
